# Exploring the sequence-function space of microbial fucosidases

**DOI:** 10.1038/s42004-024-01212-4

**Published:** 2024-06-18

**Authors:** Ana Martínez Gascueña, Haiyang Wu, Rui Wang, C. David Owen, Pedro J. Hernando, Serena Monaco, Matthew Penner, Ke Xing, Gwenaelle Le Gall, Richard Gardner, Didier Ndeh, Paulina A. Urbanowicz, Daniel I. R. Spencer, Martin Walsh, Jesus Angulo, Nathalie Juge

**Affiliations:** 1 The Gut Microbes and Health Institute Strategic Programme, Quadram Institute Bioscience, Norwich Research Park, Norwich, NR4 7UQ UK; 2 GuangDong Engineering Technology Research Center of Enzyme and Biocatalysis, Institute of Biological and Medical Engineering, Guangdong Academy of Sciences, Guangzhou, China; 3 Beijing Key Lab of Traffic Data Analysis and Mining, Beijing Jiaotong University, Beijing, China; 4 Collaborative Innovation Center of Railway Traffic Safety, Beijing Jiaotong University, Beijing, China; 5 School of Computer and Information Technology, Beijing Jiaotong University, Beijing, China; 6 Diamond Light Source Ltd, Diamond House, Harwell Science and Innovation Campus, Didcot, OX11 0FA UK; 7 Research Complex at Harwell, Rutherford Appleton Laboratory, Harwell Oxford, Didcot, OX11 0FA UK; 8 Iceni Glycoscience Ltd., Norwich Research Park, Norwich, NR4 7JG UK; 9 School of Pharmacy, University of East Anglia, Norwich Research Park, Norwich, NR4 7TJ UK; 10 Norwich Medical School, University of East Anglia, Norwich Research Park, Norwich, NR4 7TJ UK; 11 Ludger Ltd, Culham Science Centre, Abingdon, OX14 3EB UK; 12 Departamento de Química Orgánica, Universidad de Sevilla, 41012 Sevilla, Spain; 13 Instituto de Investigaciones Químicas (CSIC-US), 41092 Sevilla, Spain; 14 Present Address: University of Dundee, School of Life Sciences, Dundee, DD1 5EH Scotland UK

**Keywords:** Enzyme mechanisms, Hydrolases, X-ray crystallography, Solution-state NMR, Cheminformatics

## Abstract

Microbial α-l-fucosidases catalyse the hydrolysis of terminal α-l-fucosidic linkages and can perform transglycosylation reactions. Based on sequence identity, α-l-fucosidases are classified in glycoside hydrolases (GHs) families of the carbohydrate-active enzyme database. Here we explored the sequence-function space of GH29 fucosidases. Based on sequence similarity network (SSN) analyses, 15 GH29 α-l-fucosidases were selected for functional characterisation. HPAEC-PAD and LC-FD-MS/MS analyses revealed substrate and linkage specificities for α1,2, α1,3, α1,4 and α1,6 linked fucosylated oligosaccharides and glycoconjugates, consistent with their SSN clustering. The structural basis for the substrate specificity of GH29 fucosidase from *Bifidobacterium asteroides* towards α1,6 linkages and FA2G2 *N*-glycan was determined by X-ray crystallography and STD NMR. The capacity of GH29 fucosidases to carry out transfucosylation reactions with GlcNAc and 3FN as acceptors was evaluated by TLC combined with ESI–MS and NMR. These experimental data supported the use of SSN to further explore the GH29 sequence-function space through machine-learning models. Our lightweight protein language models could accurately allocate test sequences in their respective SSN clusters and assign 34,258 non-redundant GH29 sequences into SSN clusters. It is expected that the combination of these computational approaches will be used in the future for the identification of novel GHs with desired specificities.

## Abstract (web summary)

Microbial α-L-fucosidases pertain to the glycoside hydrolases (GHs) family and catalyse the hydrolysis of terminal α-L-fucosidic linkages and transglycosylation reactions, however, prediction of their function directly from protein sequences remains challenging. Here, the authors combine a protein sequence similarity network and protein language model to explore the sequence-function space of GH29 fucosidases and substrate specificity of uncharacterised GH29 sequences.

## Introduction

Carbohydrate-active enzymes (CAZymes) are responsible for the synthesis, breakdown and modification of all carbohydrates on earth. In the sequence-based classification database (www.cazy.org), CAZymes are grouped into families covering enzymes with common folds and enzymatic mechanisms but different substrate specificities^1^. The number of CAZymes and their families is continuously expanding with glycoside hydrolases (GHs) showing an exponential increase driven largely by high-throughput microbial whole-genome and metagenomic sequencing^2^.

This is for example the case of α-l-fucosidases which are classified into GH29, GH95, GH139, GH141, and GH151 families, a majority of which are from microbial sources^3^. Reflecting the high diversity of naturally occurring fucosylated structures, these enzymes show a wide range of substrate and linkage specificity, cleaving the nonreducing terminal α-l-fucose (Fuc) and have numerous biological roles and applications in health and biotechnology^4^.

GH95 fucosidases functionally characterised so far show strict substrate specificity to the terminal Fuc α1,2 Gal linkage and hydrolyse the linkage via an inverting mechanism. The GH139 and GH141 families include one functionally characterised fucosidase targeting specific α-l-fucose motifs in pectin^5^. The GH151 family includes three characterised fucosidases including ALfuk2 from *Paenibacillus thiaminolyticus* targeting α1,2/3/4/6 fucosylated disaccharides^6^ and two other GH151 members which showed weak or no activity towards trisaccharides^7,8^.

In contrast, fucosidases from the GH29 family have been widely studied since 1970s^9^ and are reported to act on a broad range of substrates with hydrolysis proceeding via a retaining mechanism. This family covers fucosidases with substrate specificities against Fucα1,2/3/4/6 motifs. Some of the GH29 fucosidases have relaxed substrate specificities and can act on 4-nitrophenyl α-l-fucopyranoside (pNP-Fuc) (EC 3.2.1.51), while other fucosidases show strict specificity for terminal α-(1,3/4)-fucosyl linkages with little/no activity on pNP-Fuc (EC 3.2.1.111), which led to an attempt to subdivide the GH29 family into substrate-based specificity GH29-A and GH29-B, but more accurate classification is needed^10^. We previously reported the substrate and linkage specificities of fucosidases from the human gut symbiont *Ruminococcus gnavus*, revealing a GH29 fucosidase with the capacity to recognise sialic acid-terminated fucosylated glycans (sialyl Lewis X/A epitopes) and hydrolyse α1,3/4 fucosyl linkages in these substrates without the need to remove sialic acid^11^. In addition, GH29 fucosidases are increasingly being considered as glyco-tools for their capacity to synthesise oligosaccharides by transglycosylation, as reported for AlfB and AlfC from *Lactobacillus casei* BL23^12,13^. According to the CAZy database (last update:2023-10-10), out of 11,285 GH29 sequences, 98 were characterised at the protein level, and 18 have 3D structure information. Given the wide enzymatic diversity within the GH29 family, there is great interest in mining this family for applications^14,15^ and several bioinformatics-based approaches are being tested to better predict substrate specificity and transglycosylation ability of these enzymes^16,17^.

The protein sequence similarity network (SSN) is a well-known method for analysing protein sequences relationships and has been applied in classifying protein families or subfamilies including kinases^18^, CAZymes such as GH16^19^ or GH29 and GH95 fucosidases^11^. Conserved unique peptide pattern (CUPP) is a newly developed approach for CAZyme annotation based on peptide-motif clustering and shows higher sensitivity compared to multiple alignment of full-length protein sequences such as Hidden Markov Model (HMM), InterProScan or dbCAN^20,21^. CUPP has recently been used to provide an overview of the sequence and function diversity of fucosidases across GH29^16^. However, both methods have limitations in delineating enzymes that were not included in the original SSN/CUPP analyses. With numbers of GH29 enzymes growing day by day, it is important to develop bioinformatics approaches to guide the rational selection of these novel enzymes. Recently, protein language models (pLMs) have been developed to extract sequence features as protein representations which can be employed in learning and predicting protein properties such as protein structure or remote homology and classification, leveraging the recent advances of deep learning in natural language processing^22–32^. This approach provides an alternative to multiple sequence alignment (MSA)-based prediction methods requiring mapping query sequences against databases to generate sequence profiles based on the scoring systems or HMMs which are computationally expensive.

Here, we developed an innovative approach for exploring the GH29 sequence-function space by combining SSN clustering of GH29 family with pLMs to expand the range of sequences that can be interrogated for their putative function.

## Results

### Sequence similarity network (SSN) revealed predicted substrate-specificity GH29 fucosidase clusters

**Fig. 1:**
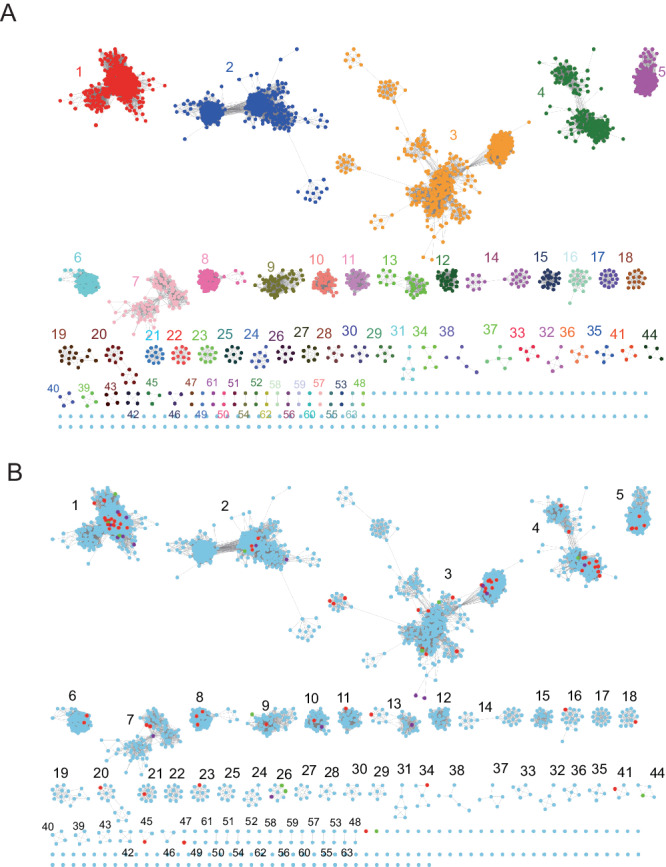
Sequence similarity network (SSN) of the GH29 fucosidase family. **A** The coloured SSN of GH29 family after cluster analysis. **B** The distribution of functionally characterised GH29s in different clusters. Red nodes represent enzymatically characterised GH29s, purple nodes represent structurally characterised GH29s while green nodes represent new GH29 enzymes characterised in this work.

SSN was used to explore the sequence-function space of microbial fucosidases belonging to the GH29 family (www.cazy.org). The SSN is composed of nodes and edges, with each representative node representing a single protein sequence, which is linked with an edge when sharing over 40% sequence identity. The SSN analysis of GH29 amino acid sequences revealed a total of 2,971 representative nodes wired by 141,732 edges. The network was composed of 63 distinct main clusters and 121 singletons defined by cluster analysis utility^33^ (Fig. 1). Clusters 1, 2, and 3 accounted for 54% of the total nodes. Of the 63 clusters analysed, clusters 1-11, 13, 16, 18, 20, 21, 23, 26, 34, 41, 45 and 47 included sequences corresponding to functionally characterised enzymes. Among them, clusters 1, 13, and 45 contained GH29-B enzymes while the remaining clusters belonged to GH29-A apart from cluster 11, in which Fuc30 isolated from breast-fed infant faecal microbiome was found unrelated to GH29-A or GH29-B subfamilies^15^.

Clusters 1 and 13 contained α1,3/4 fucosidases active towards α1,3/4 fucosylated GlcNAc found in Lewis antigens (Table S1). The convergency ratios of clusters 2, 3 and 4 were lower than 0.30, indicating that these clusters were not isofunctional. Consistent with this, fucosidases belonging to clusters 2, 3, and 4 have been reported to have promiscuous activities for α1,2/3/4/6 fucosyl linkages (Table S1). Fucosidases in clusters 2 and 3 have been reported to release Fuc from xyloglucans^8,14,34,35^. Cluster 2 also contained the newly found exo-α-l-galactosidase BpGH29 from *Bacteroides plebeius* DSM 17135^36^. Fucosidases from clusters 3 and 47 as well as non-clustered FucWf4 from *Wenyingzhuangia fucanilytica* CZ1127^T^ have been shown to release terminal α1,3/4 Fuc from sulfated fucooligosaccharides^37,38^. Most fucosidases in cluster 4 are of animal origin. Cluster 5 contained fucosidases that specifically act on α1,3 fucosyl linkages with cFase I from *Elizabethkingia meningoseptica* FMS-007 cleaving α1,3 Fuc from the core GlcNAc position from intact glycoproteins^39^. Cluster 6 contained two characterised fucosidases, BF0810 from *Bacteroides fragilis* NCTC 9343 active on pNP-Fuc but not on natural substrates with α1,2/3/4/6 linkages^40^; and Fuc5372 isolated from breast-fed infant faecal microbiome, with preference for α1,2 fucosyl linkages found in HMOs and blood group antigens^15^. Clusters 7, 8 and 10 contained fucosidases with relatively high catalytic efficiency towards aryl-Fuc and marginal activity against α1,2/3/4 fucosyl linkages^7,10,14,40,41^. Cluster 9 contained Fuc1584 from breast-fed infant faecal microbiome which acts on α1,3/4/6 fucosyl linkages^15^. Clusters 11 and 41 contained α1,6 specific fucosidases with no activity to α1,2/3/4 fucosyl linkages^15,42^. In cluster 16, AlfB from *Lactobacillus casei* BL23 has been reported to be over 800-fold more active on α1,3 fucosylated GlcNAc than on α1,4 fucosylated GlcNAc with the non-terminal Gal in LeX abrogating its activity^42^. Cluster 26 contained site-specific core α1,6 fucosidase AlfC from *L. casei* BL23^42^. Cluster 45 contained Afc1 from *Clostridium perfringens* ATCC 13124 which showed no activity against all aryl- and natural substrates tested^43^.

Functionally characterised fucosidases displaying transfucosylation activities were found in GH29-A clusters including clusters 2, 3, 4, 7, 8, 18 and 26 and in cluster 1 belonging to GH29-B subfamily (for full information on functionally characterised fucosidases identified in SSN clusters, see Table S1).

### Microbial GH29 enzymes showed substrate specificity in line with their SSN-cluster allocation

To further explore the sequence-function relationship across different clusters, 11 GH29 sequences were selected for biochemical characterisation (Table 1). These included seven GH29-A sequences, *Ri*GH29^2A^, *La*GH29^3A^, *Rs*GH29^3A^, *Ny*GH29^4A^, *Sg*GH29^9A^, *Ba*GH29^26A^ and *Fb*GH29^26A^ spanning six clusters to explore inter-cluster differences despite predicted substrate promiscuity of these clusters, two GH29-B fucosidases from cluster 1, *Pg*GH29^1B^ and *Sm*GH29^1B^, to explore intra-cluster differences of the largest SSN cluster, one fucosidase from cluster 44, *Bs*GH29^44B^, as the first representative of this cluster, and one non-clustered fucosidase *St*GH29^nc^ to explore sequences with poor similarities. Previously characterised fucosidases were also included as controls spanning different substrate/linkage specificities.; these were two α1,3/4 fucosidases from cluster 1, E1_10125 (E1_10125^1B^ from *R. gnavus* E1^11^ and SsFuc (*Ss*Fuc^1B^) from *Streptomyces* sp. 142^44^; TfFuc1 (*Tf*Fuc1^8A^) α1,2/6 fucosidase from *Tannerella forsythia* ATCC 43037 from cluster 8^14,45^; and Afc1 (Afc1^45B^) from *C. perfringens* ATCC 13124 from cluster 45, a predicted fucosidase but with no reported activity against any of the α1,2/3/4/6 fucosylated substrates tested^43^.

**Table 1:** List of GH29 fucosidase targets characterised in this study

	Cluster	Fucosidases	Genbank No.	Uniprot No.	Source_Phylum	Source_Strain
GH29-A	2	*Ri*GH29^2A^	CBL09437.1	D4KRD5	Firmicutes	*Roseburia intestinalis* M50/1
	3	*La*GH29^3A^	WP_169795686.1	UPI0007202582	Proteobacteria	*Lysobacter antibioticus*
	3	*Rs*GH29^3A^	EMI43122.1	M5T1J4	Planctomycetes	*Rhodopirellula* sp. SWK7
	4	*Ny*GH29^4A^	OQP40061.1	A0A1V9E1W8	Bacteroidetes	*Niastella yeongjuensis*
	8	*Tf*Fuc1^8A^	AEW21393.1	G8UMQ6	Bacteroidetes	*Tannerella forsythia* (strain ATCC 43037) (Bacteroides forsythus)
	9	*Sg*GH29^9A^	ADY12241.1	F0RXW4	Spirochaetota	*Sphaerochaeta globosa* (strain ATCC BAA-1886) (*Spirochaeta* sp. (strain Buddy))
	26	*Ba*GH29^26A^	KJY49568.1	A0A0F4KU40	Actinobacteria	*Bifidobacterium asteroides*
	26	*Fb*GH29^26A^	CDD22838.1	R6XGR0	Firmicutes	*Firmicutes bacterium* CAG:345
GH29-B	1	*Pg*GH29^1B^	BAG34209.1	B2RLG4	Bacteroidetes	*Porphyromonas gingivalis* (strain ATCC 33277)
	1	*Sm*GH29^1B^	CBJ23255.1	D3HBK5	Firmicutes	*Streptococcus mitis* (strain B6)
	1	*Ss*Fuc^1B^	AAD10477.1	Q9Z4I9	Actinobacteria	*Streptomyces* sp.
	1	E1_10125^1B^	WP_243035407.1	A0A2N5PIE7	Firmicutes	*Ruminococcus gnavus* E1
	44	*Bs*GH29^44B^	KPL16698.1	A0A0S8K4Z7	Bacteroidetes	*Bacteroides* sp. SM23_62
	45	Afc1^45B^	ABG82807.1	A0A0H2YQY6	Firmicutes	*Clostridium perfringens* (strain ATCC 13124)
	Non-clustered	*St*GH29^nc^	SMQ76172.1	A0A1Y6FTG1	Proteobacteria	*Sphingopyxis terrae* subsp. ummariensis

The genes encoding the selected GH29 fucosidases were heterologously expressed in *E. coli* and the His_6_-tag recombinant proteins purified by IMAC and gel filtration (Fig. S1). *E. coli* Tuner DE3 pLacI strain was chosen as heterologous host as it does not display any endogenous β-galactosidase activity (due to the deletion of the LacZ gene) that may interfere with the enzymatic characterisation of the recombinant enzymes.

**Fig. 2:**
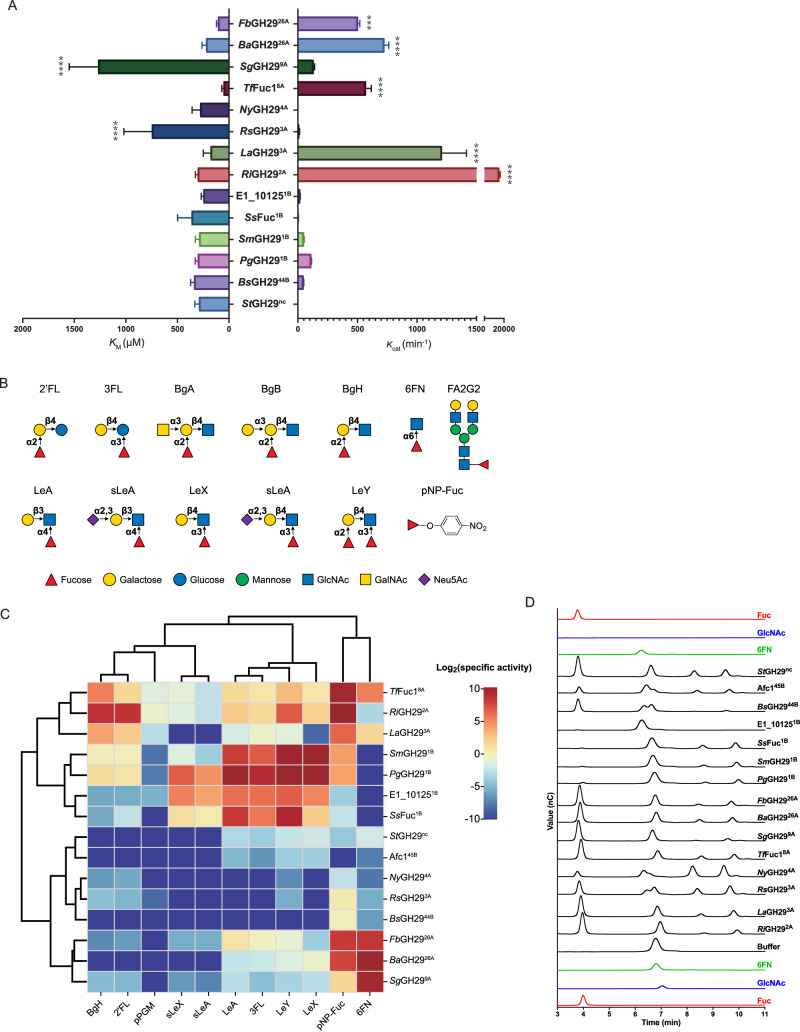
Enzymatic characterisation of GH29 fucosidases. **A** Kinetic parameters of GH29 fucosidases on CNP-Fuc. ***, p < 0.001; ****, p < 0.0001. **B** Fucosylated oligosaccharides used in this study. Monosaccharide symbols follow the Symbol Nomenclature for Glycans (SNG) system. **C** Substrate specificity of GH29 fucosidases. The data are presented in base-2 logarithm function. **D** HPAEC-PAD analysis of GH29 enzymatic reaction products with 6FN. The data were analysed with Prism. Standards were Fuc (red, 100 µM), 6FN (green, 100 µM), GlcNAc (blue, 100 µM). The black lines correspond to the enzymatic reactions with 6FN incubated with the different GH29 fucosidases tested or with buffer only. See Supplementary Fig. S2 for HPAEC-PAD analysis of GH29 enzymatic reaction products with all other substrates tested (2’FL, 3FL, BgA, BgB, BgH, LeA, sLeA, LeX, sLeX, LeY, pNP-Fuc and pPGM).

The kinetic parameters of all GH29 enzymes (*Ri*GH29^2A^, *La*GH29^3A^, *Rs*GH29^3A^, *Ny*GH29^4A^, *Tf*Fuc1^8A^, *Sg*GH29^9A^, *Ba*GH29^26A^, *Fb*GH29^26A^, *Pg*GH29^1B^, *Sm*GH29^1B^, *Ss*Fuc^1B^, E1-10125^1B^, *Bs*GH29^44B^, Afc1^45B^ and *St*GH29^nc^) were determined using CNP-Fuc (Fig. 2A, Table S2). All enzymes were found to be active towards CNP-Fuc, apart from Afc1^45B^ from cluster 45 as reported earlier^43^. The kinetic parameters of GH-29B fucosidases *Pg*GH29^1B^, *Sm*GH29^1B^, *Ss*Fuc^1B^ and E1-10125^1B^ from cluster 1, and *Bs*GH29^44B^ from cluster 44 shared *K*_m_ values around 300 µM and catalytic efficiencies between 10^-2^ and 10^-1 ^µM^-1^·min^-1^ while GH29-A fucosidases, *Ri*GH29^2A^, *La*GH29^3A^, *Rs*GH29^3A^, *Ny*GH29^4A^, *Tf*Fuc1^8A^, *Sg*GH29^9A^, *Ba*GH29^26A^ and *Fb*GH29^26A^ varied significantly in *K*_m_ values ranging from 100 to 700 µM and in catalytic efficiencies from 10^-4^ to 10^2 ^µM^-1^·min^-1^ (Fig. 2A, Table S2). *Ri*GH29^2A^ showed highest activity towards CNP-Fuc among all GH29 enzymes tested, with *k*_cat_/*K*_m_ of 58.24 µM^-1^·min^-1^, in a range similar to that of Ssα-Fuc from the neighbouring node (*k*_cat_/*K*_m_ = 10.25 µM^-1^·min^-1^)^46^.

**Fig. 3:**
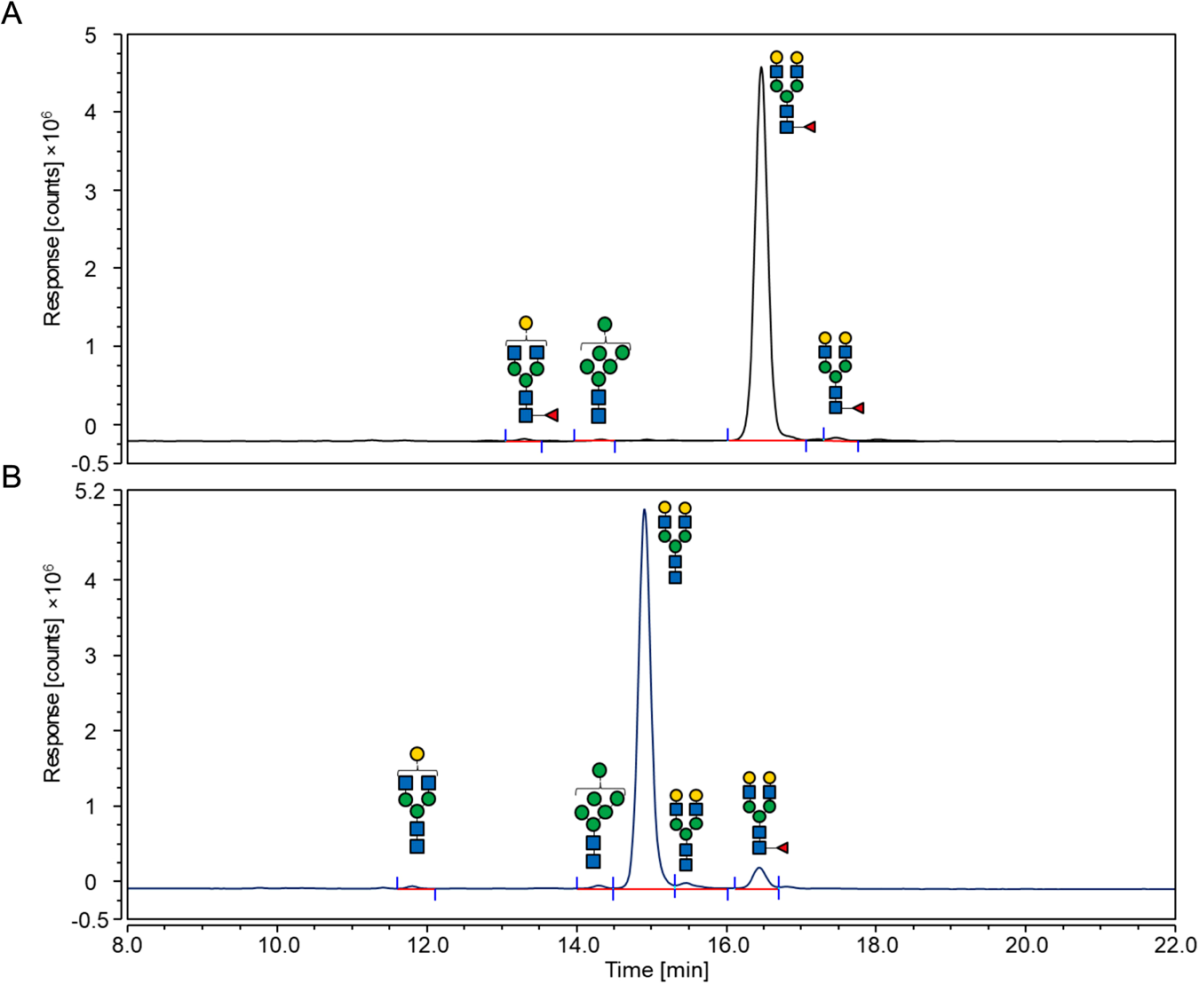
LC-FD-MS/MS analysis of *Ba*GH29^26A^ reaction with FA2G2. **A** Reaction was performed without enzyme. **B** Reaction was performed with enzyme. Glycan products are annotated next to peaks on the chromatograms.

Next, the substrate specificity of the recombinant fucosidases was tested on a range of fucosylated oligosaccharides. The specific activity was determined based on fucose release against 2′FL (Fucα1-2Galβ1-4Glc), 3FL (Galβ1-4[Fucα1-3]Glc), BgA (GalNAcα1-3[Fucα1-2]Galβ1-4GlcNAc), BgB (Galα1-3[Fucα1-2]Galβ1-4GlcNAc), BgH (Fucα1-2Galβ1-4GlcNAc), LeA (Galβ1-3[Fucα1-4]GlcNAc), sLeA (Neu5Acα2-3Galβ1-3[Fucα1-4]GlcNAc), LeX (Galβ1-4[Fucα1-3]GlcNAc), sLeX (Neu5Acα2-3Galβ1-4[Fucα1-3]GlcNAc), LeY (Fucα1-2Galβ1-4[Fucα1-3]GlcNAc), 6FN (Fucα1-6GlcNAc), pPGM and pNP-Fuc using the l-fucose assay (Fig. 2B,C, Table S3). Among all GH29-B fucosidases tested, fucosidases from cluster 1 showed highest catalytic capability for α1,3/4 linkages. The major intra-cluster variation of cluster 1 fucosidases lay in different reaction rates towards α1,2 linkages and aryl-Fuc substrates. Other GH29-B fucosidases such as *Bs*GH29^44B^ and Afc1^45B^ from clusters 44 and 45, respectively showed activity against 6FN but reduced activity against all other fucosylated linkages in natural substrates such as 2’FL, 3FL and Lewis antigens. Although all GH29-A fucosidases tested were active against pNP-Fuc, inter-cluster differences were observed. *Ri*GH29^2A^ showed preference for α1,2 linkages, *Sg*GH29^9A^, *Fb*GH29^26A^ and *Ba*GH29^26A^ for α1,6 linkages while *La*GH29^3A^, *Rs*GH29^3A^, *Ny*GH29^4A^ and *Tf*Fuc1^8A^ showed broad specificity towards α1,2/6 linkages. In addition, the enzyme activities of *Ba*GH29^26A^ and *Fb*GH29^26A^ towards pNP-Fuc were significantly higher than *Sg*GH29^9A^. *Tf*Fuc1^8A^ showed higher activity against LeX than *Ny*GH29^4A^, *La*GH29^3A^ and *Rs*GH29^3A^, whilst the latter two enzymes were more active towards pNP-Fuc than *Ny*GH29^4A^. The non-clustered *St*GH29^nc^ fucosidase showed a similar enzymatic profile towards α1,3/4/6-linked fucosylated substrates to that of Afc1^45B^ but the later showed no activity on aryl-Fuc (Fig. 2C, Tables S2 and S3). The lack of detectable activity observed for some enzyme-substrate pairs may be due to the sensitivity and risk of interference of the l-fucose assay. Therefore HPAEC-PAD analyses were conducted, confirming the release of Fuc in all active enzymes (Figs. 2D and S2). In addition, traces of Fuc were detected for enzymatic reactions not detectable by the colorimetric assay such as *Ri*GH29^2A^, *La*GH29^3A^, *Rs*GH29^3A^, *Tf*Fuc1^8A^, *Sg*GH29^9A^, *Fb*GH29^26A^, *Pg*GH29^1B^, *Sm*GH29^1B^, *Ss*Fuc^1B^, and E1_10125^1B^ against blood group A/B type II antigens (see Fig. S2) but showed the same linkage preference. LC-FD-MS/MS was then used to further investigate the activity of α1,6-specific *Ba*GH29^26A^ and *Sg*GH29^9A^ enzymes, as well as *Ny*GH29^4A^ which co-locates in SSN with eukaryotic GH29s with reported activity towards α1,3/6 core fucosylated glycoproteins^47^ on FA2G2, PLA2 or IgG glycan or glycoprotein. *Ny*GH29^4A^ but not *Sg*GH29^9A^ or *Ba*GH29^26A^ showed activity towards IgG glycan (Fig. S3A); *Ba*GH29^26A^ was tested on FA2G2, showing release of Fuc (Fig. 3) while none of the three enzymes tested showed activity on IgG glycoprotein, PLA2 *N*-glycan or glycoprotein (Fig. S3B, C, D).

**Fig. 4:**
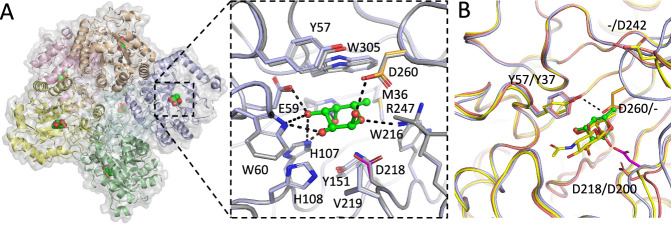
Crystal structure of *Ba*GH29^26A^. **A** Crystal structure of *Ba*GH29^26A^ in complex with Fuc. Boxout shows ligand bound WT *Ba*GH29^26A^ in light blue and unbound D218N in grey. The bound Fuc residue is shown in green. The catalytic acid base and nucleophile residues are highlighted in orange and magenta, respectively. Hydrogen bonding interactions are indicated with black dashed lines. **B** Proposed rotation of active site Tyr57 in the presence of substrate molecules suggested by alignment to AlfC, show bound to 6FN in yellow (PDB 6OHE) and bound to fucose in pink (PDB 6O1A). *Ba*GH29^26A^ is shown in light blue.

**Fig. 5:**
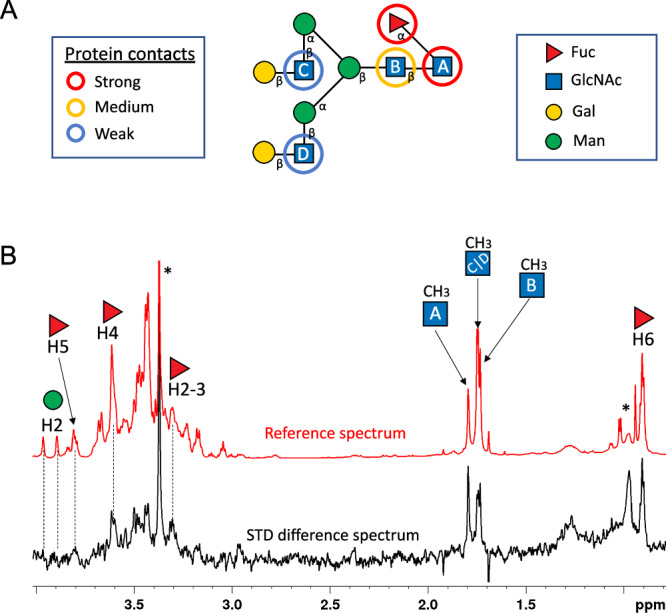
STD-NMR analysis of the interaction between *Ba*GH29^26A^ and FA2G2. **A** Binding epitope mapping of FA2G2 as bound to *Ba*GH29^26A^ from STD NMR experiments. Protein contact strength reflects relative values of saturation transfer after normalisation to the most intense one (the methyl group of GlcNAc(A)) obtained from STD initial slopes (full STD NMR build-up curves and initial slopes for each proton can be found in Fig. S5). **B** STD NMR difference (black) and reference (red) spectra of the FA2G2/*Ba*GH29^26A^ D218A sample, acquired at 2 s saturation time. Only isolated protons that were unambiguously assigned could be analysed for binding epitope determination and are labelled on the spectra (impurities are marked with *). The STD NMR analysis supports that the enzyme preferentially recognises the reducing end, with sugar rings of Fuc and GlcNAc(A) showing the strongest STD intensities.

X-ray crystallography was used to gain structural insights into *Ba*GH29^26A^ α1,6 specificity. It was only possible to grow diffracting crystals of *Ba*GH29^26A^ wild-type (WT) in the presence of 2’FL, resulting in a complex with Fuc bound in the active site while the nucleophile mutant *Ba*GH29^26A^D128N was crystallised in an apo-form (Fig. 4A, Supplementary Data 1&2). Data collection and refinement statistics are detailed in Table S4. *Ba*GH29^26A^ WT and mutant displayed a (α/β)8-fold, typical of GH29 enzymes (Fig. 4A). Asp218 was identified as the catalytic nucleophile based on its homology with other GH29 enzymes and proximity to the Fuc residue (2.9 Å from the ring oxygen of the bound sugar) (Fig. 4A). We propose Asp260 as the putative acid/base residue based on its superimposition with Asp242 of AlfC^26A^ from *Lactobacillus casei* W56 (Fig. 4B), although this catalytic residue was not experimentally validated^48^. Asp218 is flanked by the structurally conserved Tyr151 that donates a hydrogen bond to the nucleophile, as previously observed in TmαFuc^7A^ and E1_10125^1B^ fucosidases from *T. maritima* and *R. gnavus* E1, respectively^11,49^. Extensive hydrogen bonding interactions were observed between the active site and the bound sugar hydroxyl groups (Fig. 4A). The C6 methyl group sits in a hydrophobic pocket formed by Trp216 and Trp305 (Fig. 4A). Unlike E1_10125^1B^, which showed evidence of β-fucose bound^11^, the electron density of the *Ba*GH29^26A^ complex most clearly matched α-Fuc (Fig. S4A). Furthermore, attempting to model β-Fuc led to a steric clash with Asp210. High B-factors were observed in the residues surrounding the active site, indicating that there may be plasticity in the presence of larger substrate molecules (Fig. S4B). However, minimal conformation changes were observed when comparing the Fuc-bound active site in the WT enzyme to the unbound active site in the D218N catalytic mutant (Fig. 4A). Compared to fucosidases E1_10125^1B^ from *R. gnavus* E1 and Blon_2336^1B^ from *B. longum* subsp. *infantis*, the *Ba*GH29^26A^ active site was shown to be constricted (Fig. S4C, D), which may contribute to the substrate specificity of this enzyme. Tyr57 may be implicated in *Ba*GH29^26A^ α1,6 linkage specificity. This residue which hydrogen bonds with the catalytic acid/base (Fig. 4B), is structurally conserved in AlfC^26A^ (as Tyr37), which is in the same SSN cluster as *Ba*GH29^26A^ and shows specificity to α1,6-linked Fuc^48^. Tyr37, forming an aromatic subsite in AlfC^26A^, has been shown to change conformation in the presence of the α1,6-linked ligand by providing a stacking interaction with the monosaccharide linked to Fuc^48^. Such conformational change and function may be conserved in *Ba*GH29^26A^. In contrast, an equivalent residue is absent in E1_10125^1B^ and Blon_2336^1B^ from *B. longum* subspecies *infantis*, both belonging to cluster 1. Fucosidases from this cluster show a preference for α1,3/4 fucosyl linkages as compared to α1,6 Fuc (Fig. S4C, D). From closer inspection of the active site, it is expected that *Ba*GH29^26A^ Ile284 would clash with substrates presenting α1,3/4 linkages, whereas the presence of an acidic residue in this position in Blon_2336^1B^ would create a stabilising hydrogen bond to the substrate (Fig. S4D). To gain further structural insights into the ligand specificity of *Ba*GH29^26A^, saturation transfer difference nuclear magnetic resonance spectroscopy (STD NMR) studies^50^ were conducted with the nucleophile mutant, *Ba*GH29^26A^D218A in the presence of FA2G2 (Fig. 5 and Fig. S5). The D218A mutation allowed the NMR study to focus on the process of molecular recognition of the substrate, disentangling it from the subsequent enzymatical reaction. Transfer of magnetisation as saturation from the protein to the ligand was observed, in agreement with the activity of *Ba*GH29^26A^ for this substrate. Due to the large size of FA2G2 (decasaccharide), the 1D NMR spectrum showed significant chemical shift overlapping, challenging the analysis. For this reason, only isolated protons were assigned and quantitatively analysed (i.e. protons H5 and H6 of fucose, H2s of mannose and the methyl group of the four GlcNAc rings) (Fig. 5A). A full build-up curve analysis of their STD intensities showed that the enzyme intimately recognised the reducing end sugar residues constituting FA2G2 (Fig. 5B and Fig. S5) while no significant differences between reference and STD spectra were found for non-reducing end binding epitopes. The main contacts were restricted to Fuc and GlcNAc (Fig. 5A) residues, whereas only loose contacts were observed with the distant GlcN moieties (Fig. 5B).

### SSN clustering informed on transfucosylation activity of microbial GH29-A fucosidases

**Fig. 6:**
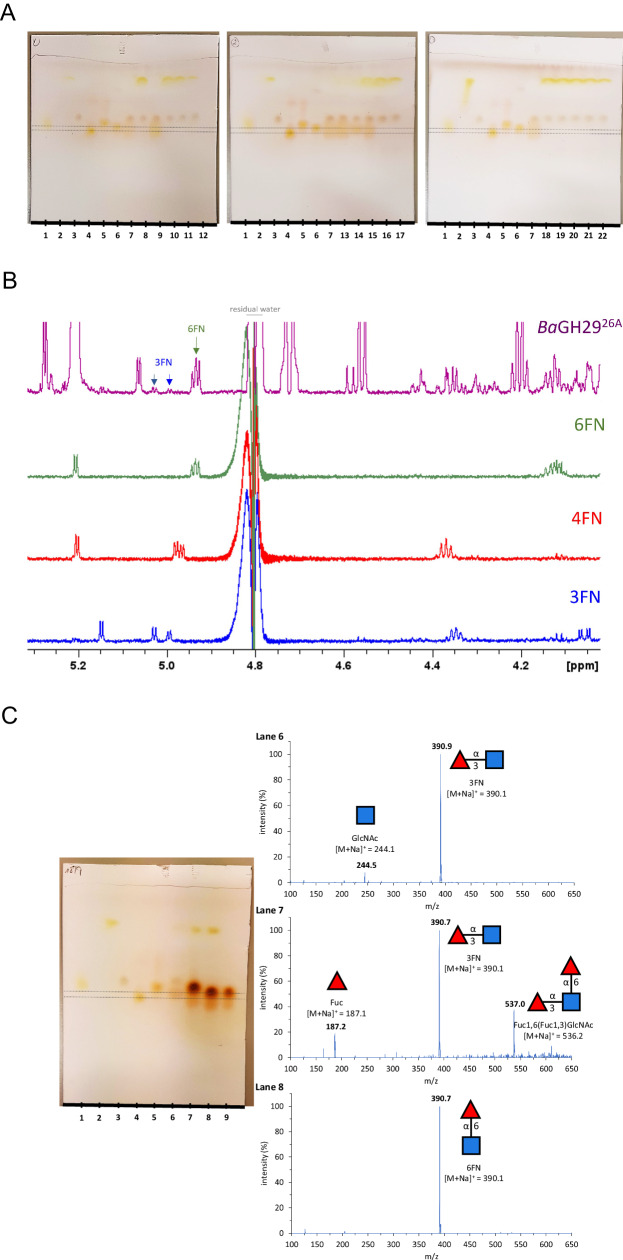
Transfucosylation activity of GH29 fucosidases. **A** TLC analysis of GH29 transfucosylation reactions with GlcNAc as acceptor and pNP-Fuc as donor; ATCC_03833^3A^ was used as control. Lanes 1 to 6 correspond to standards: Fuc (lane 1), pNP-Fuc (lane 2), GlcNAc (lane 3), 4FN (lane 4), 3FN (lane 5) and 6 is 6FN (lane 6). Lane 7 is the control reaction with ATCC_03833^3A^. Lanes 8 to 22 are the GH29 reactions with *Pg*GH29^1B^ (lane 8), *Ri*GH29^2A^ (lane 9), *Sm*GH29^1B^ (lane 10), *Ss*Fuc^1B^ (lane 11), *Tf*Fuc1^8A^ (lane 12), *La*GH29^3A^ (lane 13), *Ba*GH29^26A^ (lane 14), *Fb*GH29^26A^ (lane 15), *Rs*GH29^3A^ (lane 16), Afc1^45B^ (lane 17), *Sg*GH29^9A^ (lane 18), *Bs*GH29^44B^ (lane 19), *St*GH29^nc^ (lane 20), *Ny*GH29^4A^ (lane 21) and E1_10125^1B^ (lane 22). The upper grey dotted line corresponds to the 6FN standard and the lower grey dotted line corresponds to the 4FN substrate control. **B** 600 MHz ^1^H NMR spectra of *Ba*GH29^26A^ reaction and standards of 3FN, 4FN and 6FN. The mid field region displays distinctive signals showing the presence of 6FN and trace levels of 3FN in *Ba*GH29^26A^. **C** TLC and TLC-ESI-MS analysis of *Ba*GH29^26A^ transfucosylation reactions with 6FN as acceptors and pNP-Fuc as donor. Lanes 1 to 5 and 9 correspond to standards: Fuc (lane 1), pNP-Fuc (lane 2), GlcNAc (lane 3), 4 is 4FN (lane 4), 3FN (lane 5) and 6FN (lane 9). Lanes 6 to 8 are the *Ba*GH29^26A^ reactions with GlcNAc (lane 6), 3FN (lane 7) and 6FN (lane 8). The upper black dotted line corresponds to the 6FN standard, and the lower black dotted line corresponds to the 4FN standard. Glycan symbols follow the SNFG.

To test the transfucosylation capacity of the GH29 fucosidases characterised above, the recombinant enzymes were first assayed using GlcNAc as acceptor and pNP-Fuc as donor. The GH29 fucosidase ATCC_03833^3A^ from *R. gnavus* ATCC 29149^11^ showing 73.0% similarity to aLfuk1^3A^ from *Paenibacillus thiaminolyticus* (both in cluster 3) was used as a control as aLfuk1^3A^ was previously shown to catalyse the transfer of α-l-fucosyl moiety to different pNP-glycopyranosides with pNP-Fuc as donor^51^. The analysis of the reaction products by TLC showed the formation of transfucosylation products by ATCC_03833^3A^, *Ri*GH29^2A^, *La*GH29^3A^, *Ba*GH29^26A^ and *Fb*GH29^26A^ characterised by the presence of spots with similar retention factors (from 0.52 to 0.57) that may correspond to potential 4FN, 3FN, and 6FN transfucosylation products (Fig. 6A and Fig. S6A, B). These results are in agreement with the SSN analysis showing that GH29 enzymes with reported transglycosylation activity with GlcNAc as acceptor are distributed in clusters 2, 3, 8, and 26 belonging to GH29-A subfamily with the exception of *Rs*GH29^3A^ where no transfucosylation product was observed likely due to its negligible hydrolytic activity (Fig. 6A). None of the GH29-B fucosidases tested showed transfucosylation activity using this acceptor-donor pair (Fig. 6A). Since the R_*f*_ values for 3FN, 4FN and 6FN (0.57, 0.52 and 0.55, respectively) on TLC could not discriminate between the products formed, NMR was used to gain further insights into the linkages of the transfucosylation products. Transfucosylation reactions with α1,6 fucosidases *Ba*GH29^26A^ and *Fb*GH29^26A^ resulted in the synthesis of 6FN, in line with their substrate preferences (Figs. 6B and S6C). Both ATCC_03833^3A^ and *La*GH29^3A^ transglycosylation reactions led to the production of 6FN and 3FN (Fig. S6C). The NMR analysis also showed that 3FN was the main product generated by *Ri*GH29^2A^ although traces of 4FN were detected (Fig. S6C).

TLC-ESI-MS was carried out to further investigate the transfucosylation capacity of *Ba*GH29^26A^ (Fig. 6C). The product of *Ba*GH29^26A^ enzymatic reaction with GlcNAc was confirmed to be a fucosylated compound (Fuc1,xGlcNAc, found *m/*z 390.1 for [M+Na]^+^, calcd for C_14_H_25_NO_10_Na 390.1) (Fig. 6C). The ATCC_03833^3A^ reaction with GlcNAc produced a fucosylated product (Fuc1,xGlcNAc, found m/z 390.8 for [M+Na]+, calcd for C14H25NO10Na 390.9) (Fig. S6A). Further transfucosylation reactions were performed using 3FN or 6FN as acceptors. Both *Ba*GH29^26A^ and ATCC_03833^3A^ produced bifucosylated products with 3FN but not with 6FN (Figs. 6C and S6A). *Ba*GH29^26A^ product of the reaction with 3FN was confirmed to be a product of fucosylation (Fuc1,x[Fuc1,3]GlcNAc, found *m/*z 537.0 for [M+Na]^+^, calcd for C_20_H_35_NO_14_Na 536.2) (Fig. 6C), the same as ATCC_03833^3A^ (Fig. S6A). For both enzymatic reactions with 6FN, the only peak produced corresponded to the acceptor (*Ba*GH29^26A^: 6FN, found *m/*z 390.7 for [M+Na]^+^, calcd for C_14_H_25_NO_10_Na 390.1; ATCC_03833^3A^: 6FN, found *m/*z 390.7 for [M+Na]^+^, calcd for C_14_H_25_NO_10_Na 390.1) (Figs. 6C and S6A). From this analysis, it is expected that the transglycosylation product of *Ba*GH29^26A^ or ATCC_03833^3A^ using 3FN as acceptors is Fuc1,6[Fuc1,3]GlcNAc, in agreement with their α1,6 substrate specificity.

### SSN clustering prediction via pLMs

SSN clustering ID can be regarded as a simplified function label of enzyme properties such as substrate specificity and transfucosylation capability. Therefore, predicting SSN clustering of unexplored GH29 sequences can shed light into their enzymatic properties. Here, a semi-supervised deep learning method was performed to train a pLM, termed GH29BERT, for clustering novel GH29 sequences into the top 45 clusters of the existing SSN (accounting for 96.33% of all sequences within SSN) (Fig. S7). The pre-training dataset comprised 34,258 non-redundant GH29 sequences (i.e., unlabelled data) extracted from CAZy and Interpro databases. The obtained self-supervised pre-trained model containing 5 repeated blocks of Transformer encoders incorporated 20 million (M) parameters carrying features extracted directly from protein sequences (Table S5). The supervised dataset, with a random 80%-20% split for training and testing, respectively, included 2,796 labelled sequences extracted from the top 45 SSN clusters excluding 14 sequences of clustered fucosidases characterised in this work as listed in Table 1. The classifier model obtained by supervised task-training can interpret the outputs of the pre-training model to SSN clustering IDs via two attention layers, three densely connected layers, and one softmax classification layer consisting of ~0.6 M parameters tailored for fine-tuning. We further applied this fine-tuning technique to two state-of-the-art pLMs, ESM-2^28^ and ProtT5-XL-U50 model (abbreviated here as ProtT5)^29^, for comparing their GH29 SSN ID cluster prediction performance. These pLMs are in larger scale than GH29BERT with model parameters ranging from 8 M to 650 M for ESM-2, and 1.2 billion (B) for ProtT5 (Table S5).

As shown in Table S5, GH29BERT accurately allocated most GH29 sequences of the test dataset to their corresponding cluster IDs, achieving 98.21% accuracy. ProtT5 demonstrated best performance, achieving 99.60% of accuracy and ESM-2 models attained up to 99.28% of accuracy, indicating exceptional protein sequence modelling power of large-scale pLMs pre-trained on databases in million scale. The Pearson correlation coefficient between prediction accuracies of pLMs and the ECE perplexities is - 0.97, indicating that ECE can accurately measure the confidence of pLM prediction. The ECE values were close to 1 for all pLMs, indicating that the SSN clustering allocation by pLMs was of high confidence. Such accuracy and ECE perplexity were highly superior to that obtained using baseline models without pretraining steps, i.e., non-pretrained GH29BERT and one-hot model described in Table S5), highlighting the importance of context-aware representation learning approach for protein sequence representation. The allocation of the 14 target sequences listed in Table 1 was then determined using three pLMs, GH29BERT, ESM-2, and ProtT5, to assess the prediction performance on sequences out of the supervised dataset. The analysis showed that the three pLMs accurately allocated these sequences into their corresponding SSN clusters.

**Fig. 7:**
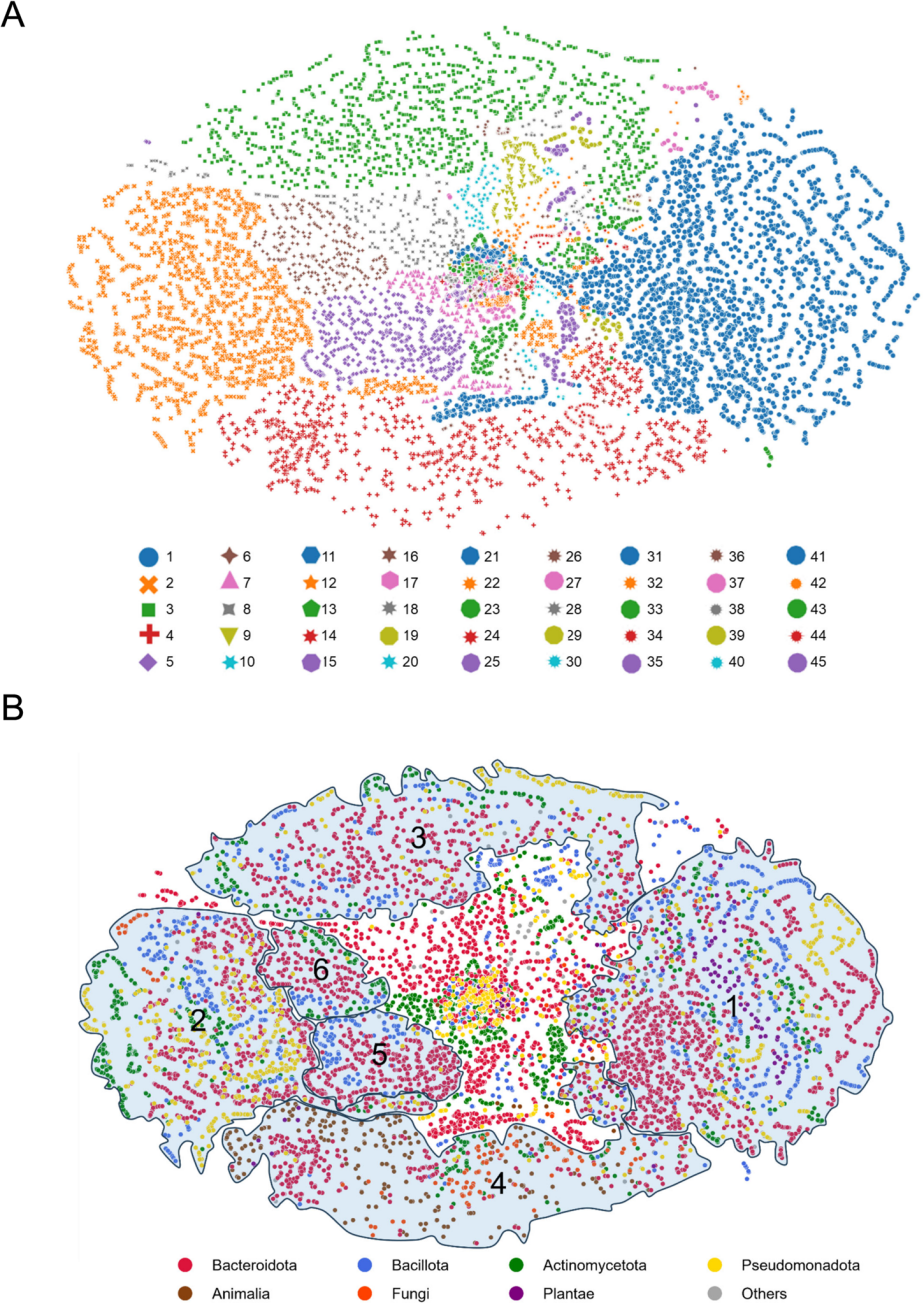
Sequence representations of GH29 family. ProtT5 was employed to map each of 34,258 non-redundant GH29 sequences into a 1024-dimension representation followed by SSN cluster ID allocation. These representations were further projected onto 2-dimension space (x- and y-axes) using UMAP for visualisation and colour-coded by predicted SSN ID clustering and taxonomy as shown in (**A**) and (**B**), respectively. Each dot on the map represents a sequence. The original (**A**) is supplied in supplementary data 6 for maximum resolution. The number in each region in panel B) corresponds to SSN clustering ID.

Due to its highest accuracy of clustering assignment (described above), ProtT5 was next used to explore the ID clustering distribution of 34,258 non-redundant GH29s, although it should be noted that GH29BERT is most advantageous in terms of computational cost. The taxonomy information of these sequences is summarised in supplementary data 3, the majority of these GH29 sequences (82.2%) belonged to bacteria while 15.8% derived from eukaryotes including animals (10.2%), fungi (3.1%), plants (2.2%) and protisa (0.3%). The top 10 abundant bacterial phyla were Bacteroidota (34.0%), Bacillota (13.3%), Actinomycetota (11.3%), Pseudomonadota (6.4%), Planctomycetota (4.2%), Verrucomicrobiota (2.7%), Acidobacteriota (2.4%), Chloroflexota (1.3%), Lentisphaerota (1.2%) and Armatimonadota (0.9%). The information extracted by ProtT5 was then visualised after dimension reduction using UMAP^52^ (Fig. 7). The topology of the sequence representation by ProtT5 divided the sequence space of GH29 into well-separated clusters, indicating its good performance in capturing inter-cluster differences (Fig. 7A, Supplementary Data 4). As summarised in supplementary data 3, GH29 sequences from the top 4 clusters accounted for 67.5% of the total sequences, where cluster 1 (21.0%), cluster 2 (14.0%), cluster 3 (16.4%), cluster 4 (16.1%) represented super-clusters in terms of size (Fig. 7, supplementary data 3). In the future, it might be necessary to subdivide clusters 2, 3 and 4 to enhance isofunctionality within subclusters. Most sequences in clusters 1, 2 and 3 were derived from bacteria while cluster 4 was composed of 22.1% bacterial fucosidases and 76.3% eukaryotic fucosidases (Fig. 7B, supplementary data 3). Most clusters contained relatively evenly distributed bacterial phyla while clusters 10 and 11 were dominated by Bacteroidota, cluster 26 was mostly from Bacillota, and clusters 15, 17 and 23 were mostly from Actinomycetota (Fig. 7B, supplementary data 3).

## Discussion

Reflecting the high diversity of fucosylated structures in nature, microbes produce a range of α-l-fucosidases with different linkages specificity^4^. Here, we combined SSN analysis and deep learning approaches, underpinned by biochemical characterisation of selected enzymes, to fully explore the structure-function space of GH29 α-l-fucosidases, expanding the GH29 enzyme toolbox. GH29 enzymes are divided into GH29-A and GH29-B, displaying broad and narrow substrate specificity, respectively. The acid/base residues of GH29-B enzymes are conserved and assignable from primary sequence alignments^53^ in contrast to GH29-A where catalytic residues are less conserved^35,48–51,54,55^. Here, we first showed using SSN that GH29-A sequences were spread within 18 clusters as compared to 3 clusters for GH29-B sequences, in line with the high variability characteristic of GH29-A enzymes. Notably, cluster 1 with 560 fucosidase sequences belonging to GH29-B, and clusters 2 and 3 belonging to GH29-A accounted for over half of the GH29 sequences.

The recombinant GH29 enzymes characterised in this work spanning different SSN clusters showed substrate specificities in line with functionally characterised GH29 fucosidases from these clusters. For example, *Pg*GH29^1B^, *Sm*GH29^1B^ and *Ss*Fuc^1B^ belonging to cluster 1 showed Fuc1,3/4GlcNAc and Fuc1,3Glc linkage preferences albeit with different catalytic efficiency. None of the fucosidases from cluster 1 GH29-B were active on 6FN. *Ri*GH29^2A^ belonging to cluster 2 showed preference for α1,2 Gal linkages and displayed transfucosylation activity, as also reported for FgFCO1 from *Fusarium graminearum* PH-1 belonging to the same cluster^14,35,56,57^. *La*GH29^3A^ and *Rs*GH29^3A^ belonging to cluster 3 showed highest activity towards pNP-Fuc but no significant activity was found towards α1,2/3/4/6 fucosylated substrates, as also reported for Fp251, Fp239 and Fp231 from *Paraglaciecola sp*^58^, Alf1_Wf from *W. fucaniytia* CZ1127^T38^ and ATCC_03833^3A^ from *R. gnavus* ATCC 29149^11^ found in the same cluster. *Ny*GH29^4A^ belonging to cluster 4 showed preference for Fucα1,2 Gal, consistent with Fucosidase O from *Omnitrophica* bacterium OLB16^59^, HsFucA1^9^ and *Hs*FucA2^60^ from *Homo sapiens* found in the same cluster. *Sg*GH29^9A^ belonging to cluster 9 was highly active on 6FN with marginal activity towards pNP-Fuc, similar to Fuc1584 isolated from breast-fed infant faecal microbiome in the same cluster^15^. *Ba*GH29^26A^ and *Fb*GH29^26A^ belonging to cluster 26 were active on both 6FN and pNP-Fuc, which might be associated with their transfucosylation activity, as shown with AlfC from *L. casei*^12,13^. *Bs*GH29^44B^ from cluster 44 (which does not contain any functionally characterised enzymes) and non-clustered *St*GH29^nc^, showed low activity against all substrates tested. Collectively, these data confirmed that SSN is a reliable approach to predict the substrate specificity of GH29 enzymes belonging to characterised clusters. Recent bioinformatic analyses based on Conserved Unique Peptide Patterns (CUPP) were applied to predict the substrate specificity and transglycosylation capacity of GH29 enzymes^16,21^. Among the novel GH29 α-L fucosidases characterised as part of this latest work, BT3665^2A^ and BT3956^11A^ from *B. thetaiotaomicron* VPI-5482 showed substrate preference for 2’FL and 6FN, respectively^15,16^, which is in line with their presence in SSN clusters 2 and 11, respectively, encompassing enzymes of similar substrate specificity. Cluster 11 also included Fuc30^11A^ from breast-fed infant faecal microbiome with the same substrate specificity^15^. *Wf*Fuc^20A^ from *Wenyingzhuangia fucanilytica* from cluster 20 showed activity towards 3FL, *Ao*Fuc^15A^ from *Amycolatopsis orientalis* from cluster 15 showed no activity to natural substrates, and *Ac*Fuc^19A^ from *Acidobacterium capsulatum* from cluster 19 was active on 3FL and 3FN^16^, filling the knowledge gaps for these clusters. CloFuc from *Clostridium porci* and *Sw*Fuc from *Sphingobacterium wenxinia* showed α1,3/4 and α1,3 fucosidase activity^16^, respectively, in accordance with their predicted cluster IDs via ProtT5, cluster 1 and 3, respectively.

The GH29-A enzymes characterised in this work showed wider kinetic parameter range towards pNP-Fuc and CNP-Fuc substrates than GH29-B enzymes. Five of the six GH29-B enzymes tested were active against pNP-Fuc and CNP-Fuc substrates, which is challenging the dogma that GH29-B enzymes are not active on these substrates, as also supported by previously characterised GH29-B fucosidases from cluster 1 i.e. BT1625 from *B. thetaiotaomicron* VPI-5482^53^, Eo0918 from *Emticicia oligotrophica* DSM 17448^61^, and Blon_2336 from *B. longum* subsp. *infantis* ATCC 15697^7^. Collectively, our results showed that GH29-B enzymes have a preference for Lewis antigen epitopes while the linkage preference of GH29-A enzymes varies between clusters, but fucosidases from both subfamilies can display strict linkage preferences.

As α1,6-linked Fuc in core position is a target for biotechnological and biomedical applications including diagnostics^47,62^, we further investigated the structural basis for *Ba*GH29^26A^ substrate specificity. *Ba*GH29^26A^ is derived from *Bifidobacterium asteroides*. Bifidobacteria are common gut commensal bacteria specialised in HMO degradation and metabolism^63^. Although many α-l-fucosidases from Bifidobacteria including *B. bifidum* and *Bifidobacterium longum* subsp. *infantis*^7,64,65^ have been identified by bioinformatics analysis, as belonging to GH29, GH95, and GH151 families, few have been functionally characterised. In the GH29 family, bifidobacterial α-l-fucosidases have been divided into GH29-BifA fucosidases (only found in *B. bifidum* strains), GH29-BifB fucosidases, GH29-BifC fucosidases, and GH29-BifD fucosidases based on their domain conservation and phylogeny^66^. *Bb*AfcB belonging to cluster 1, the only characterised representative GH29 fucosidase of GH29-BifA, has been shown to be active on 3FL, Lewis group antigens (A, B, X, and Y), and lacto-N-fucopentaose II and III but not on glycoconjugates containing α1,2-fucosyl residue or on synthetic pNP-Fuc^65^. GH29-BifB shares the same catalytic domain as GH29-BifA but lacks additional protein domains (i.e. F5/8 type C and FIVAR domains)^66^. In line with this, the only characterised bifidobacterial GH29-BifB fucosidase, belonging to cluster 1, Blon_2336 from *B. longum* subsp. *infantis* ATCC 15697, revealed similar substrate preferences to *Bb*AfcB (GH29-BifA) including towards 3FL and LeX epitopes^7,17^. These GH29-BifB fucosidases appear to be distributed across bifidobacterial strains of different species (unlike GH29-BifA fucosidases) and frequently, strains that exhibit GH29-BifB fucosidases also produce GH29-BifC fucosidases. GH29-BifC fucosidases, Blon_0426 and Blon_0248 from *B. longum* subsp. *infantis* ATCC 15697 belonging to cluster 7, can catalyse the hydrolysis of core α1,6-fucose on the *N*-glycan of glycoprotein and Fuc1-6GlcNAc-IgG^67^. Among the *B. asteroides* strains which have been genome-sequenced to date, fucosidase-encoding genes are restricted to GH29 family, with one GH29-encoding gene in the DSM 20089 and PRL2011 strains and 3 in the ESL0447 strain (www.cazy.org). Based on phylogeny analysis, these would fall into GH29-BifD, the function of which was previously unreported. Here, we showed that *Ba*GH29^26A^ from *B. asteroides*, in cluster 26, exhibited α1,6 substrate specificity and could hydrolyse FA2G2 *N*-glycan through recognition of 6FN epitopes, as shown by STD NMR but could not release Fuc from *N*-glycan unlike GH29-BifC, indicating that GH29-BifC and GH29-BifD differ in their capacity to accommodate the reducing end of *N*-glycan. *Ba*GH29^26A^ crystal structure displayed the conserved GH29 catalytic machinery. The α1,6 substrate specificity of *Ba*GH29^26A^ may result from the presence of the Tyr57-containing loop which is missing in cluster 1 fucosidases, as previously suggested for AlfC from *L. casei* BL23^48^ found in the same SSN cluster. Additionally, compared to Blon_2336 (GH29-BifB) for which a 3D complex is available, it is expected that the constricted active site in *Ba*GH29^26A^ through the intrusion of Ile284 will obstruct access to α1,3 and α1,4 linked fucosylated substrates. Structural features within the GH29 substrate binding region may also contribute to intra-cluster differences in GH29 fucosidase activities towards non-preferred substrates, as shown for example for *Ba*GH29^26A^ and *Fb*GH29^26A^ against Lewis antigens or *La*GH29^3A^ and *Rs*GH29^3A^ against 2’FL and BgH. This was illustrated by the crystal structure of *Sp*GH29 in complex with LeX (6ORF), where the trisaccharide was bound to β5 to β7 strands^68^ which are regions of poor conservation across GH29 fucosidases due the lack of α5 affecting β6 to β7 strand within the TIM barrel. These features may therefore explain the subtle discrepancies in substrate specificities within enzymes of overall high sequence identity.

GH29 α-l-fucosidases display a retaining double-displacement mechanism with retention of anomeric configuration^50^, allowing the catalysis of transglycosylation reactions leading to the synthesis of oligosaccharides, such as fucosylated HMOs. Previously characterised α-l-fucosidases AlfB and AlfC from *Lacticaseibacillus* c*asei* W56 have been shown to synthesise 3FN, 6FN, the glycoamino acid 6FN (Fuc-α-1,6-*N*-GlcNAc-Asn), and several 6′-fucosyl-glycans^12,13^. Fucosyl-N-GlcNAc disaccharides have also been produced using *Bacteroides fragilis* α-l-fucosidase^40^. The HMOs, 2′FL, 3FL, and lacto-N-fucopentaose II, have been synthetised in low amounts using α-l-fucosidases from *T. maritima*, *C. perfringens*, and a soil-derived metagenome library^14,69^. Here, we showed that, using pNP-Fuc as donor and GlcNAc as acceptor, *Ba*GH29^26A^ and *Fb*GH29^26A^ from cluster 26 produced 6FN as sole transfucosylation product, *Ri*GH29^2A^ from cluster 2 produced 3FN and 4FN but not 6FN while *La*GH29^3A^ and ATCC_03833^3A^ from cluster 3 produced 6FN and 3FN but not 4FN. These results suggest that the substrate specificity observed during catalysis was retained during transfucosylation. Although *Ri*GH29^2A^ and *La*GH29^3A^ were not tested against 3FN and 4FN, *Ri*GH29^2A^ showed the least activity against 6FN among all substrates tested while *La*GH29^3A^ may share specificity with its neighbouring node from cluster 3, *Am*GH29A from *Akkermansia muciniphila* ATCC BAA-835, which showed activity towards 3FN but not 4FN^70^, but this would need to be experimentally validated. Bi-fucosylated-GlcNAc products were produced by *Ba*GH29^26A^ with 3FN as donor. These newly characterised GH29 α-l-fucosidases might therefore be exploited as biotechnological tools in the synthesis of oligosaccharides that may be used as prebiotics for promoting the growth of Bifidobacteria in the gut. In addition, our work highlighted the suitability of SSN as a tool to predict transfucosylation capacity for sequences falling into clusters containing functionally characterised transglycosylating α-l-fucosidases. This was further supported by findings from the recent CUPP study^16^ where newly characterised GH29 α-l-fucosidases with transglycosylation activity are distributed in SSN clusters predicted to include such activity i.e. cluster 2 (BT3665, FgFCO1, NixE from *Xanthomonas campestris* pv. *campestris* str. ATCC 33913), cluster 3 (Mfuc5 from soil metagenome), cluster 8 (TfFuc1), cluster 1 (*Bb*AfcB from *B. bifidum* ATCC 1254 and *Cp*Afc2 from *Clostridium perfringens* ATCC 13124). Other recently characterised enzymes such as *Ao*Fuc^15A^, *Ac*Fuc^19A^, showed transfucosylation activity, expanding the number of clusters with transfucosylation capacity to 10 (cluster 1, 2, 3, 4, 7, 8, 15, 18, 19 and 26)^16^.

Together, this work supported the use of SSN as a platform to further explore the sequence-function of non-characterised GH29 fucosidases using pLM models to achieve an end-to-end network, i.e. from raw sequence to cluster assignment. pLMs have recently been applied to perform protein property prediction tasks such as tertiary structure or remote sequence homology^28,31^, where the highest accuracy was reported to be 91% for binary classification of membrane versus non-membrane proteins^29^. Here, we trained a GH29BERT model using a semi-supervised approach and compared its performance to two state-of-the-art large-scale pLMs, ESM-2^28^ and ProtT5^29^. The performances of these models on GH29 SSN cluster allocation prediction showed 98.21% accuracy for GH29BERT, while ESM-2 and ProtT5 attained up to 99.28 and 99.64% accuracy, respectively. This improved performance compared to previous relevant pLM applications is likely to be due to the accurately-labelled GH29 inputs generated by the SSN analysis, enhancing model performance during task-training. It is of note that the GH29BERT model required significantly fewer resources in terms of hardware and time course for both training and testing, using only 20 M parameters for its pre-training phase, compared to 650 M and 1.2 B parameters for ESM-2 and ProtT5, respectively. Together, these results indicate that the combination of SSN and pLM is an effective approach to explore the sequence-function of protein family. In this context, SSN was preferable to e.g. CUPP, due to its relaxed stringency leading to larger sample size of each cluster in order to be compatible with machine-learning approaches, for superior accuracy. By applying ProtT5 to predict clustering information for all GH29s accessible through CAZy and InterPro databases, it was found that 67.5% of the GH29 sequence-space was mostly divided into 4 clusters, which is consistent with the current SSN analysis. Cluster 1 accounted for 21.0% of the total GH29 sequences and were mostly derived from Bacteroidota, Bacillota, Actinomycetota and Eukaryota, reflecting the wide distribution of α1,3/4 fucosidases.

The continuing expansion of microbial GH families within the CAZy database, through metagenomic sequencing, with many uncharacterised or “hypothetical” proteins is an opportunity to identify novel enzymes with biotechnological applications. This work demonstrated the suitability of SSN and machine learning tools to harness the wealth of sequencing data and help predict novel fucosidases and transfucosylation activities in prokaryotes. It is expected that such combined computational approach will be applied in the future to other GH families.

## Materials and methods

### Materials

All chemicals were obtained from Sigma (St Louis, MO, USA) unless otherwise stated. 2’-fucosyllactose (2’FL) and 3-fucosyllactose (3FL) were obtained from Glycom/DSM (Esbjerg, Denmark). Blood group A type II (BgA), Blood group B type II (BgB), Blood group H type II (BgH) and LewisY (LeY) were obtained from Elicityl (Crolles, France). Lewis A trisaccharide (LeA), 3′-sialyl Lewis A (sLeA), Lewis X trisaccharide (LeX), 3’-sialyl Lewis X (sLeX), 2-acetamido-2-deoxy-6-O-(α-l-fucopyranosyl)-d-glucopyranose (6FN), 2-acetamido-2-deoxy-4-O-(α-l-fucopyranosyl)-d-glucopyranose (4FN), 2-acetamido-2-deoxy-3-O-(α-l-fucopyranosyl)-d-glucopyranose (3FN), 4-nitrophenyl α-l-fucopyranoside (pNP-Fuc), 2-Chloro-4-nitrophenyl-αl-fucopyranoside (CNP-Fuc), 2-Chloro-4-nitrophenol (CNP) and N-acetyllactosamine (LacNAc) were obtained from Biosynth Ltd (Compton, UK). FA2G2 *N*-glycan was from Ludger (Oxford, UK). IgG was purified from human serum using the protein A IgG purification kit from Thermofisher (Carlsbad, US). Purified porcine gastric mucin (pPGM) was obtained as previously described^71^. PNGase B035DRAFT_03341^72^ was a kind gift from Dr Lucy Crouch (Newcastle University). Phospholipase A2 (PLA2) from honeybee venom (*Apis mellifera*) was purchased from Sigma (St Louis, MO, USA). Recombinant fucosidases E1_10125^1B^ from *R. gnavus* E1 and ATCC_03833^3A^ from *R. gnavus* ATCC 29149 were produced in-house as previously reported^11^.

### Bioinformatics analyses

For sequence similarity networks (SSN) analysis, the sequences encoding GH29 fucosidases were extracted from CAZy database (www.cazy.org). A total of 9,505 GH29 sequences from the CAZy database (last update 2022-10-18) were winnowed down to 2,971 sequences following a sequence identity cut-off at 0.8 via CD-HIT suite^73^. The amino acid sequences were then used to generate SSN using the Enzyme Function Initiative-Enzyme Similarity Tool (EFI-EST) with an alignment score threshold of 96 (40% sequence identity)^33,74^. The SSN was visualised using Cytoscape 3.9.1. The GH29 sequences from each cluster are provided in Supplementary Data 5.

A semi-supervised training method, termed GH29BERT, was applied to implement the unsupervised protein sequence representation learning and supervised classification for GH29 fucosidases. This pLM training process is composed of two phases (as illustrated in Fig. S7), pre-training, and classification task-training, respectively. The pre-training utilised the BERT-based (Bidirectional Encoder Representations from Transformers) language model^75^ to extract features from 34,258 non-redundant GH29 sequences derived from the CAZy database (last update: 2023-10-10) and InterPro database (downloaded on 2023-11-02). A random 95%-5% data split was adopted for model pre-training and training-process validation. The original BERT model has 12 repeated blocks of Transformer encoders^76^, here we tuned this hyperparameter to 5 for best performance. Notably, the pre-training model, including a Masked Language Modelling (MLM) prediction head, enabled hiding a certain percentage of input tokens and training the model to predict them in a self-supervised approach. It implements both the next-token prediction and the previous-token prediction, facilitating bidirectional context understanding, which is critical for protein sequence modelling^29^. The classification task-training model, composed of two attention layers, three dense connected layers and one softmax classification head, was performed on 2,796 labelled sequences (see Supplementary Data 6) derived from the top 45 clusters of the SSN excluding 14 GH29 sequences which were further used for validation (see list of enzymes in Table 1). We randomly selected 80% of the labelled data for training and 20% for testing. Pre-training and task-training were executed on two NVIDIA A100 40GB GPU for one week and 2 h, respectively. In addition to evaluating the accuracy of cluster predictions, we incorporated Exponential Cross-Entropy (ECE) to assess the uncertainty of each pLM while processing input sequences. For the classification task in this study, ECE was calculated using ${e}^{1/n{\sum }_{i}^{n}{CE}({s}_{i},{y}_{i})}$, where $n$ is number of protein sequences tested, ${s}_{i}$ and ${y}_{i}$ denote the sequences and their corresponding cluster labels, respectively. ECE is also known as one kind of perplexity, which in our context ranges from 1, indicating deterministic predictions, to 45, equivalent to a completely random selection from the 45 clusters. In addition to adopting the semi-supervised method through training the pLM on GH29 fucosidase sequences from scratch, i.e., with randomly initialised model parameters, we also included two state-of-the-art pLMs, ESM-2^28^ and ProtT5 model^29^, for validating their efficacy on GH29 sequence cluster prediction. These pLMs are in larger scale than GH29BERT, in terms of training data, number of parameters, and training time, and were pre-trained on the entire known proteins (see detailed configuration comparison in Table S5). We loaded and froze their official pre-trained parameters, then used labelled GH29 sequences to fine-tune the task-training model, which had the identical structure as for GH29BERT. For comparison, we established two baselines using a non-pretrained GH29BERT and a one-hot encoding approach, respectively. Non-pretrained GH29BERT was trained directly with labelled GH29 sequences, while the one-hot method trained the same task-training model using a one-hot encoding of the protein sequence. Dimension reduction was performed using Uniform Manifold Approximation and Projection (UMAP)^52^. The GH29BERT model is accessible through a friendly user-interface: https://huggingface.co/spaces/Oiliver/GH29BERT. This web tool assigns a corresponding cluster ID to any sequence uploaded. The configuration of running environment, including all dependencies and used packages, as well as the Python version are detailed in supplementary data 7. The source code and instruction for running environment preparation are available on GitHub: https://github.com/ke-xing/GH29BERT.

### Cloning, expression and purification of fucosidases

The GH29-encoding genes were synthesised exempt of the signal peptide sequence and cloned into pET28a with N terminal His_6_-tag by Prozomix (Haltwhistle, UK). *Ba*GH29^26A^D218A and *Ba*GH29^26A^D218N mutants were synthesised by NZYTech (Lisboa, Portugal). *Escherichia coli* TunerDE3 pLacI cells were transformed with the recombinant plasmids according to manufacturer’s instructions. Expression was carried out in 1 L LB media growing cells at 37°C until OD_600_ reached 0.3 to 0.6 and then induced at 16°C for 20-22 h. The cells were harvested by centrifugation at 4,000 g for 35 min. The His-tagged proteins were purified by immobilised metal affinity chromatography (IMAC) and further purified by gel filtration on an ÄKTApure (Cytiva, Little Chalfont, UK). Protein purification was assessed by standard SDS–polyacrylamide gel electrophoresis using the NuPAGE Novex 4–12% Bis-Tris gels (Life Technologies, Paisley, UK). Protein concentration was measured with a NanoDrop (Thermo Scientific, Wilmington, USA) and using the extinction coefficient calculated by Protparam^77^ from the peptide sequence.

### Enzymatic activity assays

For kinetics, all enzymes were incubated with CNP-Fuc in 50 mM citrate buffer at pH 6 and 37°C. The amount of enzyme was determined to fulfil free-ligand approximation, i.e. the enzyme concentration was linear with product formation. The reaction duration was optimised to measure the reaction rates under initial conditions. A standard curve was made with the reaction product CNP and Fuc in 1:1 ratio from 0 to 0.3 mM to better mimic the reaction products. The release of CNP was monitored using a microplate reader (FLUOstar Omega, BMG LABTECH, Ortenberg, Germany) by monitoring absorbance at 405 nm every 2 min for 40 min in 3 technical replicates. The kinetic parameters were calculated based on the Michaelis-Menten equation using a non-linear regression analysis programme, and one-way ANOVA was performed compared to E1_10125^1B^ (Prism 5, GraphPad, San Diego, USA).

The enzymatic activity of recombinant fucosidases was determined on 2’FL, 3FL, BgA, BgB, BgH, LeA, sLeA, LeX, sLeX, LeY, 6FN, pNP-Fuc and pPGM using 10 μM enzyme, 0.5 mM substrate or 1 mg/mL for pPGM in 50 mM citrate buffer pH 6 and 1 mg/mL bovine serum albumin (BSA). The reactions were incubated at 37°C and stopped by boiling at 95°C for 10 min. The release of Fuc was quantified with the l-fucose assay kit from Megazyme (Wicklow, Ireland) using a microplate reader (FLUOstar Omega, BMG LABTECH, Ortenberg, Germany) by monitoring absorbance at 340 nm every 2 min. To determine the specific activity, the enzymatic reactions were optimised by adjusting enzyme concentration and incubation time (Table S6) to obtain between 6%-25% of substrate hydrolysis which is within detection limit and corresponds to linear range. Specific activity was calculated from 4 technical replicates. One unit of activity was defined as the amount of enzyme needed to release 1 μmol Fuc per min under the conditions described above. Enzymatic reactions were carried out as above but with 0.1 mM substrate and were incubated for 24 h, and the released Fuc were confirmed by HPAEC-PAD using a Dionex ICS 5000 system (Thermo Scientific, Hemel Hempstead, UK). The sugars were separated on a CarboPac PA1 analytical column protected with a CarboPac PA1 guard column using the following gradient conditions at 1 mL/min: 0-20 min, 18 mM NaOH; 20.1-35 min, 100 mM NaOH; 35.1-50 min, 18 mM NaOH.

Enzymatic reactions (20 μL) were also performed against complex glycans and glycoproteins using 10 μM of enzyme and 5 μM of oligosaccharides or FA2G2 (5 ng/μL)^78^, PLA2 (1 mg/mL), IgG (1 mg/mL) untreated or treated with PNGase B035DRAFT_03341 (10 μM)^72^ or PNGaseF (5000 units/mL), respectively in 50 mM citrate buffer at pH 6, 37°C for 24 h to release *N*-glycans. The products were analysed by LC-FD-MS/MS as previously described^11^. The reactions were stopped by heating 95 °C for 5 min and then dried down using Savant SpeedVac centrifugal evaporator (Thermo Fisher, Wilmington, USA), labelled at the reducing end with procainamide using the glycan labelling kit with sodium cyanoborohydride as the reductant (Ludger, Oxford, UK) and purified using a LudgerClean Procainamide Plate (LC-PROC-96, Ludger, Oxford, UK) to remove the excess dye. The samples were dried down using a Thermo Savant SpeedVac centrifugal evaporator and resuspended in 50 µL of 75% acetonitrile: 25% water. The suspensions were then injected onto a Waters BEH amide column (2.1 ×150 mm, 1.7 µm particle size, 130 Å pore size) at 40 °C on a Dionex Ultimate 3000 UHPLC instrument with a fluorescence detector (λex = 310 nm, λem = 370 nm) coupled to a Bruker Amazon Speed ETD. A 50 mM ammonium formate solution pH 4.4 (Ludger, Oxford, UK) was used as mobile phase A and acetonitrile (Romil, UK) was used as mobile phase B. A 70 min gradient was used at 0.2 mL/min unless otherwise specified, 0-53.5 min, 76% to 51%B, 0.4 mL/min; 53.5-55.5 min, 51% to 0% B; 55.5-57.5 min, 0% B; 57.5-59.5 min, 0% to 76% B; 59.5-65.5 min, 76% B; 65.5-70 min, 76% B, 0.4 mL/min.

The Heatmap of enzyme specific activities was constructed via Chiplot (https://www.chiplot.online/). Hierarchical clustering was performed based on Euclidean distance calculated with complete linkage as computing method.

### Transfucosylation reactions

For transfucosylation, enzymatic reactions with 1 μM enzyme (1.43 μM for *Ba*GH29^26A^), 180 mM GlcNAc and 18 mM pNP-Fuc were incubated in 20% (v/v) DMSO to increase the solubility of pNP-Fuc for 1 h at 37°C. The reactions were stopped by addition of ethanol using three times the volume of the reaction. To assay the capacity of the enzymes to carry out further transfucosylation reactions, 1 μM enzyme (1.43 μM was used for *Ba*GH29^26A^) was incubated with 180 mM 3FN or 6FN and 18 mM pNP-Fuc in 20% (v/v) DMSO.

### Thin layer chromatography (TLC) and TLC-electrospray ionisation-mass spectrometry (TLC-ESI-MS) analysis

To analyse the products of transfucosylation reactions, standards, Fuc (0.01 µmol), pNP-Fuc (0.03 µmol), GlcNAc (0.25 µmol), 4FN (0.005 µmol), 3FN (0.005 µmol), 6FN (0.005 µmol), and reaction samples with GlcNAc (8 ×0.5 µL) or with 3FN or 6FN (4 ×0.5 µL) were loaded on a 12 cm tall plate (TLC Silica gel 60 F254, Sigma-Aldrich, Germany). The plates were developed using an isopropanol-ammonium hydroxide-water 6:3:1 mixture (namely IPA-NH_4_OH-H_2_O) for 3 h or until the frontline of the solvent rose to ca. 11.25 cm. The plate was then dried using a hair dryer and stained using a 5% ethanolic solution of sulphuric acid. Gently heating of the plate allowed the identification of the TLC spots corresponding to controls and reaction products.

TLC-ESI-MS of the enzymatic reactions was performed using an Expression Compact Mass Spectrometer (Advion, UK) coupled with a Plate Express reader (Advion, UK) in positive mode to identify the fucosylated reaction products. The enzymatic reactions were analysed through TLC as described above. The analysis was performed in duplicates to stain one TLC plate and use it as a guide to perform the TLC-ESI-MS on the non-stained plate. By comparison with the stained plate, the laser of the Plate Express reader was aimed at the right retention factor (R_*f*_) and the data obtained was analysed using Advion Mass Express software. Comparison of retention factors, in combination with TLC-ESI-MS analysis and NMR allowed identification of reaction products.

### Nuclear Magnetic Resonance (NMR) spectroscopy

An aliquot of the enzymatic reactions (600 μL) was evaporated to dryness and reconstituted in 600 μL of NMR buffer (100 mL D_2_O containing 0.26 g NaH_2_PO_4_, 1.41 g K_2_HPO_4_, and 1 mM deuterated trimethylsilyl propionate (TSP) as a reference compound) before ^1^H-NMR spectroscopic analysis. ^1^H-NMR spectra were recorded using a 600-MHz Bruker Avance spectrometer fitted with a 5-mm TCI proton-optimised triple resonance NMR inverse cryoprobe and autosampler (Bruker, Bremen, Germany). Sample temperature was controlled at 300 K. Spectra were acquired with 32 scans, a spectral width of 12500 Hz and an acquisition time of 2.6 s. The “noesypr1d” presaturation sequence was used to suppress the residual water signal with a low-power selective irradiation at the water frequency during the recycle delay. Spectra were then transformed with a 0.3-Hz line broadening and zero filling, manually phased, baseline corrected, and referenced by setting the TSP–d4 signal to 0 ppm. Reaction products were identified by comparison with the spectra of standards (GlcNAc, pNP-Fuc, Fucose, 6FN, 3FN and 4FN).

### STD NMR

All NMR binding experiments were performed at 278 K on a Bruker Avance III 800 MHz spectrometer equipped with a 5-mm TXI 800 MHz H-C/N-D-05 Z BTO probe (Bruker, Bremen, Germany). First, FA2G2 was spectroscopically characterised by standard COSY (*cosydfesgpph*), TOCSY (*mlvevphpp*), ^1^H-^13^C HSQC (*hsqctgpsp*) and NOESY (*noesygpph*) for the purpose of assignment. Then, FA2G2 was recovered and prepared in a Shigemi advanced NMR microtube assembly at the concentration of ~200 μM in the presence of ~20 uM *Ba*GH29^26A^ (protein:ligand ratio 1:20), in D_2_O buffer solution containing 25 mM Tris-d_11_ pH 7.8 and 100 mM NaCl. An STD NMR pulse sequence including 2.5 ms and 5 ms trim pulses and a 3 ms spoil gradient was used. Saturation was achieved by applying a train of 50 ms Gaussian pulses (0.40 mW) on the f2 channel, at 6.70 ppm (on-resonance experiments) and 40 ppm (off-resonance experiments). The broad protein signals were removed using a 40 ms spinlock (T1ρ) filter. As a first test for binding, an STD NMR experiment with a saturation time of 2 s and a relaxation delay of 5 s was performed. Then, an STD build up curve was performed, by carrying out STD experiments at different saturation times (0.5, 1, 2, 3, 4 and 5 s) with 2 K scans, in order to obtain the binding epitope mapping. The resulting build-up curves for each proton were fitted mathematically to a mono-exponential equation (*y*  =  a*[1-exp(b*x)]), from which the initial slopes (a*b) were obtained. Finally, the binding epitope mapping was obtained by dividing the initial slopes by the strongest signal corresponding to the methyl group of GlcNAc A, to which an arbitrary value of 100% was assigned.

### X-ray crystallography

*Ba*GH29^26A^ was dialysed into 20 mM Tris 150 mM NaCl. Sitting drop vapour diffusion plates were set up with a protein concentration of 20 mg/mL and 5 mM 2’FL. Crystals appeared in many conditions across commercial sparse matrix screens with the best diffracting crystals appearing in the following condition: 0.12 M diethylene glycol, 0.12 M triethylene glycol, 0.12 M tetraethylene glycol, 0.12 M pentaethylene glycol, 100 mM Tris(base)/bicine pH 8.5, 12.5% 2-methyl-2,4-pentanediol, 12.5% PEG 1000, 12.5% w/v PEG 3350. Diffraction datasets were collected at Diamond Light Source on beamline I24 at a wavelength of 0.9686 Å. Attempts were made to soak out the resulting Fuc molecule bound in the active site so that additional complexes could be attained. These attempts were unsuccessful. By seeding with WT *Ba*GH29^26A^ crystals we also grew diffracting crystals of *Ba*GH29^26A^D218N active site mutant. These crystals were grown in 0.1 M sodium acetate pH 4.6, 8% (w/v) PEG 4000. Data were processed using xia2^79^ and dials^80^. The phase problem was solved by molecular replacement using the search model 1ODU, prepared using Chainsaw^81^. Initial model building was performed using ArpWarp^82^, followed by alternating cycles of model building and refinement using coot^83^, refmac^84^, and PDBredo^85^. The refined WT *Ba*GH29^26A^ structure has 0.3% ramachandran outliers. The final D218N *Ba*GH29^26A^ structure has 0.05% ramachandran outliers.

### Reporting summary

Further information on research design is available in the Nature Portfolio Reporting Summary linked to this article.

### Supplementary information

Supplementary Information

Description of Additional Supplementary Files

Supplementary data 1

Supplementary data 2

Supplementary data 3

Supplementary data 4

Supplementary data 5

Supplementary data 6

Supplementary data 7

Reporting Summary

## Data Availability

The datasets generated and/or analysed during the current study are available from the corresponding author on reasonable request. Crystal structure data were submitted to PDB database with identifiers as 8P1S (supplementary data 1) and 8P1R (supplementary data 2). More data can be found in supplementary information. Other supplementary data includes: Supplementary data 1. PDB file for D218N Apo (8P1S). Supplementary data 2. PDB file for Fuc-bound WT (8P1R). Supplementary data 3. Taxonomy and clustering distribution of 34,258 non-redundant GH29 sequences. Supplementary data 4. Sequence representations of GH29 family colour-coded by SSN cluster ID allocation (maximum resolution). Supplementary data 5. Sequences of 2971 GH29s in the SSN Supplementary data 6. Sequences of 2796 GH29s for task training Supplementary data 7. Configuration of running environment for GH29BERT.

## Code availability

The source code and model parameters are available on GitHub: https://github.com/ke-xing/GH29BERT and Zenodo: https://zenodo.org/records/10614689, respectively. The GH29BERT model is accessible through a friendly user interface: https://huggingface.co/spaces/Oiliver/GH29BERT.
