# Supplementary material for: Exploring the sequence-function space of microbial fucosidases
Source: Commun Chem. 2024 Jun 18;7:137. doi: 10.1038/s42004-024-01212-4 (PMC11189522; doi:10.1038/s42004-024-01212-4)
Supplement: Supplementary file 1 — Supplementary Information [file 42004_2024_1212_MOESM1_ESM.pdf]

Supplementary information for:

Exploring the sequence-function space of microbial fucosidases

Ana Martínez Gascueña, Haiyang Wu, Rui WangC. David Owen, Pedro J. Hernando, Serena Monaco, Matthew Penner, Ke Xing, Gwenaelle Le Gall, Richard Gardner, Didier NdehPaulina A. Urbanowicz, Daniel I. R. Spencer, Martin Walsh, Jesus Angulo, and Nathalie Juge

Supplementary Tables

Table S1. Origin and substrate specificities of GH29 enzymes functionally characterised in SSN clusters

Table S2. Kinetic parameters of GH29 fucosidases on CNP-Fuc.

Table S3. Substrate specificity of GH29 fucosidases

Table S4. X-ray crystallography data collection and refinement statistics of *Ba*GH29^{26A}

Table S5. Comparative performance evaluations and detailed experimental setups of pLMs

Table S6. Final enzyme concentration and incubation time used in enzymatic assays

Supplementary Figures

Figure S1. **SDS-PAGE of recombinant GH29 fucosidases.**

Figure S2. **HPAEC-PAD analysis of GH29 fucosidases reaction products with different fucosylated substrates.**

Figure S3. **LC-FD-MS/MS analysis of the product profile of GH29 fucosidase reaction on complex glycans.**

Figure S4. Close-up of *Ba*GH29^{26A} active site.

Figure S5. STD NMR full build-up curve analysis of the binding of FA2G2 to *Ba*GH29^{26A}.

Figure S6 Transfucosylation activity of GH29 fucosidases.

Figure S7. Schematic of the GH29BERT semi-supervised training approach used for protein sequence representation and SNN clustering prediction.

Supplementary references

Table S1 Origin and substrate specificities of GH29 enzymes functionally characterised in SSN clusters

	GenBank No.	Organism	Protein Name	Reported substrates hydrolyzed
Cluster 1 (GH29-B)	AAD10477.1	*Streptomyces* sp. 142	SsFuc	**LNFP-II-PA**, LNFP-III-PA, Fucα1,2-LNFP-II-PA [1]
	-	*Bifidobacterium pseudocatenulatum* MP80	Ga0224696_111927	2’FL, **LNFP-II**, LNFP-I, LNFP-III [2]
	EEG94253.1	*Roseburia inulinivorans* DSM 16841	RiFuc29	LeA [3]
	ACU93704.1	*Capnocytophaga ochracea*	H06	Lewis epitope from human N glycans and glycoproteins [4]
	UUB87425.1	Breast-fed infant faecal microbiome	Fuc39	LeA, LeB, LeX, LeY, 3FL [5]
	UUB87420.1	Breast-fed infant faecal microbiome	Fuc18	**LeA**, **LeB**, **LeX**, **LeY**, **3FL**, 2’FL [5]
	ADZ77236.1	*Sphingobacterium* sp.	F11	Lewis epitope from human N glycans [4]
	AFK02389.1	*Emticicia oligotrophica* DSM 17448	Eo0918	mono-sialylated-glactosylated biantennary N-glycan, core-substituted with α1,6-linked fucose [6]
	WP_243035407.1	*Ruminococcus gnavus* E1	E1_10125	pNP-Fuc, 3FL, LeA, **LeX**, sLeA, sLeX, human plasma N glycan, PDB: 6TR4 [7]
	ADK97204	*Prevotella melaninogenica*	E07	pNP-Fuc, Lewis epitope from human N glycans [4]
	CCG57154.1	*Brachyspira pilosicoli*	E01	Lewis epitope from human N glycans and glycoproteins [4]
	ACJ53394.1	*Bifidobacterium longum* subsp.	Blon_2336	3’FL, PDB: 3MO4 [8]; 3FL, transfucosylation [9]; LeX epitope from human N-glycans [4]

		infantis ATCC 15697		
	AAO79241.1	*Bacteroides thetaiotaomicron* VPI-5482	BT4136	pNP-Fuc, α-l-fucopyranosyl fluoride [10]
	AAO77299.1	*Bacteroides thetaiotaomicron* VPI-5482	BT2192	**LeX**, LeA, 3FL, LeY, LeB, 2’FL, pNP-Fuc [11]; PDB: 3EYP (to be published)
	AAO76732.1	*Bacteroides thetaiotaomicron* VPI-5482	BT1625	pNP-Fuc [10]; **LeX**, LeY, LeA, 3FL, LeB [12]
	-	*Bifidobacterium longum* subsp. *longum* SC596	BLNG_01263	α(1–3/4) Fuc linkages [13]
	BAH80310.1	*Bifidobacterium bifidum* JCM 1254	BbAfcB	**LNFP-II**, LeA, LeX, LNFP-III, LeY, 3FL, LeB [14], transfucosylation [15]
	ABG83106.1	*Clostridium perfringens* ATCC 13124	CpAfc2	**LeX**, **LeA**, PGM [16], transfucosylation [15]
	AAK76203.1	*Streptococcus pneumoniae* TIGR4	SpGH29	**LeY**, LeX, LeA, LeB, 3FL, PDB: 6ORG [17]
	ALJ46339.1	*Bacteroides ovatus* ATCC 8483	Bovatus_01698	PDB: 4ZRX (to be published)
	NP_180377.2	*Arabidopsis thaliana*	AtFUC1	**3FL-AB**, LNFP-II-AB, LeA glycan-PA [18]
Cluster 2 (GH29-A)	AAK43160.1+ AAK43159.1	*Sulfolobus solfataricus* P2	Ssα-fuc	pNP-Fuc, Fucα1-3Fucα-O-pNP, transfucosylation [19]; XyG [20]
	AFR68935.1	*Fusarium graminearum* PH-1	FgFCO1	Fucα1-2Galβ1-2Xyl [15,21];<br>2’FL [15,22], Fucα1-2Gal [22]; transglycosylation [15,23,24], PDB: 4NI3 [21]
	AAM42160.1	*Xanthomonas campestris* pv.*campestris* str. ATCC 33913	NixE	pNP-Fuc, core1,3fucose in plan N glycan [25];<br>2'FL, XyG nonasaccharide (XXFG) extracted from apple, XyG-rich fraction extracted from citrus peel [15]

	UUB87428.1	Breast-fed infant faecal microbiome	Fuc2358	**LeA**, **BgH-II, LeX**, **LeY**, **2’FL**, **6FN**, 3FL, BgH-I, LeB, BgA, BgB [5]
	EDY95436.1	*Bacteroides plebeius* DSM 17135	BpGH29	pNP-Gal, PDB: 7LJJ [26]
Cluster 3 (GH29-A)	CBM40947.1	*Paenibacillus thiaminolyticus*	aLfuk1	pNP-Fuc, transglycosylation [27], PDB: 6GN6 [28]
	AEX22740.1	*Vibrio* sp.strain EJY3	VejFCD	pNP-Fuc, pNP-α-D-Glc, pNP-α-D-Gal, pNP-β-D-Gal, lactose, maltose, PDB: 7DB5 [29]
	QEX52072.1	*Paenibacillus* sp.3179	PsFuc	pNP-Fuc, transfucosylate to produce 2'FL [30]
	MW767957	*Flavobacterium algicola*	OUC-Jdch16	pNP-Fuc, transfucosylate to produce 2'FL [31]
	A0A068B4M2	soil metagenome	Mfuc7	**2'FL**, XyG, 3FL [32]
	KJ626340	soil metagenome	Mfuc5	**XyG**, **2'FL**, 3FL [32]
	KJ626339	soil metagenome	Mfuc4	**XyG**, **2'FL**, 3FL [32]
	A0A068B4L8	soil metagenome	Mfuc2	**XyG**, **2'FL**, 3FL [32]
	A0A068B780	soil metagenome	Mfuc1	**XyG**, 2'FL, 3FL [32]
	ANW96113.1	*Wenyingzhuangia fucanilytica* CZ1127^T	FucWf2	pNP-Fuc, α-1,3/4-linked terminal L-fucose in sulfated fucooligosaccharides [33]
	ANW96121.1	*Wenyingzhuangia fucanilytica* CZ1127^T	FucWf1	pNP-Fuc, α-1,3/4-linked terminal L-fucose in sulfated fucooligosaccharides [33]
	UUB87423.1	Breast-fed infant faecal microbiome	Fuc35A	**LeX**, 2’FL, LeY, 6FN, LeA, 3FL, LeB [5]

	UUB87426.1	Breast-fed infant faecal microbiome	Fuc193	**LeX**, LeY, 6FN, LeA, 2’FL, 3FL [5]
	MW623633.1	*Paraglaciecola* sp.	Fp251	CNP-Fuc [34]
	MW623631.1	*Paraglaciecola* sp.	Fp239	CNP-Fuc, 2’FL, LeY, 3FL [34]
	MW623630.1	*Paraglaciecola* sp.	Fp231	CNP-Fuc, 4FN [34]
	ACD03857.1	*Akkermansia muciniphila* Muc^T (ATCC BAA-835)	Amuc_0010	pNP-Fuc, 2’FL [35], 3FN [36]
	ANW96380.1	*Wenyingzhuangia fucanilytica* CZ1127^T	Alf1_Wf	pNP-Fuc, LeA, α1,3 linkage in the fucoidan fragment [37]
	WP_004844769.1	*Ruminococcus gnavus* ATCC 29149	ATCC_03833	**pNP-Fuc**, 2’FL, 3FL, LeA, sLeA, FA2G2 [7]
Cluster 4 (GH29-A)	ACZ87343.1	*Streptosporangium roseum*	SrFucNaFLD	**4-Methylumbelliferyl-α-L-fucopyranoside**, 2'FL, BgH-II, LeA [38]
	KXK31601.1	*Omnitrophica* bacterium OLB16	Fucosidase O	**2'FL, NA2F**, M3N2F, NGA2F, M3N2XF, LNFP-II; xylosylated NGA2F [39]
	AFK04462.1	*Emticicia oligotrophica* DSM 17448	Eo3066	3'-FL, LNFP-III, LNDFH-I, LNDFH-II [6]
	AAA52481.1	*Homo sapiens*	HsFucA1	**4MU-Fuc**, **Fucα1-2Gal-,** pNP-Fuc, 2’FL,LNFP-II, LNFP-III, α1-2Fuc-dekasaccharide, 6FN [40]; PDB: 7PLS [41]
	AAM50292.1	*Drosophila melanogaster*	DmFuca	4MU-Fuc [42]
	AFR68934.1	*Fusarium oxysporum* 0685	FoFCO1	pNP-Fuc [43];<br>fucoidan, PGM [44]
	APG32842.1	*Fusarium proliferatum* LE1	FpFucA	pNP-Fuc, BgH-II-OMe, 4FN-PAA, 6-O-**α**-L-Fuc- *N,N'*-diacetyl-chitobiose, **α**-L-fucobiosides [45]
	BBG92283.1	*Patiria pectinifera*	Pap-Alf	**pNP-Fuc**, pNP-2-sulfo-α-Fuc, fucosyl-α-lactosides (Fuc-α1,2/3/6-Galβ1→4Glc-β-pNP) [46]

	CAA34268.1	*Rattus norvegicus*	RnFucA1	pNP-Fuc, α1-2/3/4 fucosyl linkages [47]
	CAA63362.1	*Canis lupus familiaris*	ClfFucA1	4MU-Fuc [48]; 2'FL, 3FL, LNFP-II, transfucosylation [49]
	CAB53746.1	*Homo sapiens*	HsFucA2	**2'FL**, Fucα1-2Gal/Glc/Xyl, 3FN, 4FN, 6FN, Fucα1-6Gal [50]
	AAO51149.1	*Dictyostelium discoideum*	DdFucA	**6FN**, 2'-Fucosyllactitol, 3-Fucosyllactitol, LNFP-I [22]
	BCX80342.1	*Bombyx mori*	BmFucA	**2'FL**, GlcNAcβ(1-4)[Fucα(1-6)]GlcNAc, 3FL, GlcNAcβ(1-4)[Fucα(1-3)]GlcNAc [51]
Cluster 5 (GH29-A)	AKH95075.1	*Elizabethkingia meningoseptica* FMS-007	cFase I	pNP-Fuc, 3'-FL, LeX, PLA2, HRP [52]
	ADB37178.1	*Spirosoma linguale* DSM74	SlFuc29	pNP-Fuc [53]
	UUB87421.1	Breast-fed infant faecal microbiome	Fuc19A	LeA, LeB, **LeX, LeY,** 3FL [5]
	AFK05193.1	*Emticicia oligotrophica* DSM 17448	Eo3812	3'-FL, LNFP III [6]
	CAH09273.1	*Bateroides fragilis* NCTC 9343	BF3591	**LeX, 3FN**, pNP-Fuc, Fucα1,2Gal, LeA, 6FN, 4FN [54]
Cluster 6 (GH29-A)	UUB87429.1	Breast-fed infant faecal microbiome	Fuc5372	**2'FL**, BgH-II, BgB, BgA, 6FN, 3FL, BgH-I [5]
	CAH06554.1	*Bacteroides fragilis* NCTC 9343	BF0810	pNP-Fuc [54]
Cluster 7 (GH29-A)	AAD35394.1	*Thermotoga maritima* MSB8	TmαFuc	**pNP-Fuc**, transglycosylation [55];<br>2'FL, XXFG, XyG [15,32]; PDB: 1HL8 [56]
	ACJ51546.1	*Bifidobacterium longum* subsp. *infantis* ATCC 15697	Blon_0426	**CNP-Fuc**, LNFP III [8]; 2'FL, 6FN, Fucα1-2Gal [57]
	ACJ51376.1	*Bifidobacterium longum* subsp. *infantis* ATCC 15697	Blon_0248	**CNP-Fuc**, LNFP III [8]; 6FN, Fucα1-2Gal [57]
Cluster 8 (GH29-A)	AEW21393.1	*Tannerella forsythia* ATCC 43037	TfFuc1 (TT1386)	**pNP-Fuc**, 2'FL [15,58], BgH-I, 6FN [58], XyG [15]

	QKE45427.1	*Pedobacter* sp. CAU209	PbFuc	**pNP-Fuc**, 3'FL,2'FL, transfucosylate to produce 2'FL/3'FL [59]
	CAH08937.1	*Bateroides fragilis* NCTC 9343	BF3242	**pNP-Fuc**, 3FN, 6FN, Fucα1-2Gal, 4FN, LeA, LeX, transfucosylate to produce 3FN/6FN [54]
Cluster 9 (GH29-A)	UUB87427.1	Breast-fed infant faecal microbiome	Fuc1584	**LeX**, **6FN**, LeY, 3FL, LeA, LeB, LNF-II [5]
Cluster 10 (GH29-A)	AAO78076.1	*Bacteroides thetaiotaomicron* VPI-5482	BT2970	**pNP-Fuc**, 3FN, 4FN [11]; PDB: 2WVV [60]
	CAH05807.1	*Bateroides fragilis* NCTC 9343	BF0028	**pNP-Fuc**, LeA, Fucα1-2Gal, 3FN [54]
Cluster 11 (GH29*)	UUB87422.1	Breast-fed infant faecal microbiome	Fuc30	6FN [5]
Cluster 13 (GH29-B)	UUB87424.1	Breast-fed infant faecal microbiome	Fuc35B	**LeA**, 3FL, LeX, LeB, LeY, LNF-II [5]
	NP_812709.1	Bacteroides thetaiotaomicron VPI-5482	BT3798	**pNP-Fuc** [4],PDB: 3GZA [60]
Cluster 16 (GH29-A)	CAQ67877.1	*Lactobacillus casei* BL23	AlfB	**3FN**, Fucα1-2Gal, 2'FL, BgH-II, 4FN [61]
Cluster 18 (GH29-A)	AIC77303.1	soil metagenome	Mfuc6	**XyG**, 2'FL, transfucosylation [32]
Cluster 20 (GH29-A)	ANW97462.1	*Wenyingzhuangia fucanilytica* CZ1127^T	FucWf6	Failed to express in soluble fraction [33]
Cluster 21 (GH29-A)	QTJ01949.1	*Paraglaciecola* sp.	Fp240	CNP-Fuc [34]
Cluster 23 (GH29-A)	AEW80941.1	*Propionibacterium acnes*	C11	pNP-Fuc, lewis epitope from human N glycans [4]
Cluster 26 (GH29-A)	CAQ67984.1	*Lactobacillus casei* BL23	AlfC	**6FN**, 4FN, Fucα1-2Gal, 3FN[61], transglycosylate to produce 6FN/6FN-Asn [62,63], PDB: 6O18 [64]
Cluster 34 (GH29-A)	QTJ01951.1	*Paraglaciecola* sp.	Fp284	CNP-Fuc, 2'FL, 6FN [34]
Cluster 41 (GH29-A)	CAQ67115.1	*Lactobacillus casei* BL23	AlfA	pNP-Fuc, 6FN [61]

Cluster 45 (GH29-B)	ABG82807.1	*Clostridium perfringens* ATCC 13124	Afc1 (TT4199)	n.a. [16]
Cluster 47 (GH29-A)	ANW96108.1	*Wenyingzhuangia fucanilytica* CZ1127^T	FucWf3	pNP-Fuc, α-1,2/3/4-linked terminal L-fucose in sulfated fucooligosaccharides [33]
Non-clustered	ANW96106.1	*Wenyingzhuangia fucanilytica* CZ1127^T	FucWf4	α-1,3/4-linked terminal L-fucose in sulfated fucooligosaccharides [33]

Substrates in bold are the most preferred ones among listed.

n.a., not active

-, not available

*, new GH29 subfamily

4MU-Fuc, 4 methy-lumbelliferyl-α-L-fucopyranoside

-AB, 2-aminobenzammide-labelled

-PA, 2-aminopyridin-labelled

pNP-Fuc, p-nitrophenyl-α-L- fucopyranoside

pNP-Gal, p- nitrophenyl-α-l-galactopyranoside

PGM, porcine gastric mucin

PA, 2-aminopyridine

XyG, xyloglucan

3FN, Fucα1-3GlcNAc

4FN, Fucα1-4GlcNAc

6FN, fucosyl-α-1,6-N-acetylglucosamine

BgH, blood group H

BgA, blood group A

BgB, blood group B

Table S2. Kinetic parameters of GH29 fucosidases on CNP-Fuc.

	Cluster	Name	Vmax (µM/min)	Km (µM)	kcat (min^{-1})	kcat/Km ($\mu M^{-1}\cdot min^{-1}$)
GH29-A	2	*Ri*GH29^{2A}	3.52 ± 0.03	302.30 ± 7.05	17605.00	58.24
	3	*La*GH29^{3A}	4.47 ± 0.20	208.10 ± 33.24	894.60	4.30
	3	*Rs*GH29^{3A}	12.13 ± 0.46	668.00 ± 69.04	12.13	0.02
	4	*Ny*GH29^{4A}	0.56 ± 0.01	279.80 ± 10.82	0.23	0.00
	8	*Tf*Fuc1^{8A}	0.53 ± 0.02	153.70 ± 21.81	534.70	3.48
	9	*Sg*GH29^{9A}	14.47 ± 1.74	495.20 ± 171.80	144.70	0.29
	26	*Ba*GH29^{26A}	1.45 ± 0.04	222.00 ± 20.10	726.50	3.27
	26	*Fb*GH29^{26A}	1.07 ± 0.05	188.10 ± 32.87	533.00	2.83
GH29-B	1	*Pg*GH29^{1B}	2.25 ± 0.02	302.10 ± 9.69	112.60	0.37
	1	*Sm*GH29^{1B}	2.60 ± 0.04	289.50 ± 14.61	51.90	0.18
	1	*Ss*Fuc^{1B}	4.78 ± 0.23	341.60 ± 51.67	4.78	0.01
	1	E1_10125^{1B}	1.93 ± 0.03	248.20 ± 12.27	20.04	0.07
	44	*Bs*GH29^{44B}	4.80 ± 0.13	299.60 ± 25.34	47.96	0.16
	45	Afc1^{45B}	-	-	-	-
	Non-clustered	*St*GH29^{nc}	2.19 ± 0.05	290.60 ± 19.81	0.44	0.00

*, unknown

-, not active

n.c., non-clustered

3 technical replicates were performed for each reaction.

Table S3. Substrate specificity of GH29 fucosidases

			Specific activity (U/μmol)												
	Cluster	Name	2’FL	3FL	BgA	BgB	BgH	LeA	sLeA	LeX	sLeX	LeY	6FN	pNP-Fuc	pPGM
GH29-A	2	*Ri*GH29^{2A}	399 ± 49	3.33 ± 0.12	na	na	454 ± 87	5.7 ± 0.9	0.167 ± 0.033	4.39 ± 0.95	0.366 ± 0.026	122 ± 16	0.099 ± 0.018	1210 ± 104	0.702 ± 0.053
	3	*La*GH29^{3A}	5.33 ± 0.65	0.727 ± 0.022	na	na	17.5 ± 1.6	0.281 ± 0.019	na	0.003 ± 0	na	0.474 ± 0.086	3.49 ± 0.43	91.4 ± 14	0.133 ± 0.018
	3	*Rs*GH29^{3A}	0.03 ± 0.005	na	na	na	0.042 ± 0.009	na	na	na	na	0.021 ± 0.004	0.022 ± 0.004	1.52 ± 0.28	0.03 ± 0
	4	*Ny*GH29^{4A}	0.019 ± 0.003	na	na	na	0.012 ± 0.001	na	na	na	na	0.009 ± 0.001	0.004 ± 0.001	0.165 ± 0.029	na
	8	*Tf*Fuc1^{8A}	3.38 ± 0.48	1.9 ± 0.42	na	na	43.5 ± 5.9	3.25 ± 0.49	0.167 ± 0.011	1.9 ± 0.25	0.486 ± 0.075	8.49 ± 1.69	41.1 ± 2.2	869 ± 99	0.401 ± 0.092
	9	*Sg*GH29^{9A}	0.032 ± 0.002	0.042 ± 0.006	na	na	0.037 ± 0.006	0.143 ± 0.005	0.006 ± 0.001	0.133 ± 0.011	0.012 ± 0.001	0.09 ± 0.017	849 ± 97	3.51 ± 0.69	na
	26	*Ba*GH29^{26A}	na	0.216 ± 0.03	na	na	na	0.194 ± 0.043	na	0.729 ± 0.093	na	0.373 ± 0.032	1005 ± 206	227 ± 19	na
	26	*Fb*GH29^{26A}	0.012 ± 0.001	0.906 ± 0.164	na	na	0.008 ± 0.001	2.01 ± 0.11	0.023 ± 0.003	0.066 ± 0.014	0.022 ± 0.004	0.577 ± 0.057	360 ± 49	315 ± 19	na
GH29-B	1	*Pg*GH29^{1B}	1.9 ± 0.29	535 ± 115	na	na	2.69 ± 0.48	1004 ± 28	28 ± 4.6	952 ± 156	112 ± 21	1063 ± 214	na	19 ± 2.7	0.005 ± 0.001
	1	*Sm*GH29^{1B}	1.1 ± 0.06	121 ± 9	na	na	1.18 ± 0.08	279 ± 53	0.07 ± 0.017	923 ± 49	0.386 ± 0.063	948 ± 169	na	19 ± 0.9	0.004 ± 0
	1	*Ss*Fuc^{1B}	0.168 ± 0.03	56.8 ± 4.6	na	na	0.021 ± 0.003	311 ± 30	1.45 ± 0.26	5.69 ± 0.28	2.47 ± 0.43	585 ± 114	na	0.183 ± 0.043	na
	1	E1_10125^{1B}	0.022 ± 0.003	70.7 ± 3.8	na	na	0.02 ± 0.002	72.6 ± 6.8	17.7 ± 3.9	40.1 ± 4.8	43.6 ± 0	111 ± 16	na	0.073 ± 0.013	0.026 ± 0.002
	44	*Bs*GH29^{44B}	na	na	na	na	na	na	na	na	na	na	0.014 ± 0.001	1.07 ± 0.21	na

45	Afc1^{45B}	na	0.011 ± 0.002	na	na	na	0.033 ± 0.002	na	0.072 ± 0.011	na	0.068 ± 0.013	0.006 ± 0.001	na	na
Non-clustered	*St*GH29^{nc}	na	0.067 ± 0.016	na	na	na	0.106 ± 0.017	na	0.234 ± 0.028	na	0.191 ± 0.02	0.25 ± 0.025	0.058 ± 0.007	na

Specific activity was calculated with 4 technical replicates for each GH29-substrate reaction. The enzyme was considered active against the tested substrates if over 6% of the substrates were hydrolysed within in 24 h. na, tested but no activity detected.

Table S4 X-ray crystallography data collection and refinement statistics of *Ba*GH29[26A]

	D218N Apo	Fuc-bound WT
PDB identifier	8P1S	8P1R
Data collection		
Space group	P21212	P21212
Cell dimensions		
a, b, c (Å)	87.7, 136.3, 160.5	89.1, 142.8, 167.2
α, β, g (	90, 90, 90	90, 90, 90
Resolution (Å)	103.86-1.66 (1.69-1.66)	108.56-1.90 (1.93-1.90)
R_{merge}	0.20 (0.8)	0.25 (9.9)
I/sigI	11.2 (1.9)	9.2 (0.4)
Completeness %	99.1 (94.5)	100 (100)
Redundancy	21.1 (7.8)	17.5 (14.9)
CC(½)	0.997 (0.541)	0.998 (0.215)
Refinement		
Resolution	104.07-1.66	83.74-1.90
No. reflections	224428	167720
R_{work}/R_{free}	0.193/0.160	0.201/0.176
No. atoms		
Protein	16417	16209
Ligand	40	122
Water	1942	749
B-factors		
Protein	16.8	27.6
Ligand	18.9	44.9
Waters	23.5	38.36
r.m.s deviations		
Bond lengths (Å)	0.014	0.008
Bond angles (°)	1.8	1.33

Table S5 Comparative performance evaluations and detailed experimental setups of pLMs. The pretrained pLMs include GH29BERT trained on around 30 K GH29 sequences, ESM-2 [65] and ProtT5-XL [66] trained on up to 65M and 45M sequences, respectively. Two baseline methods were also tested including GH29BERT without pre-training and one-hot encoding approach, respectively. The detailed hyper-parameters of model architectures including parameter scale, number of blocks in transformation encoders, representation dimensions, as well as the training data source and data amount, are presented.

Method		Pre-training						No Pre-training	
		GH29BERT	ESM-2				ProtT5-XL	GH29BERT	One-hot
Accuracy (%)		98.21	94.64	96.78	97.14	99.28	99.64	81.07	79.39
ECE Perplexity		1.09	1.13	1.13	1.33	1.03	1.04	1.82	2.10
Databases		C&I	UR50	UR50	UR50	UR50	UR50	C&I	C&I
Number proteins		~30 K	~65 M	~65 M	~65 M	~65 M	~45 M	-	-
Architect.	Parameter scale	20 M	8 M	35 M	150 M	650 M	1.2 B	20 M	-
	Number of blocks	5	6	12	30	33	24	5	-
	Embedding dimension	400	320	480	640	1280	1024	400	-
Training (PT / TT)	Optimizer	Adam	- / Adam					Adam	
	Batch size	4 / 1	- / 1					1	
	Learning rate	1e-7 / 1e-6	- / 1e-6					1e-6	
	Weight decay	0 / 0.001	- / 0.001					0.001	
	Epoch	100 / 600	- / 220	2000	2000	- / 50	- / 50	2000	

C, CAZy database; I, Interpro database; UR50, UniRef50 from UniProt database, PT, pre-training, TT, task-training.

Table S6 Final enzyme concentration and incubation time used in enzymatic assays

Enzymes\substrates	CNP-Fuc	2’FL	3FL	BgA	BgB	BgH	LeA	sLeA	LeX	sLeX	LeY	6FN	pNP-Fuc	pPGM
*Pg*GH29[1B]	0.02 µM; 40min	1 µM; 30 min	0.005 µM; 30 min	-	-	0.1 µM; 4 h	0.002 µM; 1 h	0.005 µM; 5 h	0.002 µM; 30 min	0.005 µM; 2 h	0.002 µM; 30 min	-	0.1 µM; 30 min	10 µM; 24 h
*Ri*GH29[2A]	0.0002 µM; 40min	0.01 µM; 30 min	1 µM; 30 min	-	-	0.001 µM; 2 h	0.5 µM; 30 min	1 µM; 5 h	0.5 µM; 30 min	1 µM; 2 h	0.01 µM; 30 min	5 µM; 2 h	0.001 µM; 30 min	1 µM; 1 h
*Sm*GH29[1B]	0.05 µM; 40min	1.5 µM; 1 h	0.01 µM; 1 h	-	-	0.1 µM; 6 h	0.005 µM; 30 min	5 µM; 2 h	0.001 µM; 1 h	1 µM; 2 h	0.001 µM; 1 h	-	0.01 µM; 6 h	10 µM; 24 h
*Ss*Fuc[1B]	1 µM; 40min	5 µM; 1 h	0.02 µM; 1 h	-	-	10 µM; 6 h	0.005 µM; 1 h	0.5 µM; 1 h	0.1 µM; 2 h	0.5 µM; 1 h	0.001 µM; 1 h	-	1 µM; 5 h	-
*Tf*Fuc1[8A]	0.001 µM; 25min	0.2 µM; 1 h	1 µM; 30 min	-	-	0.05 µM; 30 min	0.5 µM; 1 h	1 µM; 5 h	0.2 µM; 2 h	1 µM; 2 h	0.1 µM; 1 h	0.05 µM; 30 min	0.001 µM; 1 h	1 µM; 2 h
*La*GH29[3A]	0.005 µM; 40min	0.05 µM; 3 h	5 µM; 30 min	-	-	0.05 µM; 1 h	5 µM; 1 h	-	10 µM; 24 h	-	1 µM; 2 h	0.2 µM; 1 h	0.005 µM; 2 h	10 µM; 30 min
*Ba*GH29[26A]	0.002 µM; 40min	-	5 µM; 1 h	-	-	-	5 µM; 1 h	-	1 µM; 1 h	-	1 µM; 3 h	0.001 µM; 1 h	0.001 µM; 5 h	-
*Fb*GH29[26A]	0.002 µM; 40min	10 µM; 8 h	2 µM; 30 min	-	-	10 µM; 16 h	1 µM; 30 min	10 µM; 6 h	5 µM; 2 h	10 µM; 5 h	1 µM; 1 h	0.005 µM; 30 min	0.001 µM; 3 h	-
RsGH29[3A]	1 µM; 40min	10 µM; 4 h	-	-	-	10 µM; 2 h	-	-	-	-	8 µM; 6 h	8 µM; 6 h	1 µM; 30 min	10 µM; 20 h
Afc1[45B]	not detectable	-	5 µM; 24 h	-	-	-	10 µM; 3 h	-	10 µM; 2 h	-	8 µM; 2 h	8 µM; 24 h	-	-

*Sg*GH29^{9A}	0.1 µM; 40min	10 µM; 4 h	10 µM; 3 h	-	-	10 µM; 2 h	10 µM; 1 h	10 µM; 16 h	10 µM; 1 h	10 µM; 6 h	10 µM; 1 h	0.001 µM; 1 h	0.5 µM; 30 min	-
*Bs*GH29^{44B}	0.1 µM; 40min	-	-	-	-	-	-	-	-	-	-	10 µM; 8 h	1 µM; 1 h	-
*St*GH29^{nc}	5 µM; 40min	-	2.5 µM; 6 h	-	-	-	2.5 µM; 6 h	-	5 µM; 1 h	-	1 µM; 6 h	1 µM; 4 h	5 µM; 3 h	-
*Ny*GH29^{4A}	2.5 µM; 40min	5 µM; 16 h	-	-	-	10 µM; 6 h	-	-	-	-	8 µM; 16 h	10 µM; 20 h	1 µM; 6 h	-
E1_10125^{1B}	0.1 µM; 40min	10 µM; 6 h	0.05 µM; 30 min	-	-	10 µM; 4 h	0.01 µM; 2 h	0.01 µM; 5 h	0.05 µM; 30 min	0.05 µM; 30 min	0.01 µM; 1 h	-	5 µM; 3 h	1 µM; 24 h

-, specific activity could not be determined (the enzyme was considered inactive against the tested substrates if less than 6% of the substrates were hydrolysed within in 24 h).

Figure S1. **SDS-PAGE of recombinant GH29 fucosidases.** Lanes 1-7/9-15 correspond to the purified recombinant GH29 fucosidases. Lane 8 corresponds to the molecular weight marker (PageRuler™ Prestained Protein Ladder, 10 to 180 kDa). The gel was stained with InstantBlue Coomassie Protein Stain.

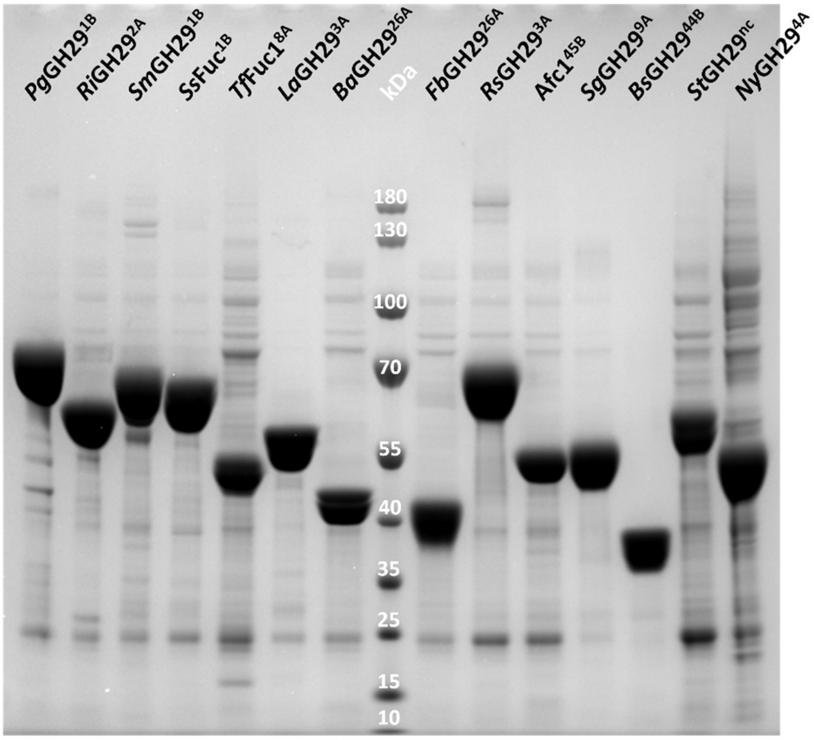

Figure S2. **HPAEC-PAD analysis of GH29 fucosidases reaction products with different fucosylated substrates.** The data were analysed with Prism. Standards: Fuc (red), fucosylated glycans (green for the substrates used; blue for the potential reaction products). The traces corresponding to the enzymatic reaction with GH29 enzymes in the presence of different substrates or in buffer control are shown in black. Substrates: 2'FL (A), 3FL (B), BgA (C), BgB (D), BgH (E), LeA (F), sLeA (G), LeX (H), sLeX (I), LeY (J), pNP-Fuc (L) and pPGM (M).

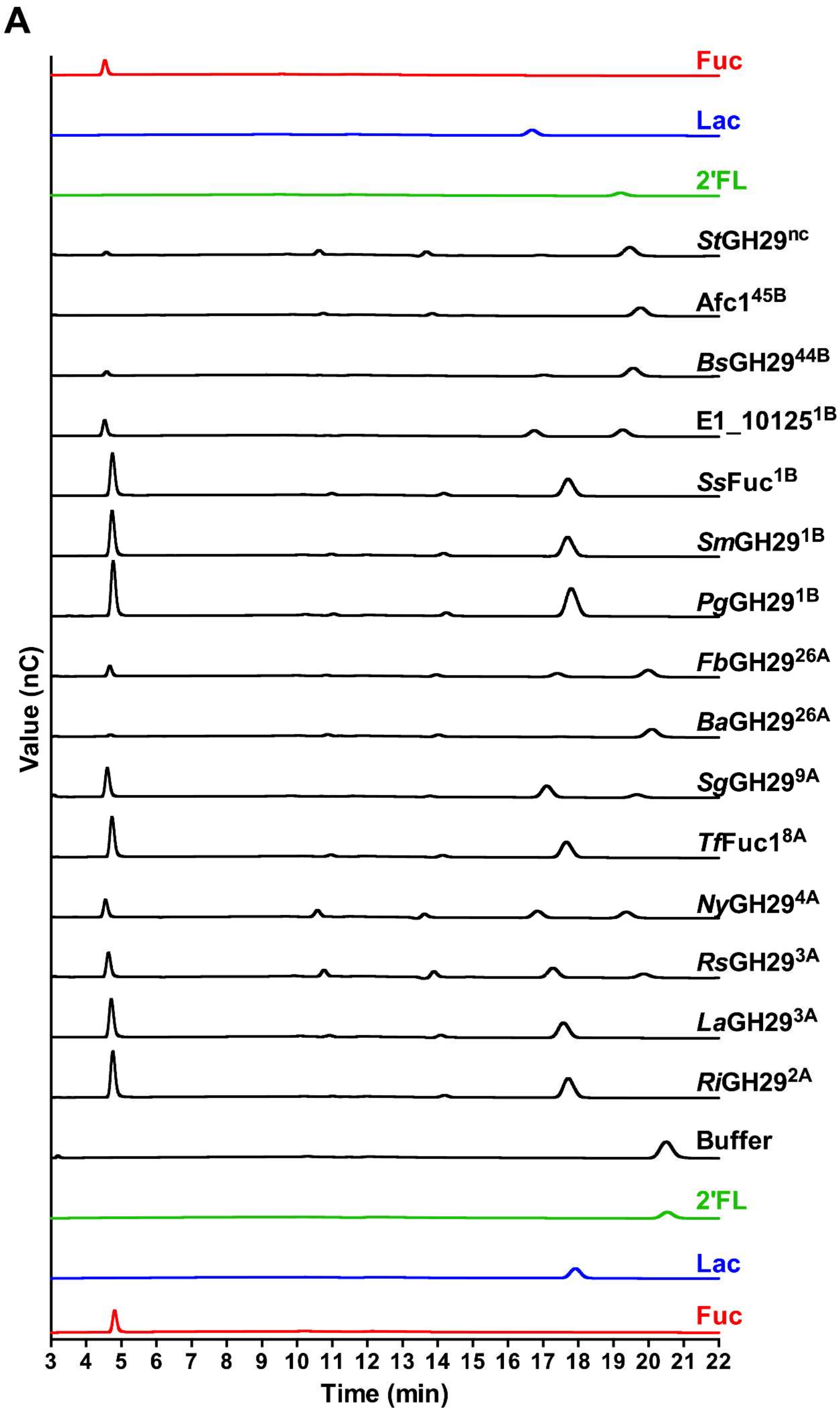
A
Fuc
Lac
2'FL
StGH29nc
Afc145B
BsGH2944B
E1_101251B
SsFuc1B
SmGH291B
PgGH291B
FbGH2926A
BaGH2926A
SgGH299A
TfFuc18A
NyGH294A
RsGH293A
LaGH293A
RiGH292A
Buffer
2'FL
Lac
Fuc
Value (nC)
3 4 5 6 7 8 9 10 11 12 13 14 15 16 17 18 19 20 21 22
Time (min)

B

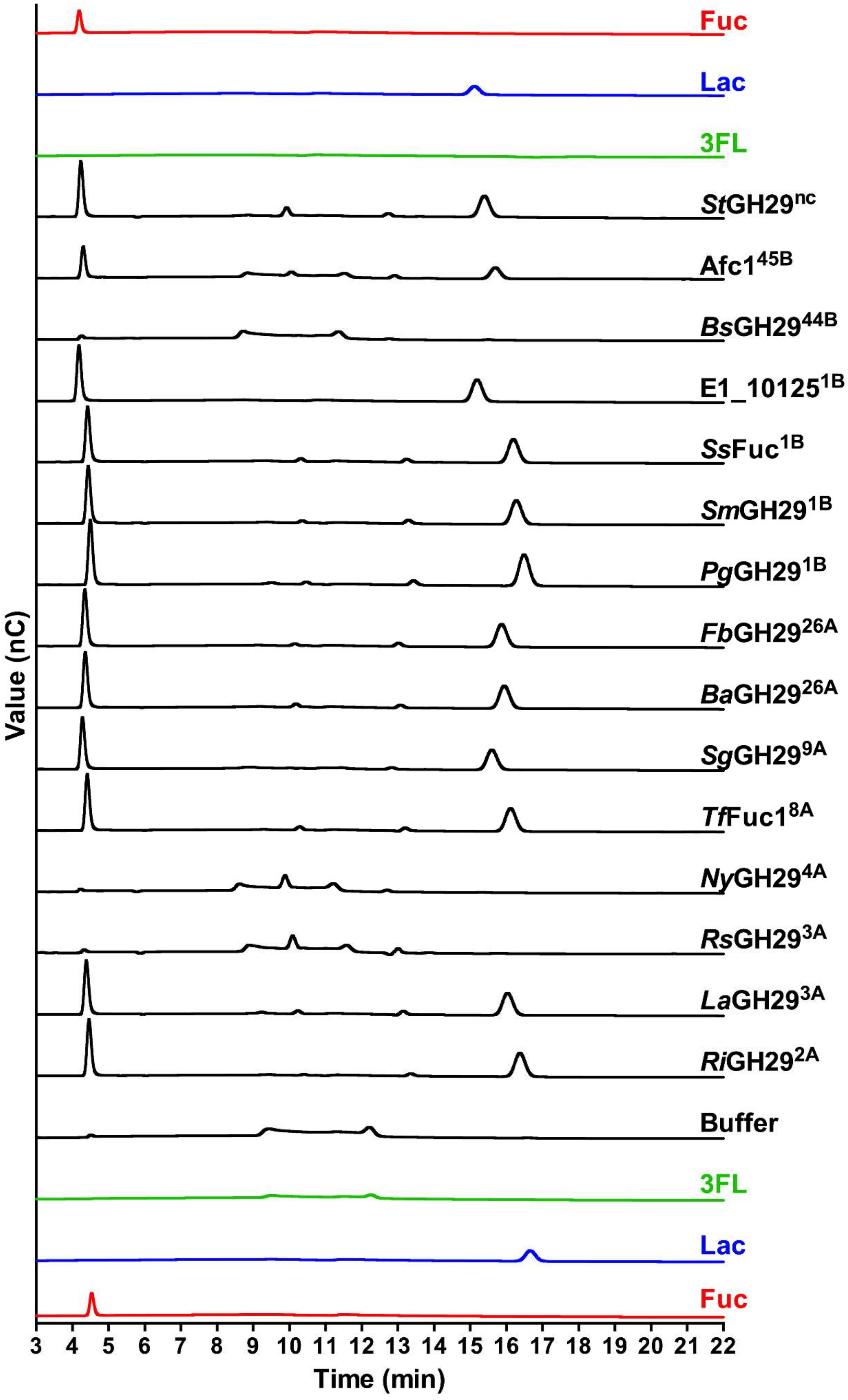
Fuc
Lac
3FL
StGH29nc
Afc145B
BsGH2944B
E1_101251B
SsFuc1B
SmGH291B
PgGH291B
FbGH2926A
BaGH2926A
SgGH299A
TfFuc18A
NyGH294A
RsGH293A
LaGH293A
RiGH292A
Buffer
3FL
Lac
Fuc
Value (nC)
3 4 5 6 7 8 9 10 11 12 13 14 15 16 17 18 19 20 21 22
Time (min)

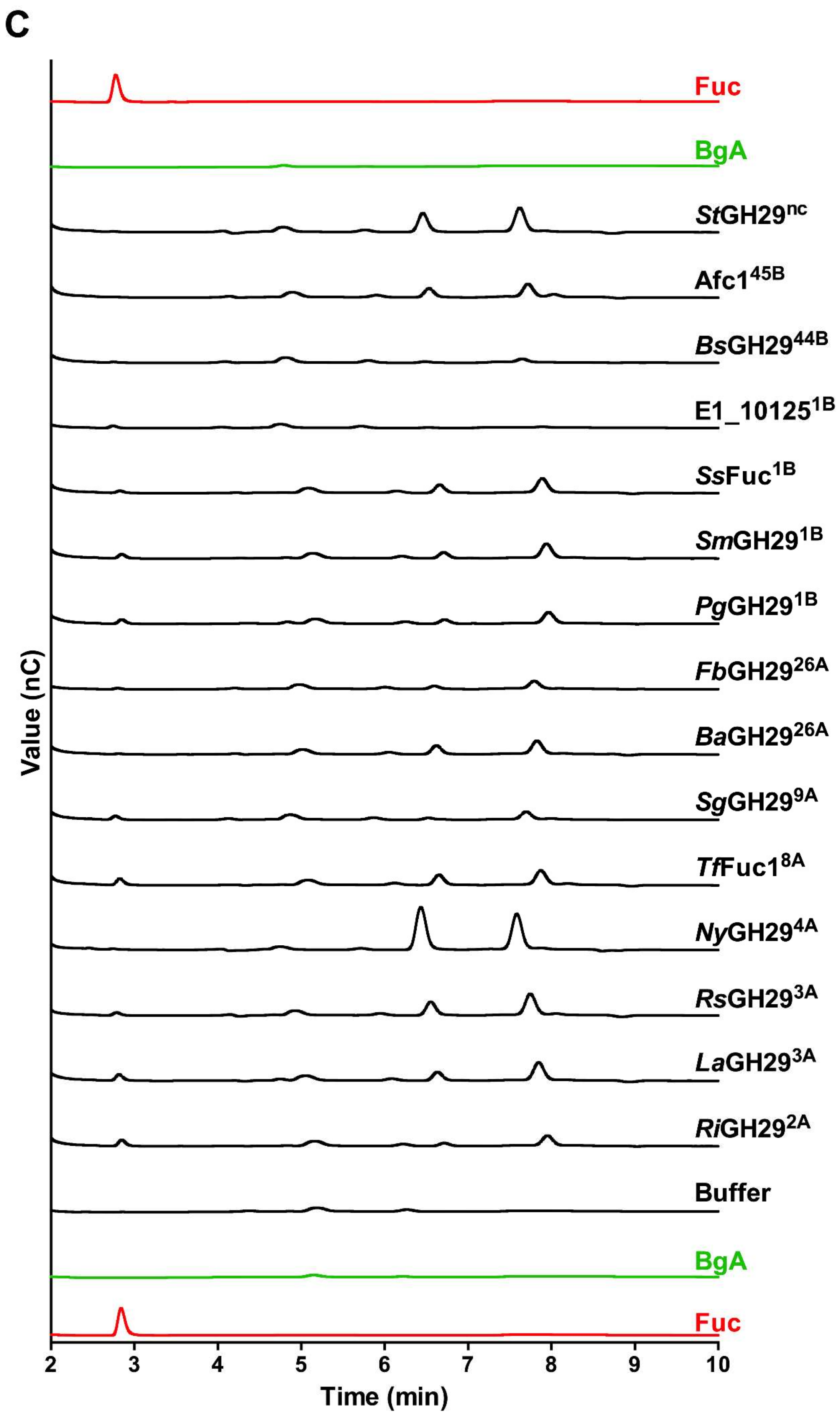

C
Fuc
BgA
StGH29nc
Afc145B
BsGH2944B
E1_101251B
SsFuc1B
SmGH291B
PgGH291B
FbGH2926A
BaGH2926A
SgGH299A
TfFuc18A
NyGH294A
RsGH293A
LaGH293A
RiGH292A
Buffer
BgA
Fuc
Value (nC)
2
3
4
5
6
7
8
9
10
Time (min)

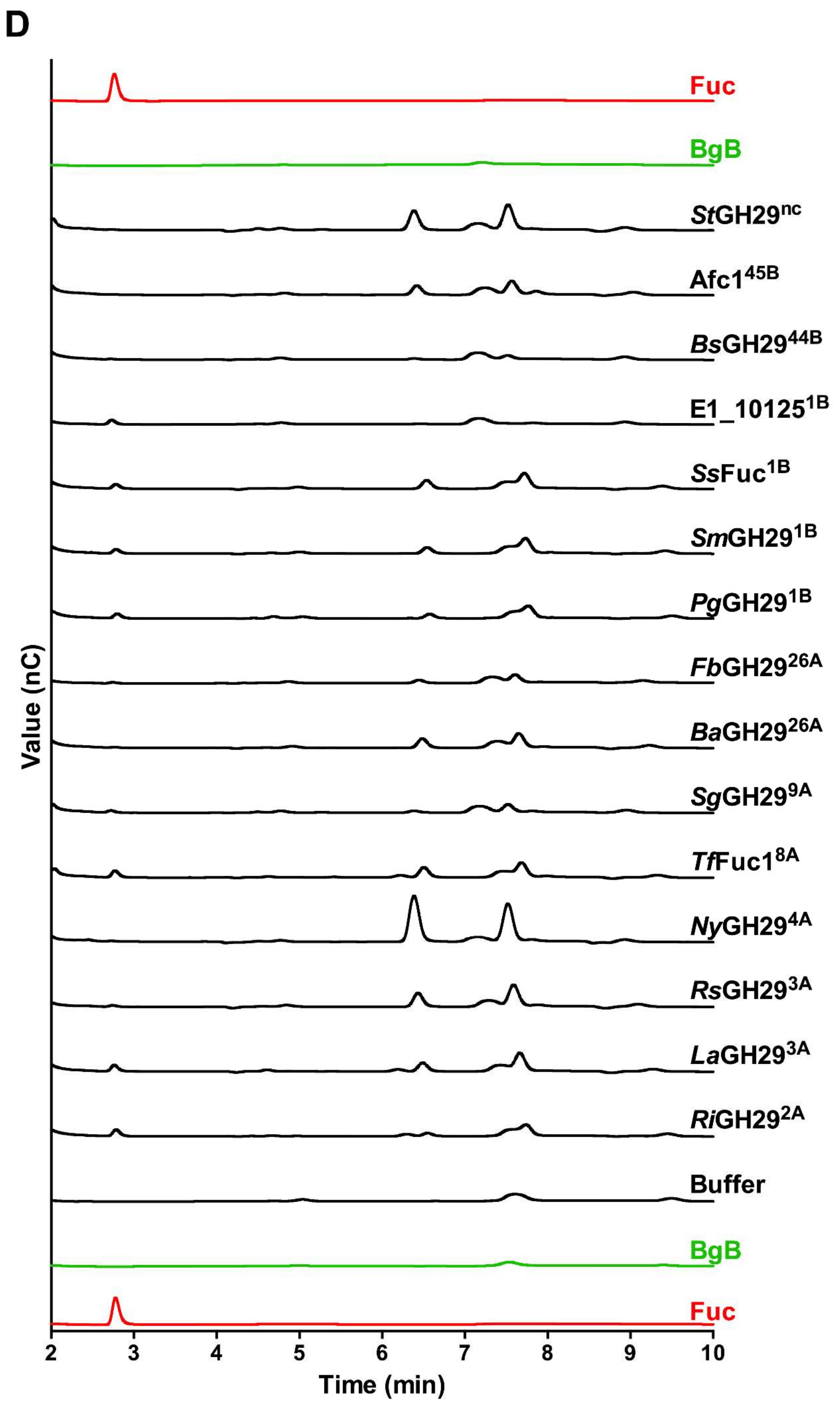
D
Fuc
BgB
StGH29nc
Afc145B
BsGH2944B
E1_101251B
SsFuc1B
SmGH291B
PgGH291B
FbGH2926A
BaGH2926A
SgGH299A
TfFuc18A
NyGH294A
RsGH293A
LaGH293A
RiGH292A
Buffer
BgB
Fuc
Value (nC)
2 3 4 5 6 7 8 9 10
Time (min)

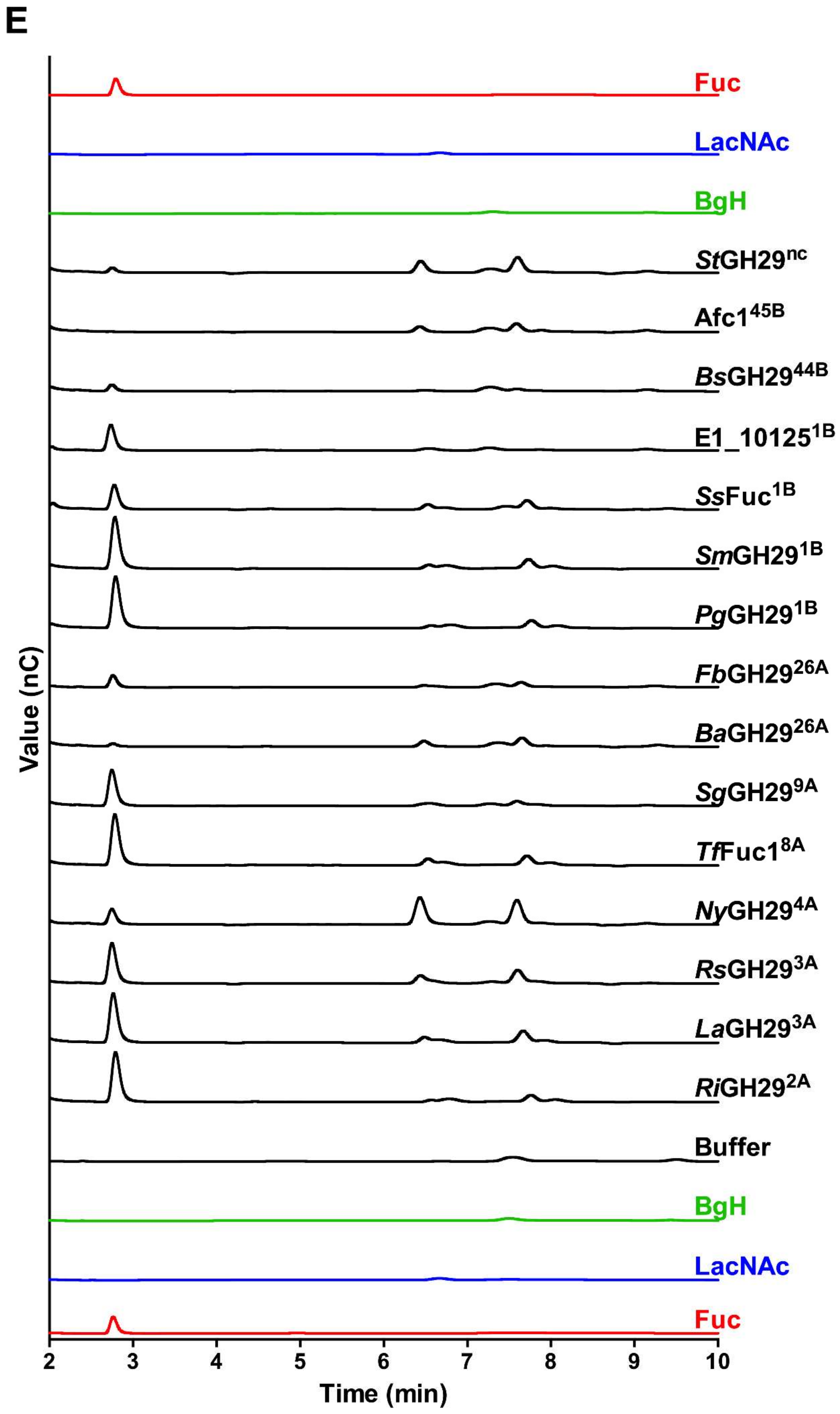

E
Fuc
LacNAc
BgH
StGH29nc
Afc145B
BsGH2944B
E1_101251B
SsFuc1B
SmGH291B
PgGH291B
FbGH2926A
BaGH2926A
SgGH299A
TfFuc18A
NyGH294A
RsGH293A
LaGH293A
RiGH292A
Buffer
BgH
LacNAc
Fuc
Value (nC)
2 3 4 5 6 7 8 9 10
Time (min)

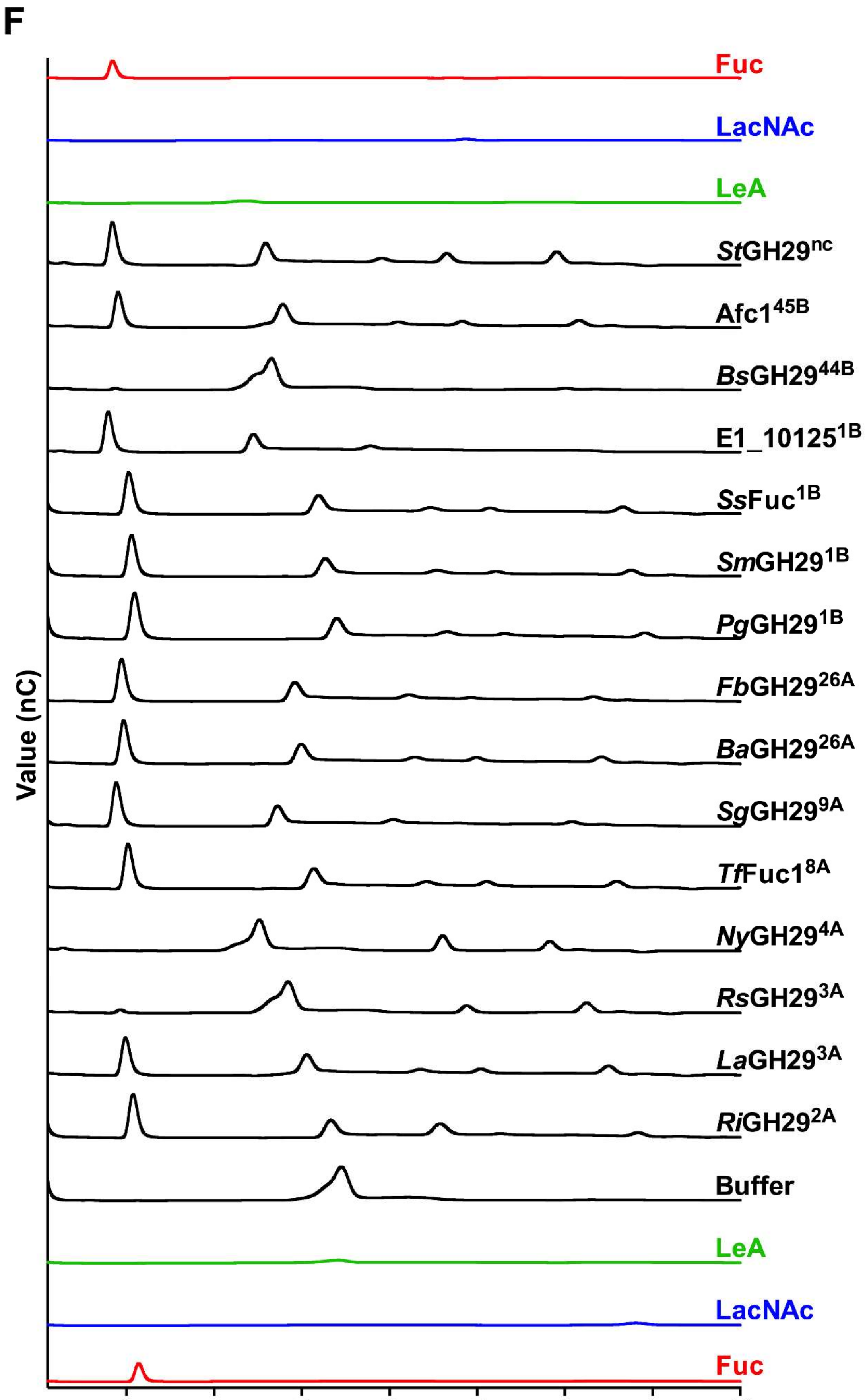

F
Fuc
LacNAc
LeA
StGH29nc
Afc145B
BsGH2944B
E1_101251B
SsFuc1B
SmGH291B
PgGH291B
FbGH2926A
BaGH2926A
SgGH299A
TfFuc18A
NyGH294A
RsGH293A
LaGH293A
RiGH292A
Buffer
LeA
LacNAc
Fuc
Value (nC)
3
4
5
6
7
8
9
10
Time (min)

G

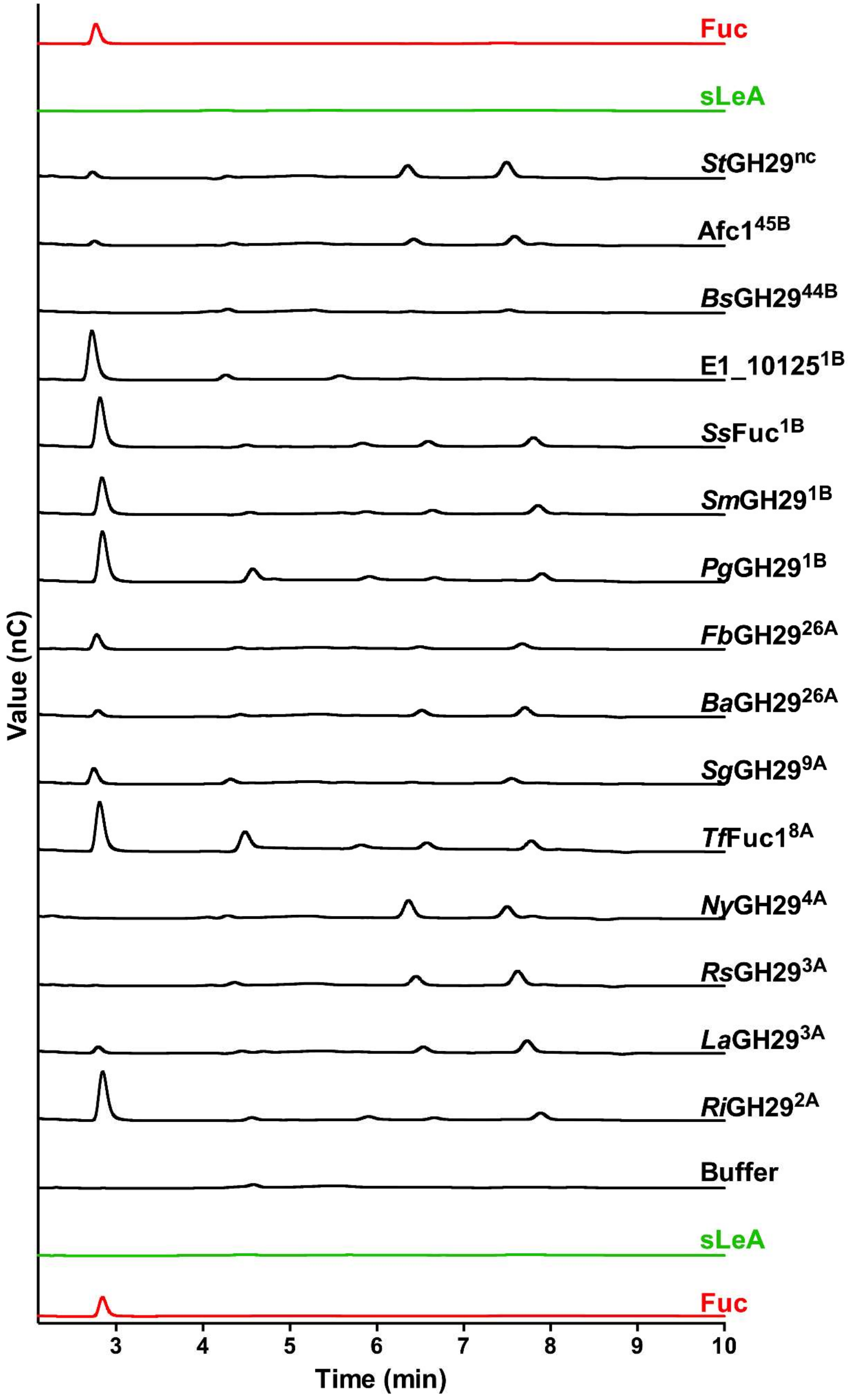

Fuc
sLeA
StGH29nc
Afc145B
BsGH2944B
E1_101251B
SsFuc1B
SmGH291B
PgGH291B
FbGH2926A
BaGH2926A
SgGH299A
TfFuc18A
NyGH294A
RsGH293A
LaGH293A
RiGH292A
Buffer
sLeA
Fuc
Value (nC)
3
4
5
6
7
8
9
10
Time (min)

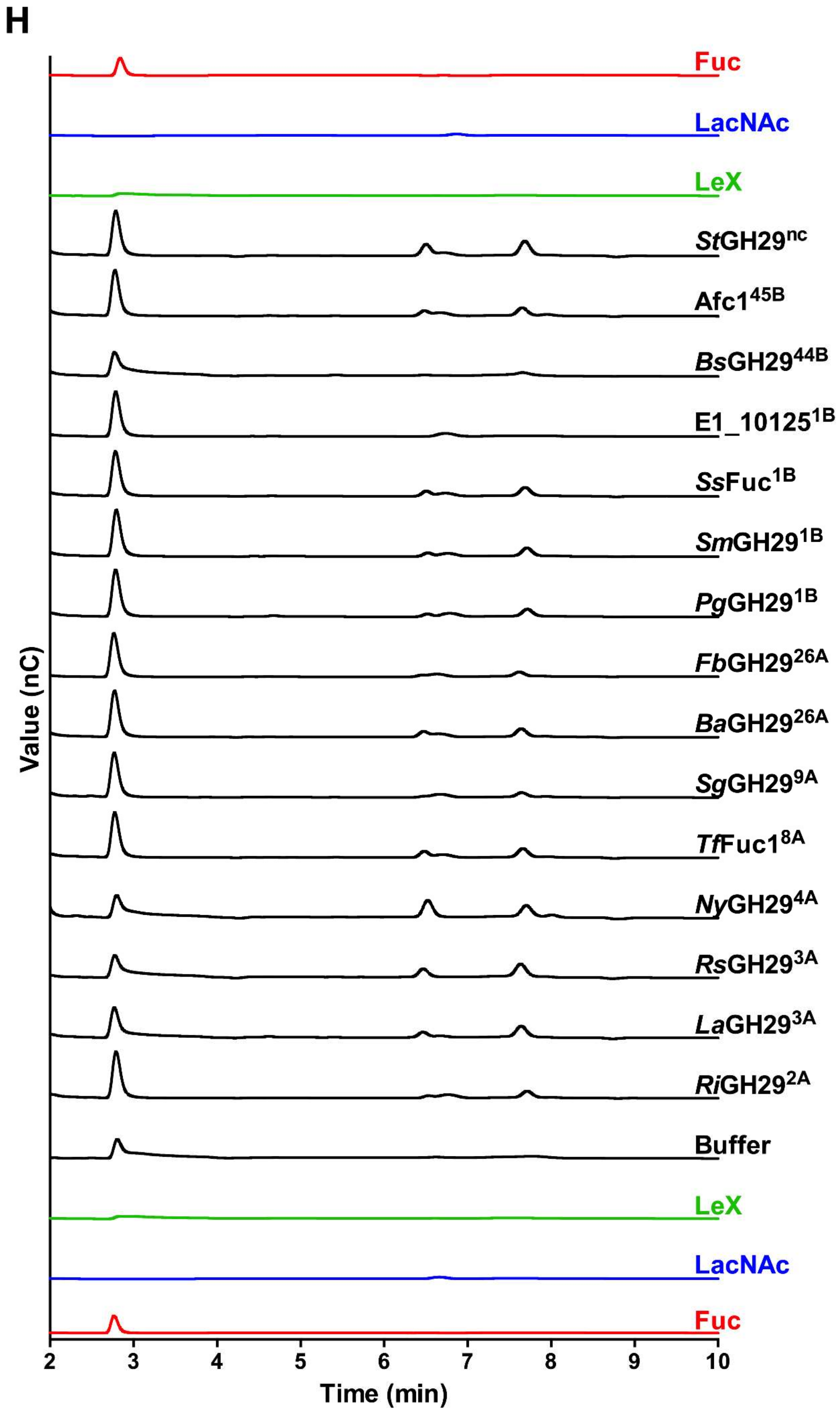

H
Fuc
LacNAc
LeX
StGH29nc
Afc145B
BsGH2944B
E1_101251B
SsFuc1B
SmGH291B
PgGH291B
FbGH2926A
BaGH2926A
SgGH299A
TfFuc18A
NyGH294A
RsGH293A
LaGH293A
RiGH292A
Buffer
LeX
LacNAc
Fuc
Value (nC)
2
3
4
5
6
7
8
9
10
Time (min)

I

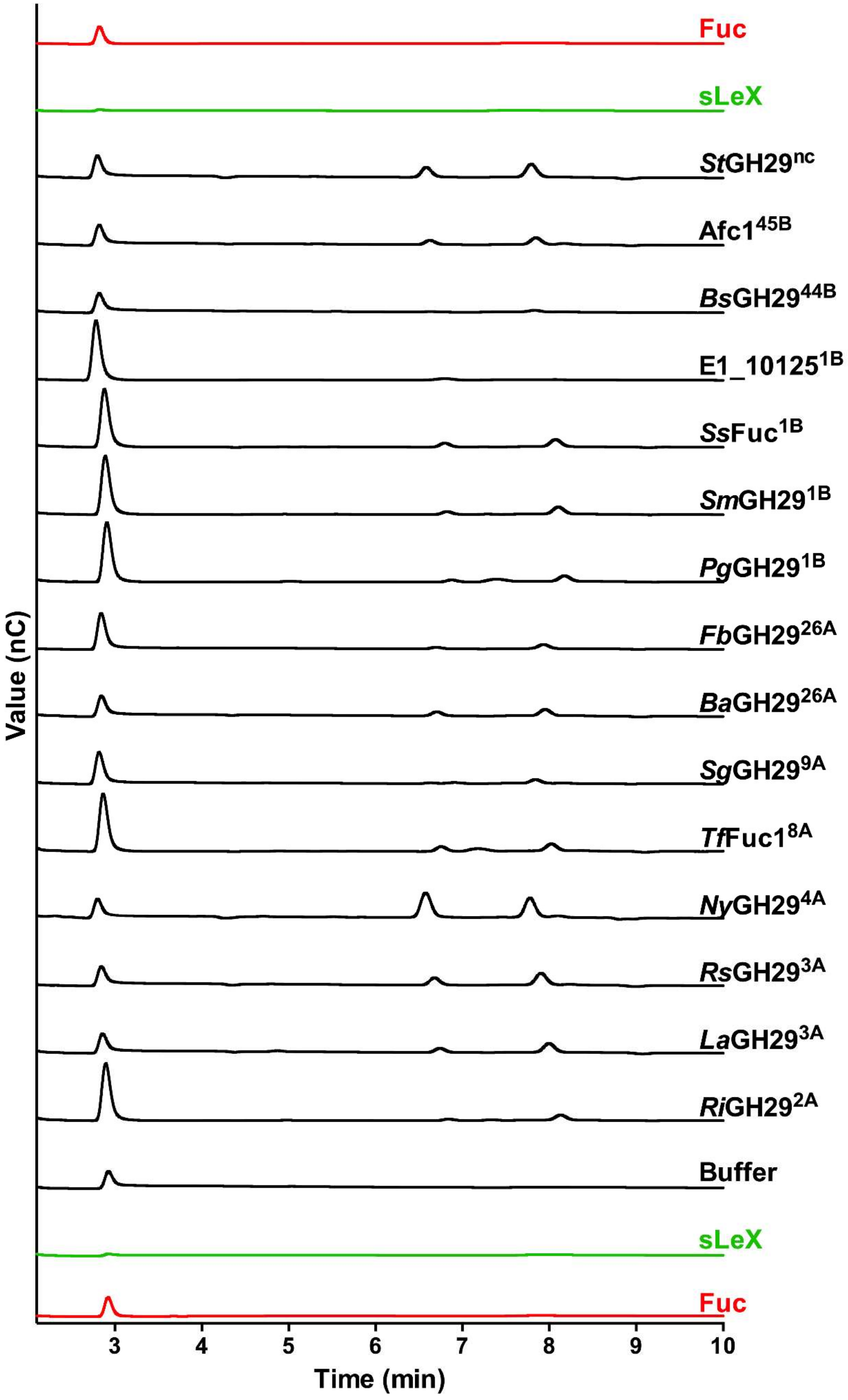
Fuc
sLeX
StGH29nc
Afc145B
BsGH2944B
E1_101251B
SsFuc1B
SmGH291B
PgGH291B
FbGH2926A
BaGH2926A
SgGH299A
TfFuc18A
NyGH294A
RsGH293A
LaGH293A
RiGH292A
Buffer
sLeX
Fuc
Value (nC)
3
4
5
6
7
8
9
10
Time (min)

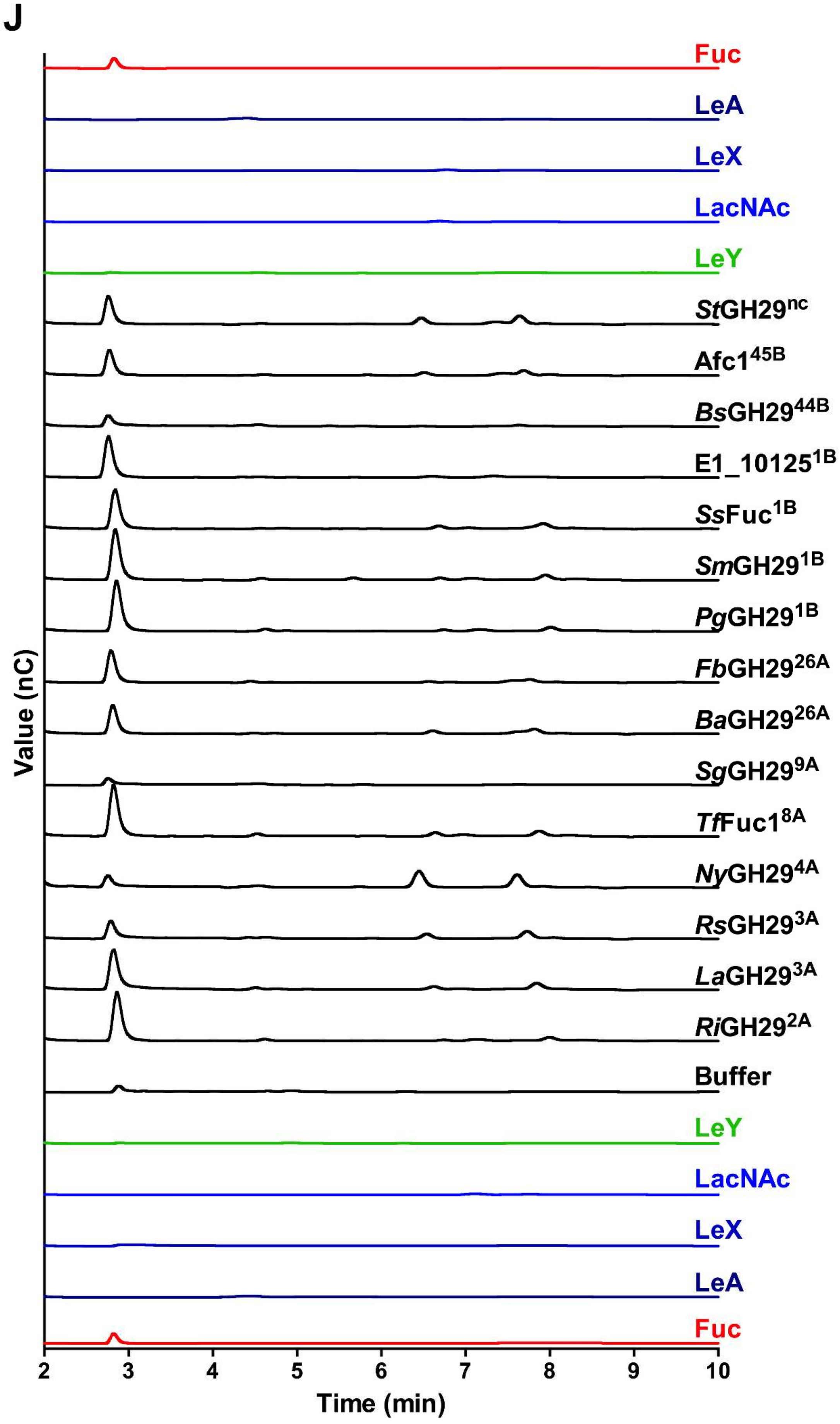
J
Fuc
LeA
LeX
LacNAc
LeY
StGH29nc
Afc145B
BsGH2944B
E1_101251B
SsFuc1B
SmGH291B
PgGH291B
FbGH2926A
BaGH2926A
SgGH299A
TfFuc18A
NyGH294A
RsGH293A
LaGH293A
RiGH292A
Buffer
LeY
LacNAc
LeX
LeA
Fuc
Value (nC)
2
3
4
5
6
7
8
9
10
Time (min)

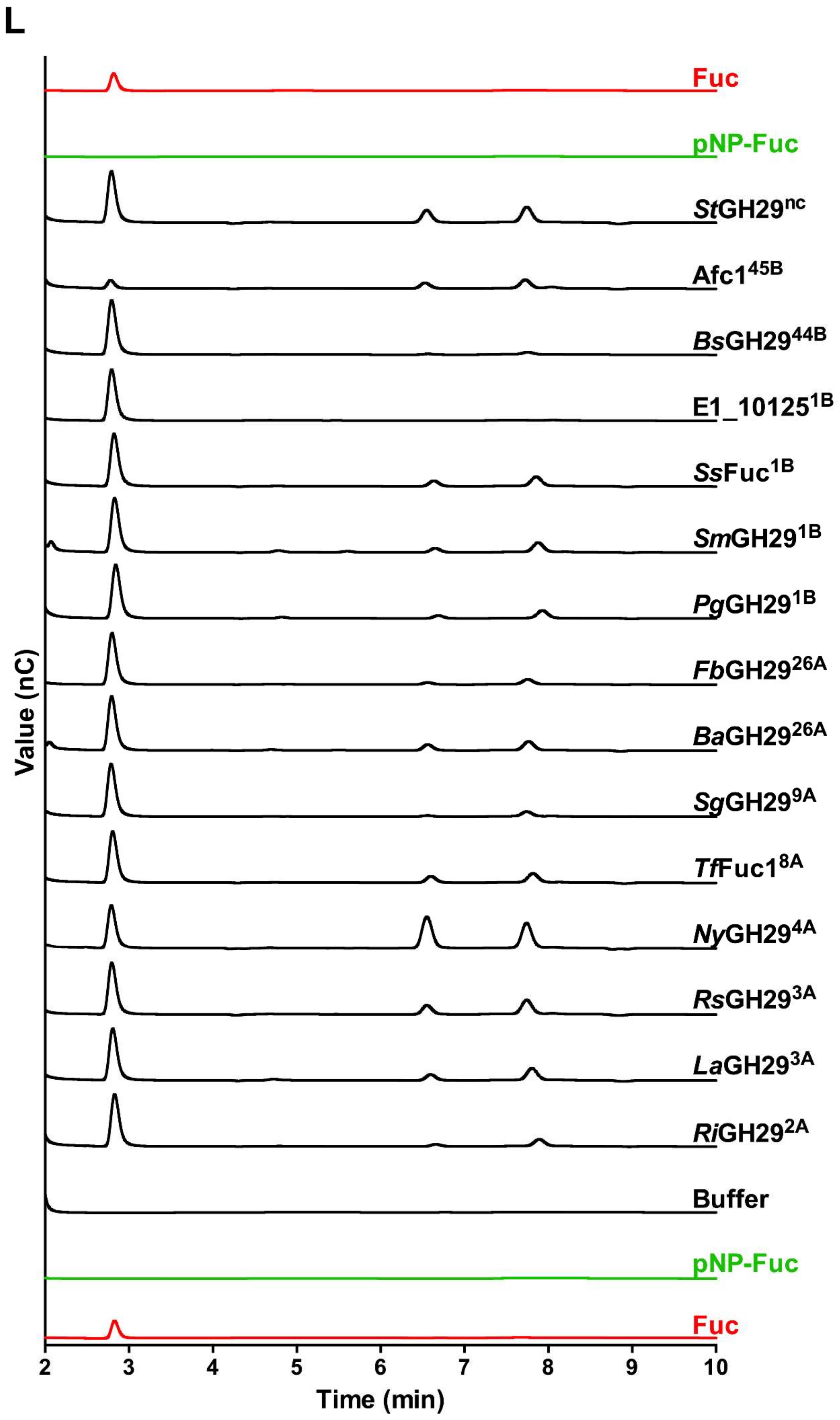

L
Fuc
pNP-Fuc
StGH29nc
Afc145B
BsGH2944B
E1_101251B
SsFuc1B
SmGH291B
PgGH291B
FbGH2926A
BaGH2926A
SgGH299A
TfFuc18A
NyGH294A
RsGH293A
LaGH293A
RiGH292A
Buffer
pNP-Fuc
Fuc
Value (nC)
2
3
4
5
6
7
8
9
10
Time (min)

M

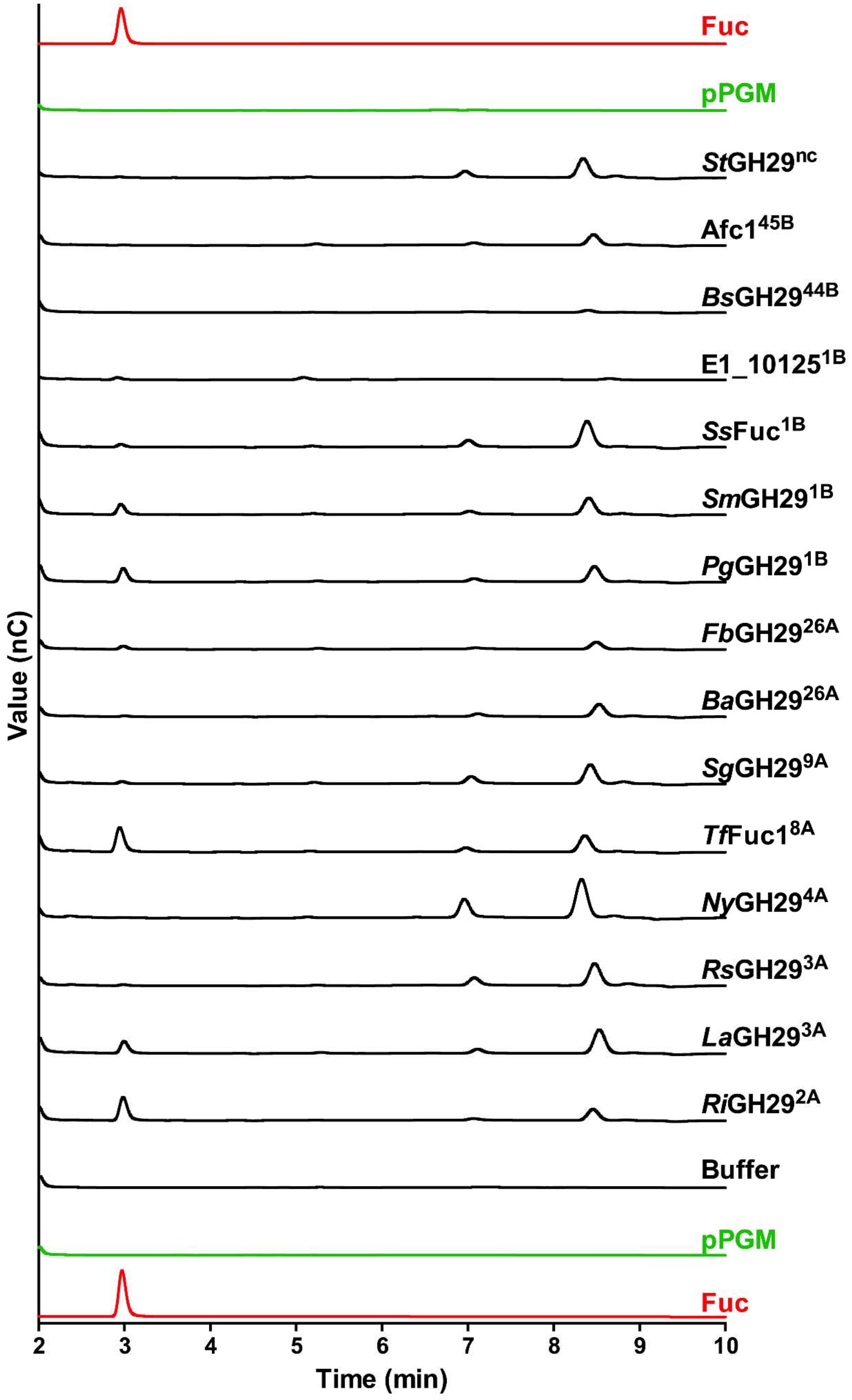
Fuc
pPGM
StGH29nc
Afc145B
BsGH2944B
E1_101251B
SsFuc1B
SmGH291B
PgGH291B
FbGH2926A
BaGH2926A
SgGH299A
TfFuc18A
NyGH294A
RsGH293A
LaGH293A
RiGH292A
Buffer
pPGM
Fuc
Value (nC)
2
3
4
5
6
7
8
9
10
Time (min)

Figure S3. **LC-FD-MS/MS analysis of the product profile of GH29 fucosidase reaction on complex glycans.** Enzymatic reactions with IgG *N*-glycans (**A**), IgG glycoprotein (**B**), PLA2 *N*-glycans (**C**), and PLA2 glycoprotein (**D**). Control samples were without enzymes. Glycan products are annotated next to peaks on the chromatograms.

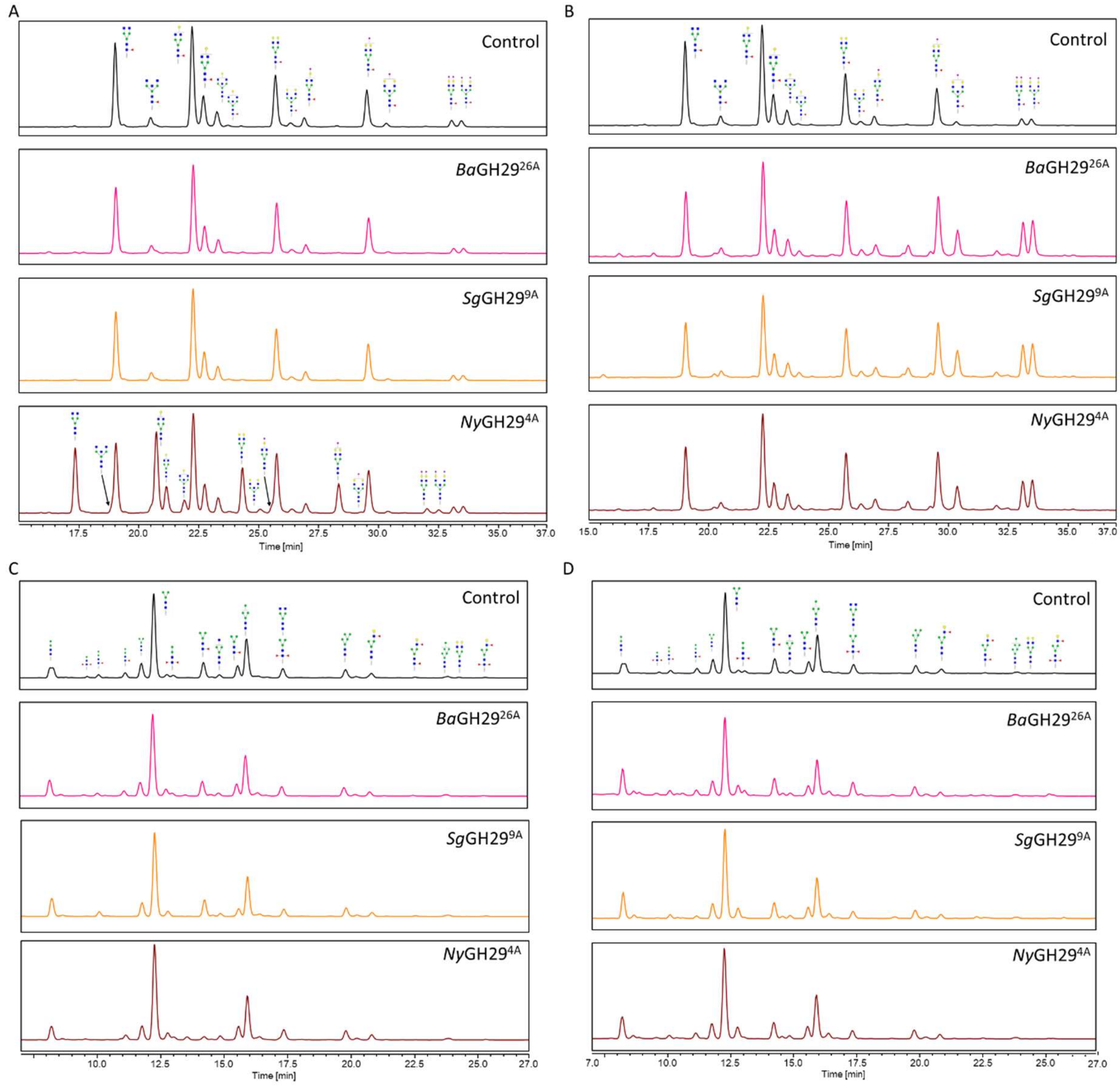

Figure S4. Close-up of *Ba*GH29[26A] active site. A) Fo-Fc difference map at 3σ (grey) and 5σ (grey) for fucose bound in the *Ba*GH29[26A] active site. **B**) Potential for plasticity in the *Ba*GH29[26A] active site highlighted by scaling and coloring the protein according to B factor. **C**) TT1819 (light blue) comparison with E1_10125[1B] GH29 (brown, PDB 6TR3). Residue numbering of *Ba*GH29[26A] or E1_10125[1B]. **D**) *Ba*GH29[26A] (light blue) comparison with α 1,3/4 GH29 Blon_2336 from *B. longum* subsp. *infantis* (orange, D172A/E217A mutant in complex with lacto-N-fucopentaose II, PDB 3UET; yellow, WT PDB 3MO4). Residue numbering of *Ba*GH29[26A] or Blon_2336. The hydrogen bond between Blon_2336 E237 and the ligand fucose residue is indicated.

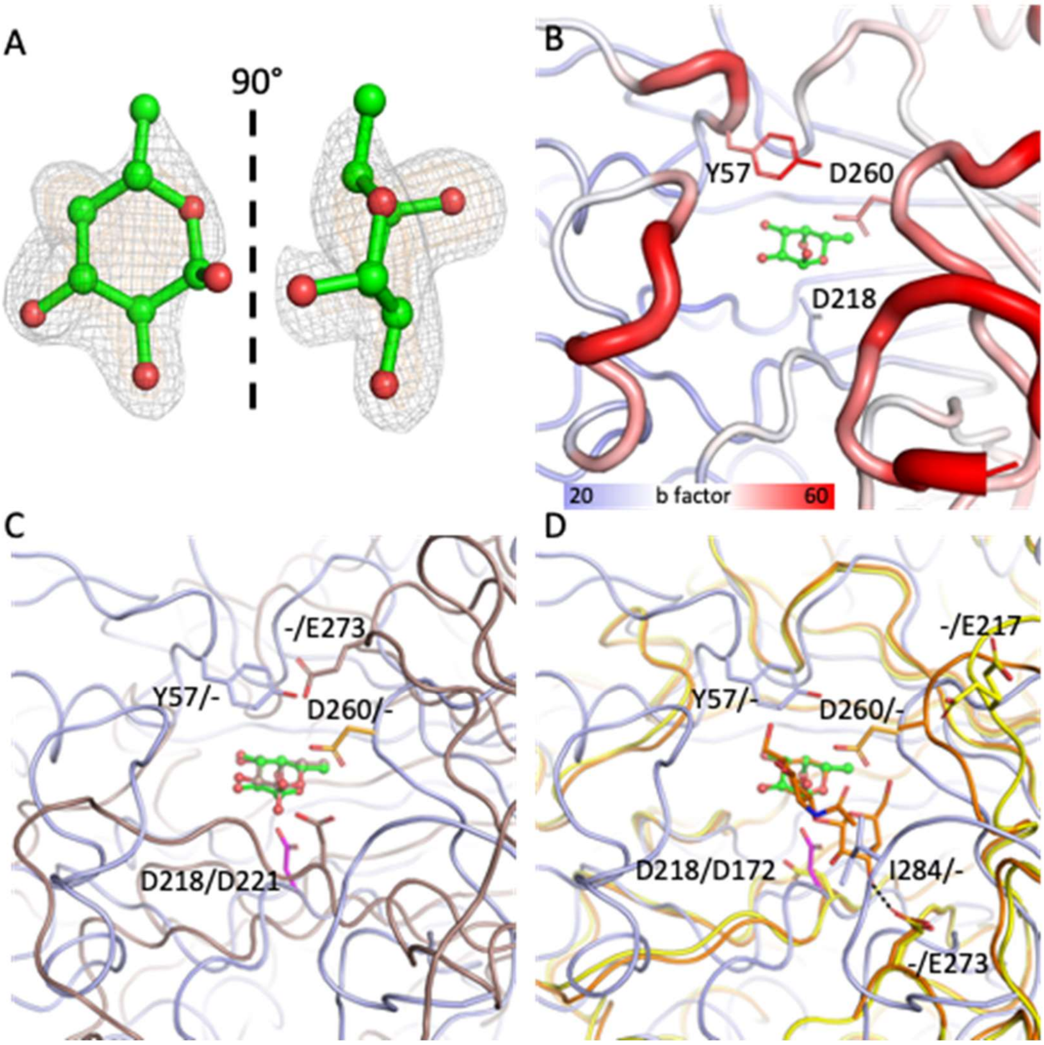

Figure S5. STD NMR full build-up curve analysis of the binding of FA2G2 to *Ba*GH29[26A]. A) Experimental STD NMR build-up curves. **B**) STD initial slopes values for the analysed protons, with contact intensities colouring scheme.

A

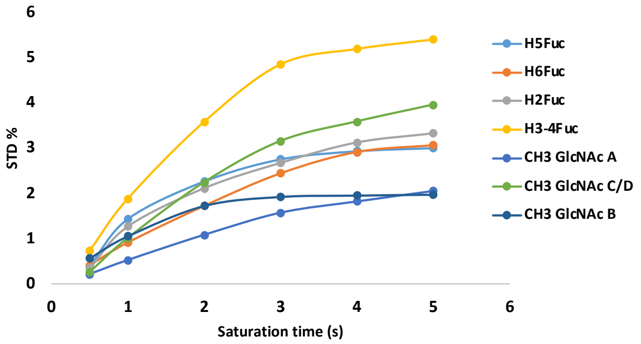

B

Proton identity	STD initial slope normalised	Legend
H2Man	undetected	0-30
H2Man'	undetected	31-60
H2Fuc	>45	61-100
H3-4Fuc	>57	
H5Fuc	70	
H6Fuc	63	
CH3 GlcNAc A	100	
CH3 GlcNAc B	53	
CH3 GlcNAc C/D	26	

Figure S6 Transfucosylation activity of GH29 fucosidases. A) TLC and TLC-ESI-MS analysis of ATCC_03833^{3A} transfucosylation reactions with GlcNAc, 3FN and 6FN as acceptors and pNP-Fuc as donor. Lanes 1 to 5 and 9 are standards: Fuc (lane 1), pNP-Fuc (lane 2), GlcNAc (lane 3), 4FN (lane 4), 3FN (lane 5) and 6FN (lane 9). Lanes 6 to 8 are ATCC_03833^{3A} transfucosylation reactions with GlcNAc (lane 6), 3FN (lane 7) and 6FN (lane 8). The upper gray dotted line corresponds to the 6FN standard and the lower blue dotted line corresponds to the 4FN standard. Glycan symbols follow the SNFG. **B**) TLC analysis of *Ri*GH29^{2A}, *La*GH29^{3A} and *Fb*GH29^{26A} transfucosylation reactions with GlcNAc as acceptor and pNP-Fuc as donor. Lanes 1 to 4 correspond to standards: GlcNAc (lane 1), 4FN (lane 2), 3FN (lane 3) 6FN (lane 4). Lanes 5 to 7 are transfucosylation reactions with GlcNAc using *Ri*GH29^{2A} (lane 5), *La*GH29^{3A} (lane 6) and *Fb*GH29^{26A} (lane 9). The upper blue dotted line corresponds to the 3FN standard and the lower black dotted line corresponds to the 6FN standard. **C**) H^1 NMR analysis of ATCC_03833^{3A}, *Ri*GH29^{2A}, *La*GH29^{3A} and *Fb*GH29^{26A} reactions with GlcNAc as acceptor and pNP-Fuc as donor. Peaks were assigned by using the appropriate sugar standards. The mid field region allows the distinctive detection of signals of 6FN and 3FN in ATCC_03833^{3A} reaction, 3FN and 4FN in *Ri*GH29^{2A} reaction, 3FN and 6FN in *La*GH29^{3A} reaction and 6FN in *Fb*GH29^{26A} reaction.

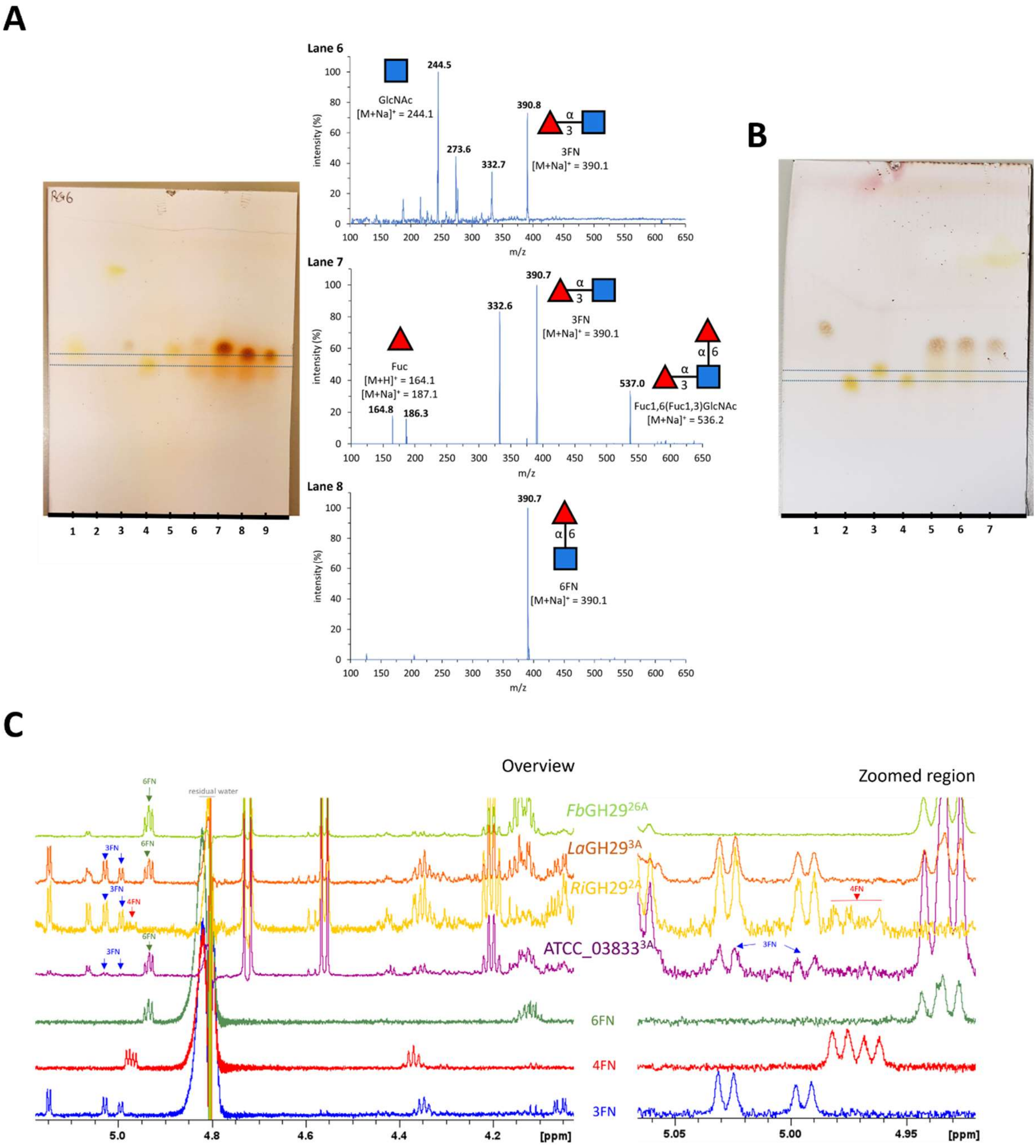
A
B
C
Lane 6
GlcNAc
[M+Na]+ = 244.1
244.5
273.6
332.7
390.8
3FN
[M+Na]+ = 390.1
intensity (%)
m/z
Lane 7
Fuc
[M+H]+ = 164.1
[M+Na]+ = 187.1
164.8
186.3
332.6
390.7
537.0
Fuc1,6(Fuc1,3)GlcNAc
[M+Na]+ = 536.2
Lane 8
6FN
Overview
Zoomed region
residual water
FbGH29^26A
LaGH29^3A
RiGH29^2A
ATCC_03833^3A
6FN
4FN
3FN
[ppm]

Figure S7. Schematic of the GH29BERT semi-supervised training approach used for protein sequence representation and SNN clustering prediction. The training process incorporates a self-supervised pre-training and a supervised task-training. The model first underwent a masked token prediction training, employing a Transformer encoder-based architecture with ~20 M parameters for representation learning to *P* unlabelled GH29 protein sequences ($s_{U1}, s_{U2}, \ldots, s_{UP}$), and acquiring a pretrained model for the conversion of raw sequence data into a low dimensional latent space ($v_1, v_2, \ldots, v_n$). Subsequently, the model was task-trained on Q labelled sequences ($s_{L1}, s_{L2}, \ldots, s_{LQ}$) with known cluster labels derived from the SSN analysis. This supervised phase utilized fundamental self-attention and dense layers with ~0.6 M parameters for fine-tuning. To predict the clustering ID of any given GH29 sequences, sequence representations were generated via the pre-trained model, serving as input for the classifier model.

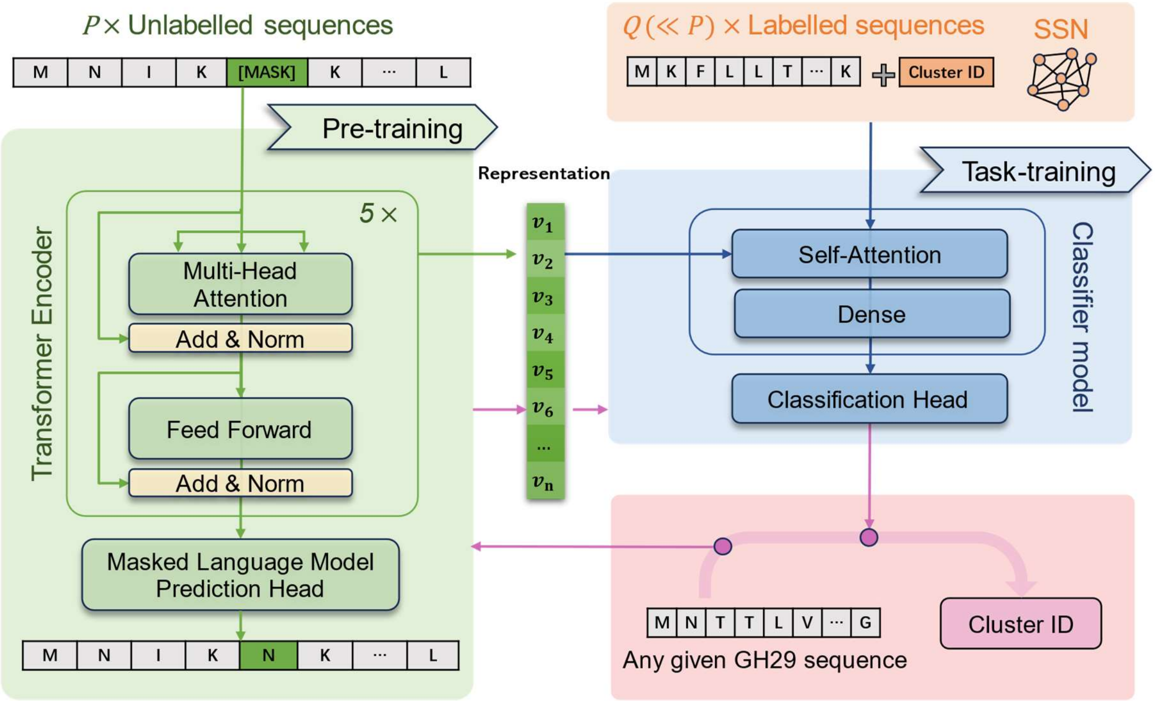

Supplementary References

1. Sano, M., Hayakawa, K. & Kato, I. Purification and characterization of alpha-L-fucosidase from Streptomyces species. J. Biol. Chem. 267, 1522–7 (1992).

2. Shani, G. et al. Fucosylated Human Milk Oligosaccharide Foraging within the Species Bifidobacterium pseudocatenulatum Is Driven by Glycosyl Hydrolase Content and Specificity. Appl. Environ. Microbiol. 88, (2022).

3. Pichler, M. J. et al. Butyrate producing colonic Clostridiales metabolise human milk oligosaccharides and cross feed on mucin via conserved pathways. Nat. Commun. 11, (2020).

4. Grootaert, H., van Landuyt, L., Hulpiau, P. & Callewaert, N. Functional exploration of the GH29 fucosidase family. Glycobiology 30, 735–745 (2020).

5. Moya-Gonzálvez, E. M. et al. Infant Gut Microbial Metagenome Mining of α-L-Fucosidases with Activity on Fucosylated Human Milk Oligosaccharides and Glycoconjugates. Microbiol. Spectr. 10, (2022).

6. Liu, S. et al. The fucosidase-pool of Emticicia oligotrophica: Biochemical characterization and transfucosylation potential. Glycobiology 26, 871–879 (2016).

7. Wu, H. et al. Fucosidases from the human gut symbiont Ruminococcus gnavus. Cell. Mol. Life Sci. 78, 675–693 (2021).

8. Sela, D. A. et al. Bifidobacterium longum subsp. infantis ATCC 15697 α-fucosidases are active on fucosylated human milk oligosaccharides. Appl. Environ. Microbiol. 78, 795–803 (2012).

9. Saumonneau, A. et al. Design of an α-L-transfucosidase for the synthesis of fucosylated HMOs. Glycobiology 26, 261–269 (2015).

10. Shaikh, F. A., Lammerts Van Bueren, A., Davies, G. J. & Withers, S. G. Identifying the catalytic acid/base in GH29 α-L-fucosidase subfamilies. Biochemistry 52, 5857–5864 (2013).

11. Sakurama, H. et al. Differences in the Substrate Specificities and Active-Site Structures of Two α-L-Fucosidases (Glycoside Hydrolase Family 29) from Bacteroides thetaiotaomicron. Biosci. Biotechnol. Biochem. 76, 1022–1024 (2012).

12. Briliūtė, J. et al. Complex N-glycan breakdown by gut Bacteroides involves an extensive enzymatic apparatus encoded by multiple co-regulated genetic loci. Nat. Microbiol. 4, 1571–1581 (2019).

13. Garrido, D. et al. A novel gene cluster allows preferential utilization of fucosylated milk oligosaccharides in Bifidobacterium longum subsp. longum SC596. Sci. Rep. 6, 35045 (2016).

14. Ashida, H. et al. Two distinct α-L-fucosidases from Bifidobacterium bifidum are essential for the utilization of fucosylated milk oligosaccharides and glycoconjugates. Glycobiology 19, 1010–1017 (2009).

15. Zeuner, B. et al. Substrate specificity and transfucosylation activity of GH29 α-L-fucosidases for enzymatic production of human milk oligosaccharides. N. Biotechnol. 41, 34–45 (2018).

16. Fan, S. et al. Cloning, characterization, and production of three α-L-fucosidases from Clostridium perfringens ATCC 13124. J. Basic Microbiol. 56, 347–357 (2016).

17. Hobbs, J. K., Pluvinage, B., Robb, M., Smith, S. P. & Boraston, A. B. Two complementary α-fucosidases from Streptococcus pneumoniae promote complete degradation of host-derived carbohydrate antigens. J. Biol. Chem. 294, 12670–12682 (2019).

18. Zeleny, R. et al. Molecular cloning and characterization of a plant α1,3/4-fucosidase based on sequence tags from almond fucosidase I. Phytochemistry 67, 641–648 (2006).

19. Cobucci-Ponzano, B., Trincone, A., Giordano, A., Rossi, M. & Moracci, M. Identification of an archaeal α-L-fucosidase encoded by an interrupted gene: Production of a functional enzyme by mutations mimicking programmed -1 frameshifting. J. Biol. Chem. 278, 14622–14631 (2003).

20. Curci, N. et al. Xyloglucan oligosaccharides hydrolysis by exo-acting glycoside hydrolases from hyperthermophilic microorganism saccharolobus solfataricus. Int. J. Mol. Sci. 22, (2021).

21. Cao, H., Walton, J. D., Brumm, P. & Phillips, G. N. Structure and substrate specificity of a eukaryotic fucosidase from fusarium graminearum. J. Biol. Chem. 289, 25624–25638 (2014).

22. Schopohl, D. et al. Purification and properties of a secreted and developmentally regulated α-L-fucosidase from Dictyostelium discoideum. J. Biol. Chem. 267, 2400–2405 (1992).

23. Biel-Nielsen, T. L. et al. Utilization of industrial citrus pectin side streams for enzymatic production of human milk oligosaccharides. Carbohydr. Res. 519, 108627 (2022).

24. Zeuner, B., Vuillemin, M., Holck, J., Muschiol, J. & Meyer, A. S. Improved transglycosylation by a xyloglucan-active α-L-fucosidase from fusarium graminearum. J. Fungi 6, 1–19 (2020).

25. Dupoiron, S. et al. The N-Glycan cluster from Xanthomonas campestris pv. campestris: A toolbox for sequential plant N-Glycan processing a toolbox for sequential plant N-Glycan processing. J. Biol. Chem. 290, 6022–6036 (2015).

26. Robb, C. S. et al. Metabolism of a hybrid algal galactan by members of the human gut microbiome. Nat. Chem. Biol. 18, 501–510 (2022).

27. Benešová, E., Lipovová, P., Dvořáková, H. & Králová, B. α-L-fucosidase from paenibacillus thiaminolyticus: Its hydrolytic and transglycosylation abilities. Glycobiology 23, 1052–1065 (2013).

28. Kovaľová, T. et al. Active site complementation and hexameric arrangement in the GH family 29; a structure–function study of α-l-fucosidase isoenzyme 1 from Paenibacillus

thiaminolyticus. Glycobiology 29, 59–73 (2018).

29. Hong, H., Kim, D. H., Seo, H., Kim, K. J. K. H. K. J. & Kim, K. J. K. H. K. J. Dual α-1,4- and β-1,4-Glycosidase Activities by the Novel Carbohydrate-Binding Module in α- l -Fucosidase from Vibrio sp. Strain EJY3. J. Agric. Food Chem. 69, 3380–3389 (2021).

30. Thøgersen, M. S., Christensen, S. J., Jepsen, M., Pedersen, L. H. & Stougaard, P. Transglycosylating β-D-galactosidase and α-L-fucosidase from Paenibacillus sp. 3179 from a hot spring in East Greenland. Microbiologyopen 9, 1–15 (2020).

31. Zhou, W. et al. Discovery and characterization of a novel α-L-fucosidase from the marine-derived Flavobacterium algicola and its application in 2′-fucosyllactose production. Food Chem. 369, 130942 (2021).

32. Lezyk, M. et al. Novel α-L-fucosidases from a soil metagenome for production of fucosylated human milk oligosaccharides. PLoS One 11, 1–18 (2016).

33. Silchenko, A. S. et al. Fucoidan-active α-L-fucosidases of the GH29 and GH95 families from a fucoidan degrading cluster of the marine bacterium Wenyingzhuangia fucanilytica. Arch. Biochem. Biophys. 728, 109373 (2022).

34. Schultz-Johansen, M., Stougaard, P., Svensson, B. & Teze, D. Characterization of five marine family 29 glycoside hydrolases reveals an α-L-fucosidase targeting specifically Fuc(α1,4)GlcNAc. Glycobiology 32, 529–539 (2022).

35. Kostopoulos, I. et al. Akkermansia muciniphila uses human milk oligosaccharides to thrive in the early life conditions in vitro. Sci. Rep. 10, 1–17 (2020).

36. Shuoker, B. et al. Sialidases and fucosidases of Akkermansia muciniphila are crucial for growth on mucin and nutrient sharing with mucus-associated gut bacteria. Nat. Commun. 14, 1833 (2023).

37. Dong, S., Chang, Y., Shen, J., Xue, C. & Chen, F. Purification, expression and characterization of a novel α-L-fucosidase from a marine bacteria Wenyingzhuangia fucanilytica. Protein Expr. Purif. 129, 9–17 (2017).

38. Bishnoi, R., Mahajan, S. & Ramya, T. N. C. An F-type lectin domain directs the activity of Streptosporangium roseum alpha-L-fucosidase. Glycobiology 28, 860–875 (2018).

39. Vainauskas, S. et al. A novel broad specificity fucosidase capable of core α1-6 fucose release from N-glycans labeled with urea-linked fluorescent dyes. Sci. Rep. 8, 9504 (2018).

40. Dawson, G. & Tsay, G. Substrate Specificity of Human α-L-Fucosidase. Arch. Biochem. Biophys. 184, 12–23 (1977).

41. Armstrong, Z., Meek, R. W., Wu, L., Blaza, J. N. & Davies, G. J. Cryo-EM structures of human fucosidase FucA1 reveal insight into substrate recognition and catalysis. Structure 30, 1443-1451.e5 (2022).

42. Intra, J., Concetta, V., Daniela, D. C., Perotti, M. E. & Pasini, M. E. Drosophila sperm surface alpha-L-fucosidase interacts with the egg coats through its core fucose residues. Insect Biochem. Mol. Biol. 63, 133–143 (2015).

43. Paper, J. M. et al. α-Fucosidases with different substrate specificities from two species of Fusarium. Appl. Microbiol. Biotechnol. 97, 5371–5380 (2013).

44. Yamamoto, K., Tsuji, Y., Kumagai, H. & Tochikura, T. Induction and purification of a-L-fucosidase from fusarium oxysporum. Agric. Biol. Chem. 50, 1689–1695 (1986).

45. Shvetsova, S. V. et al. Characterization of a new α-L-fucosidase isolated from Fusarium proliferatum LE1 that is regioselective to α-(1 → 4)-L-fucosidic linkage in the hydrolysis of α-L-fucobiosides. Biochimie 132, 54–65 (2017).

46. Ono, A. et al. Structural investigation of α-L-fucosidase from the pancreas of Patiria pectinifera, based on molecular cloning. Carbohydr. Res. 475, 27–33 (2019).

47. Opheim, D. J. & Touster, O. α-L-Fucosidase from Rat Liver Lysosomes. Methods Enzymol. 50, 505–510 (1978).

48. Bielicki, J., Muller, V., Fuller, M., Hopwood, J. J. & Anson, D. S. Recombinant canine α-L-fucosidase: Expression, purification, and characterization. Mol. Genet. Metab. 69, 24–32 (2000).

49. Berteau, O. et al. α-L-Fucosidases: Exoglycosidases with unusual transglycosylation properties. Biochemistry 43, 7881–7891 (2004).

50. Dicioccio, R. A., Barlow, J. J. & Khushi, L. Substrate Specificity and Other Properties of a-L-Fucosidase from Human Serum. J. Biol. Chem. 257, 714–718 (1982).

51. Nakamura, S., Miyazaki, T. & Park, E. Y. α-L-Fucosidase from Bombyx mori has broad substrate specificity and hydrolyzes core fucosylated N-glycans. Insect Biochem. Mol. Biol. 124, 103427 (2020).

52. Li, T. et al. Identification and characterization of a core fucosidase from the bacterium Elizabethkingia meningoseptica. J. Biol. Chem. 293, 1243–1258 (2018).

53. Pozzo, T., Higdon, S. M., Pattathil, S., Hahn, M. G. & Bennett, A. B. Characterization of novel glycosyl hydrolases discovered by cell wall glycan directed monoclonal antibody screening and metagenome analysis of maize aerial root mucilage. PLoS One 13, 1–19 (2018).

54. Liu, P. et al. Screening and characterization of an α-L-fucosidase from Bacteroides fragilis NCTC9343 for synthesis of fucosyl-N-acetylglucosamine disaccharides. Appl. Microbiol. Biotechnol. 104, 7827–7840 (2020).

55. Osanjo, G. et al. Directed evolution of the α-L-fucosidase from Thermotoga maritima into an α-L-transfucosidase. Biochemistry 46, 1022–1033 (2007).

56. Sulzenbacher, G. et al. Crystal structure of Thermotoga maritima α-L-fucosidase: Insights into the catalytic mechanism and the molecular basis for fucosidosis. J. Biol. Chem. 279, 13119–13128 (2004).

57. Ashida, H. et al. 1,6-α-L-Fucosidases from Bifidobacterium longum subsp. infantis ATCC 15697 Involved in the Degradation of Core-fucosylated N-Glycan. J. Appl. Glycosci. 67, 23–29 (2020).

58. Megson, Z. A. et al. Characterization of an α-L-fucosidase from the periodontal pathogen Tannerella forsythia. Virulence 6, 282–292 (2015).

59. Shi, R. et al. Biochemical characterization of a novel α-L-fucosidase from Pedobacter sp. and its application in synthesis of 3′-fucosyllactose and 2′-fucosyllactose. Appl. Microbiol. Biotechnol. 104, 5813–5826 (2020).

60. Bueren, A. L. Van et al. Analysis of the reaction coordinate of alpha-L-fucosidases: a combined structural and quantum mechanical approach. J. Am. Chem. Soc. 132, 1804–6 (2010).

61. Rodríguez-Díaz, J., Monedero, V. & Yebra, M. J. Utilization of natural fucosylated oligosaccharides by three novel α-L-fucosidases from a probiotic lactobacillus casei strain. Appl. Environ. Microbiol. 77, 703–705 (2011).

62. Rodríguez-Díaz, J., Carbajo, R. J., Pineda-Lucena, A., Monedero, V. & Yebra, M. J. Synthesis of fucosyl-N-Acetylglucosamine disaccharides by transfucosylation using α-L-Fucosidases from Lactobacillus casei. Appl. Environ. Microbiol. 79, 3847–3850 (2013).

63. Becerra, J. E. et al. Unique microbial catabolic pathway for the human core n-glycan constituent fucosyl-α-1,6-N-acetylglucosamine-asparagine. MBio 11, 1–18 (2020).

64. Klontz, E. H. et al. Structure and dynamics of an α-fucosidase reveal a mechanism for highly efficient IgG transfucosylation. Nat. Commun. 11, 1–14 (2020).

65. Lin, Z. et al. Evolutionary-scale prediction of atomic-level protein structure with a language model. Science. 379, 1123–1130 (2023).

66. Elnaggar, A. et al. ProtTrans: Toward Understanding the Language of Life Through Self-Supervised Learning. IEEE Trans. Pattern Anal. Mach. Intell. 44, 7112–7127 (2022).
