# Supplementary material for: Exploring the sequence-function space of microbial fucosidases
Source: Commun Chem. 2024 Jun 18;7:137. doi: 10.1038/s42004-024-01212-4 (PMC11189522; doi:10.1038/s42004-024-01212-4)
Supplement: Supplementary file 2 — Description of Additional Supplementary Files [file 42004_2024_1212_MOESM2_ESM.pdf]

Description of Additional Supplementary Files

File name: Supplementary data 1.

File Description: PDB file for D218N Apo (8P1S).

File name: Supplementary data 2.

File Description: PDB file for Fuc-bound WT (8P1R).

File name: Supplementary data 3.

File Description: Taxonomy and clustering distribution of 34,258 non-redundant GH29 708 sequences.

File name: Supplementary data 4.

File Description: Sequence representations of GH29 family color-coded by SSN cluster ID 710 allocation (maximum resolution).

File name: Supplementary data 5.

File Description: sequences of 2971 GH29s in the SSN

File name: Supplementary data 6.

File Description: sequences of 2796 GH29s for task training

File name: Supplementary data 7.

File Description: Configuration of running environment for GH29BERT.
