# Supplementary material for: Exploring the sequence-function space of microbial fucosidases
Source: Commun Chem. 2024 Jun 18;7:137. doi: 10.1038/s42004-024-01212-4 (PMC11189522; doi:10.1038/s42004-024-01212-4)
Supplement: Supplementary file 10 — Reporting Summary [file 42004_2024_1212_MOESM10_ESM.pdf]

nature portfolio

Corresponding author(s): *Double-anonymous peer review submissions: write DAPR and your manuscript number here instead of author names.*

Last updated by author(s): *YYYY-MM-DD*

Reporting Summary

Nature Portfolio wishes to improve the reproducibility of the work that we publish. This form provides structure for consistency and transparency in reporting. For further information on Nature Portfolio policies, see our Editorial Policies and the Editorial Policy Checklist.

Statistics

For all statistical analyses, confirm that the following items are present in the figure legend, table legend, main text, or Methods section.

n/a	Confirmed	
☐	☒	The exact sample size (*n*) for each experimental group/condition, given as a discrete number and unit of measurement
☐	☒	A statement on whether measurements were taken from distinct samples or whether the same sample was measured repeatedly
☒	☐	The statistical test(s) used AND whether they are one- or two-sided<br>*Only common tests should be described solely by name; describe more complex techniques in the Methods section.*
☒	☐	A description of all covariates tested
☒	☐	A description of any assumptions or corrections, such as tests of normality and adjustment for multiple comparisons
☐	☒	A full description of the statistical parameters including central tendency (e.g. means) or other basic estimates (e.g. regression coefficient) AND variation (e.g. standard deviation) or associated estimates of uncertainty (e.g. confidence intervals)
☒	☐	For null hypothesis testing, the test statistic (e.g. *F*, *t*, *r*) with confidence intervals, effect sizes, degrees of freedom and *P* value noted<br>*Give P values as exact values whenever suitable.*
☒	☐	For Bayesian analysis, information on the choice of priors and Markov chain Monte Carlo settings
☒	☐	For hierarchical and complex designs, identification of the appropriate level for tests and full reporting of outcomes
☒	☐	Estimates of effect sizes (e.g. Cohen's *d*, Pearson's *r*), indicating how they were calculated

Our web collection on statistics for biologists contains articles on many of the points above.

Software and code

Policy information about availability of computer code

Data collection	We didn't use any commercial, open source or custom code to collect the data in this study.
Data analysis	As mentioned in the Methods, we used Cytoscape 3.9.1 for sequence similarity network analysis, Prism 5 (GraphPad) for kinetic parameters analysis, Advion Mass Express software for TLC-MS analysis, xia2, dials, Chainsaw, ArpWarp, coot, refmac and PDBredo were used for crystallography analysis.

For manuscripts utilizing custom algorithms or software that are central to the research but not yet described in published literature, software must be made available to editors and reviewers. We strongly encourage code deposition in a community repository (e.g. GitHub). See the Nature Portfolio guidelines for submitting code & software for further information.

Data

Policy information about availability of data

All manuscripts must include a data availability statement. This statement should provide the following information, where applicable:

- Accession codes, unique identifiers, or web links for publicly available datasets
- A description of any restrictions on data availability
- For clinical datasets or third party data, please ensure that the statement adheres to our policy

The datasets generated and/or analyzed during the current study are available from the corresponding author on reasonable request. Crystal structure data were submitted to PDB database with identifiers as 8P1S (supplementary data 1) and 8P1R (supplementary data 2). More data can be found in supplementary

information. Supplementary data includes:
Supplementary data 1. PDB file for D218N Apo (8P1S).
Supplementary data 2. PDB file for Fuc-bound WT (8P1R).
Supplementary data 3. Taxonomy and clustering distribution of 34,258 non-redundant GH29 sequences.
Supplementary data 4. Sequence representations of GH29 family color-coded by SSN cluster ID allocation (maximum resolution).
Supplementary data 5. sequences of 2971 GH29s in the SSN
Supplementary data 6. sequences of 2796 GH29s for task training
Supplementary data 7. Configuration of running environment for GH29BERT.

Research involving human participants, their data, or biological material

Policy information about studies with human participants or human data. See also policy information about sex, gender (identity/presentation), and sexual orientation and race, ethnicity and racism.

Reporting on sex and gender	There are no human participants or human data in this study.
Reporting on race, ethnicity, or other socially relevant groupings	There are no human participants or human data in this study.
Population characteristics	There are no human participants or human data in this study.
Recruitment	There are no human participants or human data in this study.
Ethics oversight	There are no human participants or human data in this study.

Note that full information on the approval of the study protocol must also be provided in the manuscript.

Field-specific reporting

Please select the one below that is the best fit for your research. If you are not sure, read the appropriate sections before making your selection.

☒ Life sciences ☐ Behavioural & social sciences ☐ Ecological, evolutionary & environmental sciences

For a reference copy of the document with all sections, see nature.com/documents/nr-reporting-summary-flat.pdf

Life sciences study design

All studies must disclose on these points even when the disclosure is negative.

Sample size	For sequence similarity networks (SSN) analysis, 9,505 GH29 sequences were extracted from CAZy database (www.cazy.org)(last update 2022-10-18) . The sample size of 9,505 was chosen because this was the total number of GH29 sequences of CAZy database at data acquisition stage.
Data exclusions	To avoid sequence redundancy, 6,534 out of 9,505 sequences sharing over 80% sequence identity to the remaining sequences (2,971) were excluded by CD-HIT suite, which is a pre-established tool.
Replication	Regarding enzymatic assays, technical triplicates of sample preparation and analysis were preformed to verify the reproducibility of the experimental findings. All attempts at replication were successful.
Randomization	Samples were randomly allocated into experimental groups.
Blinding	The investigators were not blinded to group allocation during data collection or analysis in order to provide a comprehensive family exploration of GH29 fucosidases.

Reporting for specific materials, systems and methods

We require information from authors about some types of materials, experimental systems and methods used in many studies. Here, indicate whether each material, system or method listed is relevant to your study. If you are not sure if a list item applies to your research, read the appropriate section before selecting a response.

Materials & experimental systems

n/a	Involved in the study
☒	☐ Antibodies
☒	☐ Eukaryotic cell lines
☒	☐ Palaeontology and archaeology
☒	☐ Animals and other organisms
☒	☐ Clinical data
☒	☐ Dual use research of concern
☒	☐ Plants

Methods

n/a	Involved in the study
☒	☐ ChIP-seq
☒	☐ Flow cytometry
☒	☐ MRI-based neuroimaging

Plants

Seed stocks	There are no seed stocks or other plant material used in this study.
Novel plant genotypes	There are no seed stocks or other plant material used in this study.
Authentication	There are no seed stocks or other plant material used in this study.
